# A highly multiplexed quantitative phosphosite assay for biology and preclinical studies

**DOI:** 10.15252/msb.202010156

**Published:** 2021-09-27

**Authors:** Hasmik Keshishian, E Robert McDonald, Filip Mundt, Randy Melanson, Karsten Krug, Dale A Porter, Luke Wallace, Dominique Forestier, Bokang Rabasha, Sara E Marlow, Judit Jane‐Valbuena, Ellen Todres, Harrison Specht, Margaret Lea Robinson, Pierre M Jean Beltran, Ozgun Babur, Meagan E Olive, Javad Golji, Eric Kuhn, Michael Burgess, Melanie A MacMullan, Tomas Rejtar, Karen Wang, DR Mani, Shankha Satpathy, Michael A Gillette, William R Sellers, Steven A Carr

**Affiliations:** ^1^ Broad Institute of Massachusetts Institute of Technology and Harvard Cambridge MA USA; ^2^ Novartis Institute of Biomedical Research Cambridge MA USA; ^3^ Computer Science Department University of Massachusetts Boston Boston MA USA; ^4^ Division of Pulmonary and Critical Care Medicine Massachusetts General Hospital Boston MA USA; ^5^ Department of Medical Oncology Dana‐Farber Cancer Institute and Harvard Medical School Boston MA USA; ^6^ Present address: Novo Nordisk Foundation Center for Protein Research Faculty of Health Sciences University of Copenhagen Copenhagen Denmark; ^7^ Present address: Department of Oncology and Pathology Science for Life Laboratory Karolinska Institutet Stockholm Sweden; ^8^ Present address: Cedilla Therapeutics Cambridge MA USA

**Keywords:** breast cancer, CPTAC, medulloblastoma, post‐translational modifications, targeted mass spectrometry, Cancer, Proteomics, Signal Transduction

## Abstract

Reliable methods to quantify dynamic signaling changes across diverse pathways are needed to better understand the effects of disease and drug treatment in cells and tissues but are presently lacking. Here, we present SigPath, a targeted mass spectrometry (MS) assay that measures 284 phosphosites in 200 phosphoproteins of biological interest. SigPath probes a broad swath of signaling biology with high throughput and quantitative precision. We applied the assay to investigate changes in phospho‐signaling in drug‐treated cancer cell lines, breast cancer preclinical models, and human medulloblastoma tumors. In addition to validating previous findings, SigPath detected and quantified a large number of differentially regulated phosphosites newly associated with disease models and human tumors at baseline or with drug perturbation. Our results highlight the potential of SigPath to monitor phosphoproteomic signaling events and to nominate mechanistic hypotheses regarding oncogenesis, response, and resistance to therapy.

## Graphical abstract

SigPath is a targeted, quantitative mass spectrometry assay that measures 284 phosphosites spanning 200 phosphoproteins with high throughput and quantitative precision across a broad swath of signaling biology of known interest.

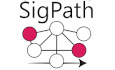

## Introduction

Cellular processes including signal transduction, cell cycle progression, and response to DNA damage, among many others, are regulated through the addition or removal of phosphate from the amino acids serine, threonine, and tyrosine. In keeping with this, aberrant phospho‐signaling is a hallmark of many diseases including cancer. For example, genetic disruption of the tumor suppressors PTEN and APC leads to pathologic levels of phosphorylated AKT and ß‐catenin respectively, while oncogenic activation of ABL and RAS leads to aberrant phosphorylation in CRKL or MEK, respectively. Dysregulated kinases and phosphatases have thus become important targets for therapeutic development. This in turn has motivated the desire to quantitatively monitor phosphorylation events to determine the cellular or organismal activity of such inhibitors. Unfortunately, due to the limited ability to robustly quantify hundreds of phosphorylation events, most drug discovery programs in this area have followed single phosphorylation events as the marker of pharmacodynamic activity. As a result, paradoxical activation of RAF isoforms as a consequence of BRAF inhibitors (Hatzivassiliou *et al*, 2010; Poulikakos *et al*, 2010), or feedback upstream pathway activation resulting from mTOR inhibitors, was missed until well after the relevant molecules were in clinical trials or beyond (Shi *et al*, 2005).

The Cancer Cell Line Encyclopedia project (CCLE), in addition to characterizing genome, transcriptome, and methylome alterations (Barretina *et al*, 2012; Ghandi *et al*, 2019), has sought to characterize the metabolome and proteome across hundreds of cancer cell lines (Li *et al*, 2019; Nusinow *et al*, 2020). An initial attempt in the phosphoproteome space was also made using Reverse Phase Protein Arrays (Li *et al*, 2017); however, the sparse availability of phosphoantibodies that are highly reliable in detecting phosphorylation events on the protein arrays limits the broader application of this approach. Thus, to begin to develop high‐complexity quantitative phosphoprotein assays for use in both characterizing cell lines and clinical samples and to enable much deeper pharmacodynamic assessment of therapeutics, we set out to develop robust mass spectrometry‐based phospho‐assay sets.

Mass spectrometry (MS)‐based proteomics has led to the discovery of the majority of the over 200,000 known human phosphosites (Phosphosite.org; Hornbeck *et al*, 2015). Many laboratories, but especially those associated with the Clinical Proteomics Tumor Analysis Consortium (Rodriguez *et al*, 2021), have elaborated deep, high‐quality phosphopeptide, and proteome datasets (Zhang *et al*, 2014, 2016; Mertins *et al*, 2016; Chen *et al*, 2017a; Huang *et al*, 2017; Archer *et al*, 2018; Vasaikar *et al*, 2019; Dou *et al*, 2020; Gillette *et al*, 2020; Krug *et al*, 2020; Satpathy *et al*, 2020). The deepscale proteomic methods used in the CPTAC studies detect 30,000–45,000 distinct phosphosites in each sample studied, and quantitative chemical labeling provides relative quantification of each site across samples. However, one drawback of this approach is the lack of uniform detection of any given phosphosite across an entire sample cohort, a technical artifact caused by stochastic sampling of the analytes introduced into the MS system, especially those in low abundance, and the extreme complexity of the samples analyzed.

Targeted MS in the forms of multiple reaction monitoring (MRM, also referred to as selected reaction monitoring, SRM) and parallel reaction monitoring (PRM) is now widely used for highly multiplexed, quantitative measurement of proteins in blood, cells, and tissues (Keshishian *et al*, 2009; Kuhn *et al*, 2009; Picotti & Aebersold, 2012; Rebecca *et al*, 2014; Soste *et al*, 2014; Gallien *et al*, 2015; Abelin *et al*, 2016; Chen *et al*, 2017b; Manes & Nita‐Lazar, 2018; Whiteaker *et al*, 2018; Huttenhain *et al*, 2019; Sperling *et al*, 2019; Eshghi *et al*, 2020; Pino *et al*, 2020). All variants of the approach begin with targeted selection in the mass spectrometer of the intact, ionized peptides of interest followed by fragmentation of each peptide precursor to produce product ions that, together with the mass of the intact peptide, are used to identify and quantify that peptide. In its most specific and precise form, heavy isotope‐labeled synthetic peptides are added at known concentration to verify that the correct peptide is being measured and to improve accuracy of the relative quantification of the target peptide. The use of this technology to measure post‐translationally modified peptides is less common. Targeted MS assays have largely been developed for purposes of verification; however, if such an assay queries a large number of targets, it can also be viewed as a discovery method.

In contrast to multiplexed antibody methods, MS‐based targeted analysis of peptides and modified peptides, including phosphopeptides, can, in principle, be configured to quantify any phosphosite of interest in any organism and scaled to measure many hundreds of peptides in a single measurement cycle of a few hours by liquid chromatography–tandem mass spectrometry (LC‐MS/MS; Burgess *et al*, 2014). Soste and coworkers developed targeted MS assays to 152 phosphosites and 157 proteins in yeast that were culled from the literature to develop what they called “sentinel markers'' to give biological insights (Soste *et al*, 2014). The methods have been extended to mammalian systems in several recent studies to detect and quantify on the order of 100 phosphosites in a single 1‐ to 2‐h analysis (Abelin *et al*, 2016; Kennedy *et al*, 2016). These studies employed TiO2 or immobilized metal affinity chromatography (IMAC) to enrich phosphopeptides from cells followed by analysis of the resulting mixture by targeted MS using heavy stable isotope‐labeled (SIL) phosphopeptides for confident detection and quantification. Kennedy *et al*. measured phosphosites relevant to DNA damage response, while Abelin *et al*. assayed a set of moderate‐to‐high‐abundance phosphopeptides known to be modulated in non‐uniform ways by a panel of drugs in a range of cell lines. The targets were selected based on ease of detection in a small number of initial chemical or genomic perturbations in cell lines, not on their potential biological relevance. Recently, Stopfer *et al*. developed and applied a targeted MS assay for several hundred phosphotyrosine (pY) sites and were able to consistently detect and quantify 165 endogenous pY peptides in colorectal cancer tumor samples (Stopfer *et al*, 2021).

Here, we present the development and application of SigPath, one of the largest phosphosite assay panels to date applicable to mammalian cells and human tissues. This quantitative, targeted MS‐based assay measures 284 phosphosites in 200 cancer‐relevant target proteins spanning many pathways. Phosphosites were selected by cancer biologists based on known or presumed relevance to cancer disease or treatment and through discovery proteomics efforts leading to the SigPath set of 298 phosphopeptides. This unique set is purposely designed to probe a broad swath of signaling biology in a single measurement rather than focusing on a single pathway. Importantly, the panel can be extended to measure other phosphosites in additional pathways as desired. Here, we demonstrate the utility of the assay through a range of applications in drug‐treated cell lines, preclinical models of breast cancer and human medulloblastoma tumor samples.

## Results

### Selection of phosphosites for assay development and assay construction

**Figure EV1:**
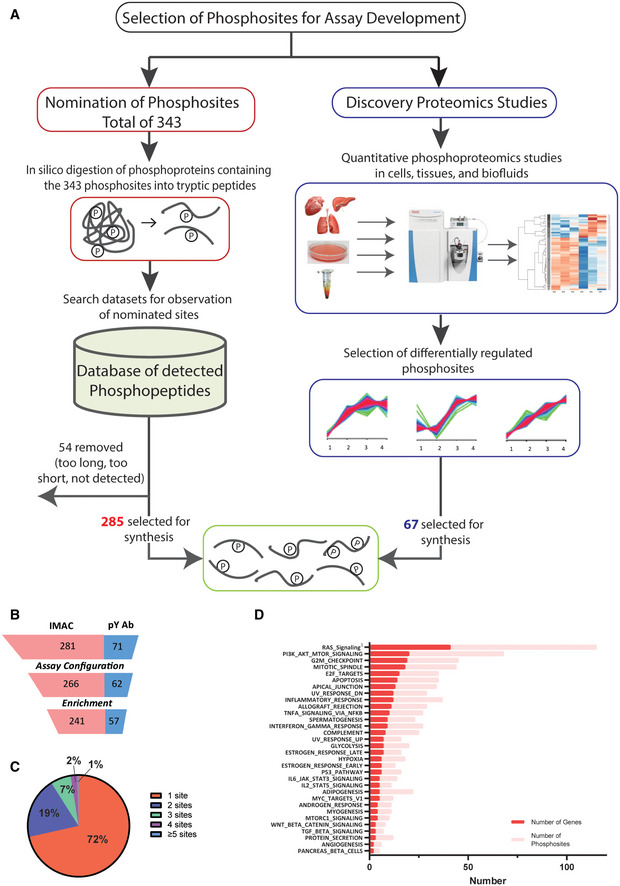
Development of SigPath assay AProcess for selecting phosphosites and phosphopeptides for SigPath assay development. Majority of phosphosites were nominated by experts, then converted into tryptic peptides, searched against existing datasets at the Broad for detection of them in MS data (see Materials and Methods section). One‐fourth of the phosphosites were included based on them being differentially regulated in quantitative phosphoproteomic studies (Mertins *et al*, 2014). Once finalized [C13, N15], stable isotope‐labeled versions of the peptides were synthesized for the assay.BAssay configuration and testing statistics of SigPath. Twenty‐four out of 352 peptides failed the assay configuration due to their LC or MS characteristics, while another 30 failed during the pY Ab or IMAC enrichment step. Final SigPath assay targets 298 phosphopeptides with 284 phosphosites.CPie graph showing range of phosphosites per protein in the assay panel. 71% of the proteins are represented by only one phosphopeptide, 19% by two phosphopeptides. The remaining varies from 3 to 9 phosphopeptides.DPathways represented by SigPath in MSigDB Hallmark pathway category. To be included in the plot, a pathway had to have at least 5% coverage, or be represented by a minimum of three proteins and five phosphosites in the assay. Both, number of genes (red) and phosphosites (pink) are shown on the plot. ^1^Included from MSigDB WikiPathway pathway category.

The majority of the phosphosites were nominated by cancer biologists in our institutions and supplemented with frequently modulated phosphosites observed in our discovery proteomic experiments (Fig EV1A). In an effort to further characterize the signaling and physiological state of human tumor samples, we set out to capture nodes of biological pathways known to be modulated via phosphorylation. Critical kinase cascades, often hyperactivated in tumors, make up the backbone of the assay set. These include the MAPK, PI3K, PKC, SRC, and JAK signaling pathways as well as both receptor and non‐receptor tyrosine kinases. Both critical activation sites of the kinases themselves and their relevant downstream substrates were targeted. These kinase cascades often culminate in the modulation of transcription factor circuits, so transcriptional nodes within the FOXO, STAT, NFKB, TGFB, and Wnt pathways were also selected. Lastly, to capture physiological cell states, protein phosphosites were selected to provide readouts on DNA damage, cell cycle arrest, apoptosis, spindle checkpoint activation, hypoxia, autophagy, cell stress, and epithelial‐to‐mesenchymal transition. The rationale for specific target selection and pathway information for the selected sites is presented in Datasets EV1 and EV2, respectively. While these sites were selected based on relevance to cancer cell‐autonomous phenotypes, the associated pathways constitute central signaling nodes and should therefore be valuable when applied to a variety of experimental paradigms.

### SigPath assay development

Assay development, configuration, and quality assessment, as well as workflow development (Figs EV1A and 1A), are described in Materials and Methods. Briefly, spectral libraries were generated on a QE or QE plus mass spectrometer using synthetic, SIL peptides, and transitions selected and optimized on a TSQ Quantiva Triple Quadrupole Mass Spectrometer. The final assay begins with proteolytic digestion of the sample, followed by spiking of SIL peptides into digested sample and subsequent phosphopeptide enrichment steps. We first perform phosphotyrosine antibody (pY Ab) capture, followed by immobilized metal affinity chromatography (IMAC) on the flow‐through of pY Ab capture. Phosphotyrosine Ab and IMAC eluents were analyzed using an LC‐MRM/MS method on two‐ and three‐hour gradients, respectively. The overall success rate for detection of the targeted heavy phosphopeptides was 85% (Fig EV1B). Twenty‐four out of 352 phosphopeptides failed assay configuration due to either their instability in solution or their poor behavior on LC or MS, while an additional 30 peptides failed during the pY Ab or IMAC enrichment steps (Dataset EV1). It is noteworthy that of the 37 phosphopeptides that were included in the assay panel but lacked prior experimental observation in our datasets (mostly pY‐containing), we were able to successfully configure assays for 24. The final working assay contains 298 phosphopeptides representing 284 phosphosites and 200 phosphoproteins (Table 1 and Dataset EV1). In addition, 178 of the 298 phosphopeptides (60%) are fully conserved between humans and mouse; therefore, this portion of the assay can be applied to mouse samples. The SigPath panel predominantly consists of singly phosphorylated peptides, with only 8 doubly phosphorylated phosphopeptides included. The majority of phosphoproteins (142/200) are represented by a single tryptic phosphopeptide containing a single phosphosite (Fig EV1C).

**Figure 1:**
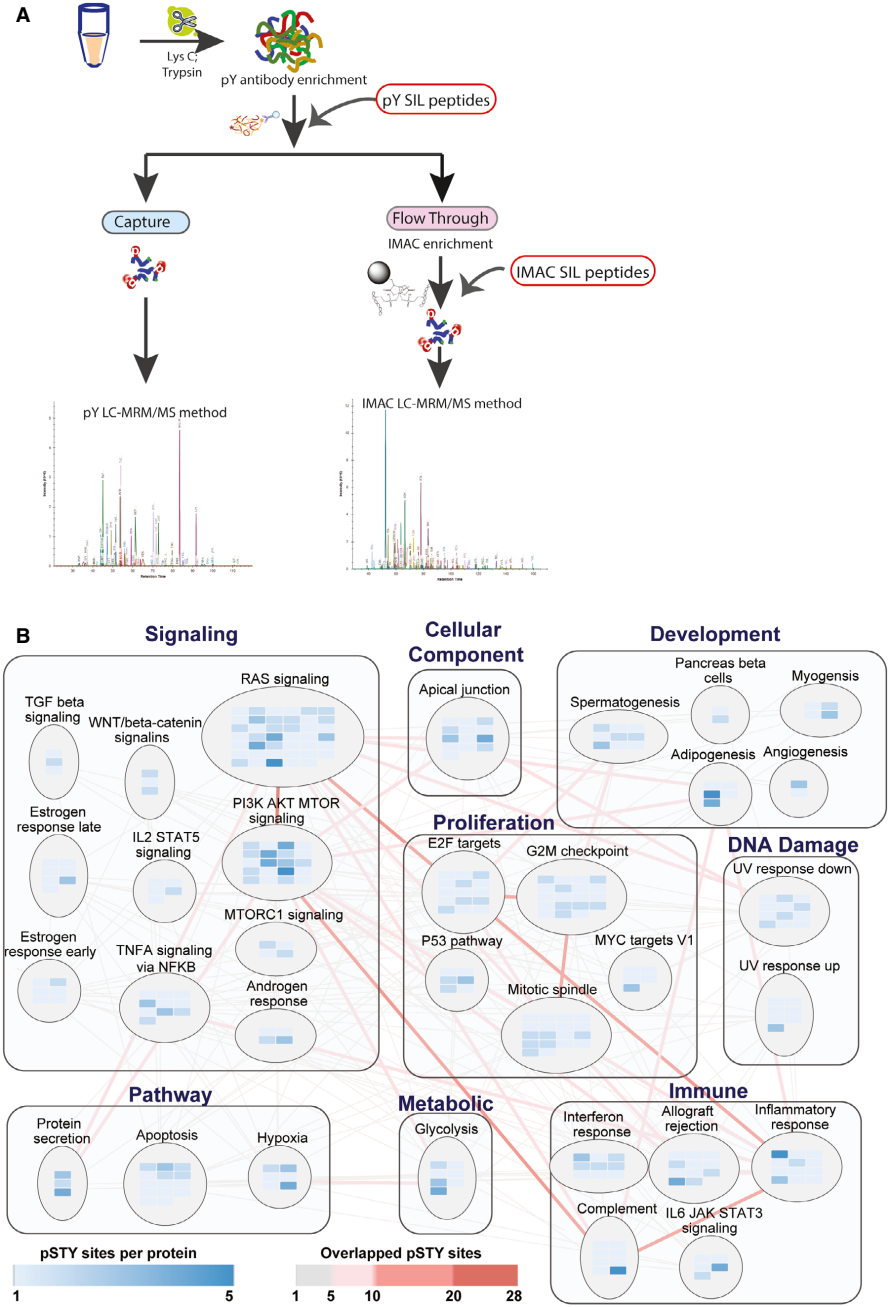
SigPath assay workflow and pathway coverage AIn the full SigPath workflow, the heavy stable isotope‐labeled (SIL) pY peptide set is spiked into the digested sample and endogenous and spiked SIL peptides enriched using pY antibody. A portion of the flow‐through from the pY enrichment is then spiked with the IMAC set of SIL peptides and enriched by IMAC. Both, pY Ab and IMAC‐captured samples are analyzed on the MS using pY and IMAC LC‐MRM/MS methods, respectively (see Materials and Methods).BMSigDB Hallmark gene sets and process categories (Liberzon *et al*, 2015) represented by SigPath. To be included in the plot, a pathway had to have at least 5% coverage, or be represented by a minimum of three proteins and five phosphosites in the assay. The RAS signaling pathway from WikiPathway (Martens *et al*, 2021) is also included in the plot. Each rectangle assembles gene sets in the same process category. Gene set is shown in circles in which blue colored rectangles refer to the proteins represented in the assay. Shades of blue indicate the number of phosphosites per protein in the assay. Edges show overlapping proteins and phosphosites between the different gene sets. Overlap of 1–5 phosphosites is indicated with gray lines, whereas overlap of more than 5 phosphosites is shown with red lines at increasing intensity. The larger the overlap, the more intense is the shade of red.

**Table 1:** List of all the proteins in the final SigPath assay panel

HUGO‐approved symbol	Number of phosphosites	HUGO‐approved symbol	Number of phosphosites	HUGO‐approved symbol	Number of phosphosites	HUGO‐approved symbol	Number of phosphosites
ABI1	1	FGFR1	1	MAPRE1	1	RBM39	1
ABL1	1	FGFR2	1	MARCKS	2	RBM7	1
ACIN1	1	FGFR3	3	MARVELD2	1	RELA	1
AKT1	2	FGFR4	1	MAST2	1	RET	1
AKT1S1	1	FLT3	1	MCL1	2	RICTOR	2
AKT2	1	FOXO1	2	MDM2	1	RIPK1	2
AKT3	1	FOXO3	2	MET	1	ROS1	1
ALK	3	FRS2	3	MITF	1	RPS27	1
ANLN	1	FRS3	1	MOB1A	1	RPS6	3
APLP2	1	GAB1	1	MPZL1	1	RPS6KA1	4
ARAF	4	GLYR1	1	MTOR	3	RPS6KA2	1
ATM	1	GRB2	1	MYC	1	RPS6KA3	3
AURKA	1	GSK3A	1	MYCBP2	1	RPS6KB1	2
AURKB	1	GSK3B	2	NF2	1	RREB1	1
AXL	1	GTF2I	1	NFKB1	1	SHB	1
Bad	2	HECTD1	1	NFKB1	1	SHC1	2
BCL2	1	HGS	1	NFKBIA	3	SMAP	1
BCL2L11	1	HIF1A	2	NTRK1	1	SMARCA4	1
BRAF	2	HMGN1	1	NTRK2	1	SMC4	1
BRCA1	1	HNRNPA3	1	NUCKS1	1	SOCS3	1
BTK	2	HSPBL2	1	PAK1	1	SOS1	2
BUB1	1	IGF1R	2	PALLD	1	SPRY1	1
CAMSAP2	1	INSR	1	PBRM1	1	SPRY2	3
CDC20	1	IRF3	1	PDPK1	1	SPRY4	1
CDK1	2	IRS1	2	PEA15	1	SRC	1
CFAP97	1	IRS2	3	PGAM1	1	STAT3	2
Chek1	2	JAK1	1	PHIP	1	STAT5A	1
CIC	1	JAK2	1	PIK3R1	3	STK3	1
CPSF6	1	JUN	1	PLCB3	1	STK39	1
CRK	1	JUND	1	PLCG1	1	STK4	2
CTNNB1	2	KIF2C	1	PLCG2	1	STMN1	1
DDI2	1	KIF4A	1	PLK1	2	SUFU	1
DNAH5	1	KIT	1	PPP2R5B	1	SYK	1
DOCK5	1	KMT2A	1	PRKAA1	1	SYNPO2	1
DOT1L	1	KSR1	2	PRKACA	1	TAZ	1
DYRK2	1	LAG3	1	PRKCA	1	TP53	2
EGFR	4	LATS1	2	PRKCB	2	TP53BP1	1
EIF2A	2	LATS1	2	PRKCB	1	TPX2	1
EIF2AK4	1	LATS2	1	PRKCD	1	TSC2	1
EIF4EBP1	3	LCK	1	PRKCQ	1	TTC39B	1
EML4	1	LYN	1	PRKCZ	1	UBE2J1	1
EPAS1	1	MAP2K1	2	PRKD2	1	ULK1	1
EPHA2	1	MAP2K4	1	PRKDC	1	VAV1	1
EPS8	1	MAP3K14	2	PTEN	1	VEPH1	1
ERBB2	2	MAP3K7	1	PTK2	3	VIM	1
ERBB3	2	MAPK1	2	PTPN11	4	XIAP	1
ERBB4	1	MAPK14	2	RAB11B	1	YAP1	2
ERH	1	MAPK3	2	RAF1	9	YTHDC1	1
ERRFI	1	MAPK8	2	RAF1	1	ZAP70	1
EZH2	2	MAPK9	1	RB1	3	ZNF638	1

Table includes HUGO gene names and number of phosphosites per protein in the assay panel.

### Pathway representation in the final assay panel

Pathways represented by the final SigPath panel were assessed using the canonical databases in the Molecular Signatures Database (MSigDB; Kanehisa & Goto, 2000; Pico *et al*, 2008; Schaefer *et al*, 2009; Liberzon *et al*, 2011, 2015; Jassal *et al*, 2020). The panel represents a spectrum of cancer‐relevant biology spanning signal transduction, cell proliferation, apoptosis, and the immune system (Dataset EV2). Figures 1B and EV1D illustrate Hallmark gene sets and processes represented in SigPath with the addition of the Ras pathway from WikiPathways (Martens *et al*, 2021).

### SigPath assay configuration and evaluation

**Figure EV2:**
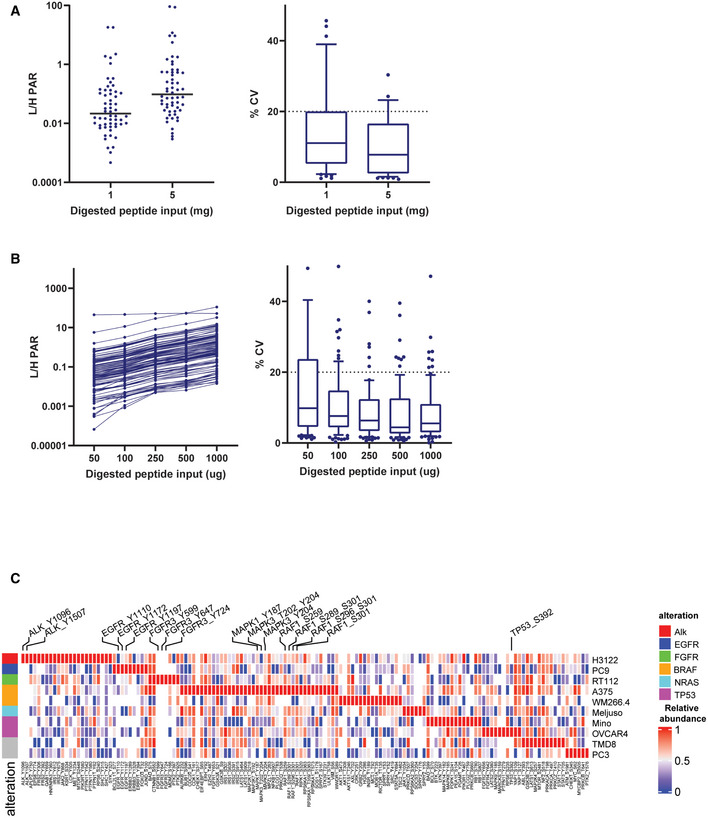
Evaluation of the SigPath assay AAverage light/heavy peak area ratio and %CV of the three replicates for all quantified peptides in titration curve experiment after pY Ab enrichment. Plot on the left shows the average values for all the quantified peptides along with a line representing the median value at 1 and 5 mg input protein level. The plot on the right shows the spread of %CVs at 1 and 5 mg input protein. The box represents interquartile range (IQR) with the lower, central, and the upper bands representing 25^th^ percentile (Q1), median, and 75^th^ percentile (Q3), respectively. The whiskers extend from 10 to 90 percentile of the data.BAverage light/heavy peak area ratio and %CV of the three replicates for all quantified peptides in titration curve experiment after IMAC enrichment. Plot on the left shows the average values for all the quantified peptides along with a line representing the median value at 0.05, 0.1, 0.25, 0.5, and 1 mg input protein level. The plot on the right shows the spread of %CVs at all the input protein levels. The box represents interquartile range (IQR) with the lower, central, and upper bands representing 25^th^ percentile (Q1), median, and 75^th^ percentile (Q3), respectively. The whiskers extend from 10 to 90 percentile of the data.CHeat map showing relative abundances of all detected phosphosites across the 10 cell lines. Labeled are phosphosites on known driver genes in these cell lines. One replicate of each samples was processed and analyzed for this experiment. The heat map was generated using Morpheus online tool, the data are median‐MAD normalized, and colors are relative across rows, from row min to row max.

Evaluation of sensitivity and reproducibility of the assay were performed in a mixture of five cancer cell lines (OVCAR, Meljuso, H3122, PC9, A375) to maximize detection of endogenous peptides. Phosphotyrosine Ab enrichment was performed with two peptide input amounts (1 and 5 mg), whereas IMAC enrichment was performed with five different input amounts ranging from 0.05 to 1 mg. Fifty‐eight and 96 endogenous light peptides were detected in this experiment with median coefficient of variation (CV) of less than 20% after pY Ab and IMAC enrichments, respectively. Phosphotyrosine Ab enrichment with only 1 mg input achieved quantification of 52 (93%) of the detected peptides, while IMAC enrichment achieved quantification of 74 (77%) of the detected peptides in the lowest input, 0.05 mg sample (Fig EV2A and B).

We initially evaluated the assay in ten cell lines representing various cancer types (lung, B‐cell lymphoma, mantle cell lymphoma, prostate, ovarian, bladder, and melanoma) and genetic contexts using 250 of the 298 phosphopeptides in SigPath (Dataset EV3). Each cell line was selected to maximize the potential for detecting endogenous signals from the phosphosites in the panel that are typically at low abundance. Endogenous versions of 89–125 peptides were detected in each individual cell line with a total of 143/250 (57%) detected across all 10 cell lines. Detection of endogenous phosphopeptides reflected the genetic context of the cell lines (Fig EV2C). For example, phosphosites/peptides derived from ALK and FGFR were only detected in lung adenocarcinoma (LUAD) cell line H3122 (driven by *ALK* fusion) and bladder carcinoma cell line RT112 (driven by *FGFR3* fusion), respectively, while in another LUAD cell line PC9, which harbors an activating EGFR mutation (exon 19 deletion) higher levels of EGFR phosphosites were detected.

### Application of the assay to evaluate effects of drug treatment in cancer cell lines

**Figure EV3:**
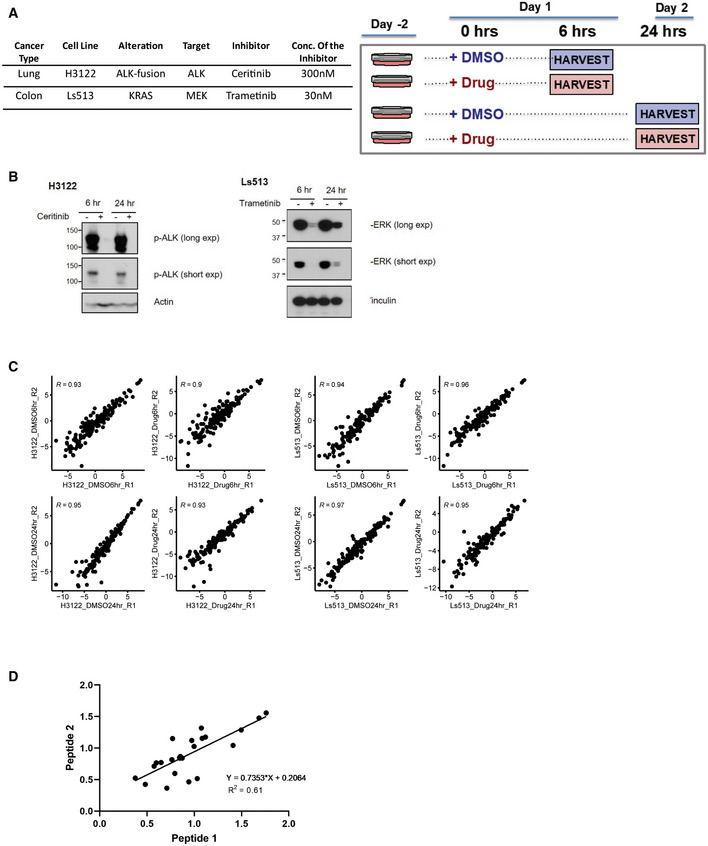
Application of the assay to cancer cell line perturbagen experiments AExperimental design and details for drug treatment studies in H3122 and Ls513 cell lines. Table contains details about the cell lines as well as the inhibitor and concentration of it used. Cells were treated either with the inhibitor or DMSO for 6 and 24 h. Two process replicates were collected for each sample.BInhibition of pALK and pERK signaling in established human cell lines. Immunoblot analyses of cultured H3122 lung adenocarcinoma cells (on the left) treated with ALK inhibitor Ceritinib (+) or DMSO (−) for 6 and 24 h. Antibodies recognizing the phosphorylated 1507‐Tyrosine site of the ALK protein and the Actin protein (loading control) were used. Immunoblot analyses of cultured Ls513 colorectal carcinoma cells (on the right) treated with KRAS inhibitor Trametinib (+) or DMSO (−) for 6 and 24 h. Antibodies recognizing the phosphorylated Thr 185/Tyr 18 sites of the ERK1/ERK2 proteins and the vinculin protein (loading control) were used.CScatter plot of two process replicates of Log_2_ light/heavy PAR of each sample group. Shown on each plot is the Pearson correlation coefficient.DScatter plot of light/heavy peak area ratio of peptide 1 and peptide 2 measuring the same site for 12 of the sites measured in H3122 and Ls513 perturbagen experiments. X‐axis and y‐axis represent light/heavy peak area ratios. Shown on the graph is Pearson correlation coefficient.

To investigate the utility of the SigPath assay to detect and quantify acute perturbations in cell lines, we next treated LUAD H3122 cells with the ALK inhibitor ceritinib (Friboulet *et al*, 2014; Shaw *et al*, 2014) and KRAS mutant Ls513 colorectal cancer cells (CRC) with the MEK inhibitor trametinib (Falchook *et al*, 2012; Flaherty *et al*, 2012; Fig EV3A and B). Cells were treated with the respective inhibitors or DMSO for 6 and 24 h, and two process replicates per condition were analyzed using the SigPath assay as described above. A total of 162 and 155 endogenous phosphopeptides were detected in H3122 (LUAD) and Ls513 (CRC) cell lines, respectively (Dataset EV4). Excellent reproducibility was achieved for replicates, with Pearson correlation > 0.9 for all samples in both cell lines (Fig EV3C). Furthermore, using both LUAD and CRC cell line perturbagen data, we investigated correlation of site quantification as measured by 2 different peptides for the subset of 12 sites for which such data was available. The measured levels of the sites differed by a maximum of 30% from peptide 1 to peptide 2, while the Pearson correlation of the drug/DMSO ratio was 0.6 (Fig EV3D).

**Figure 2:**
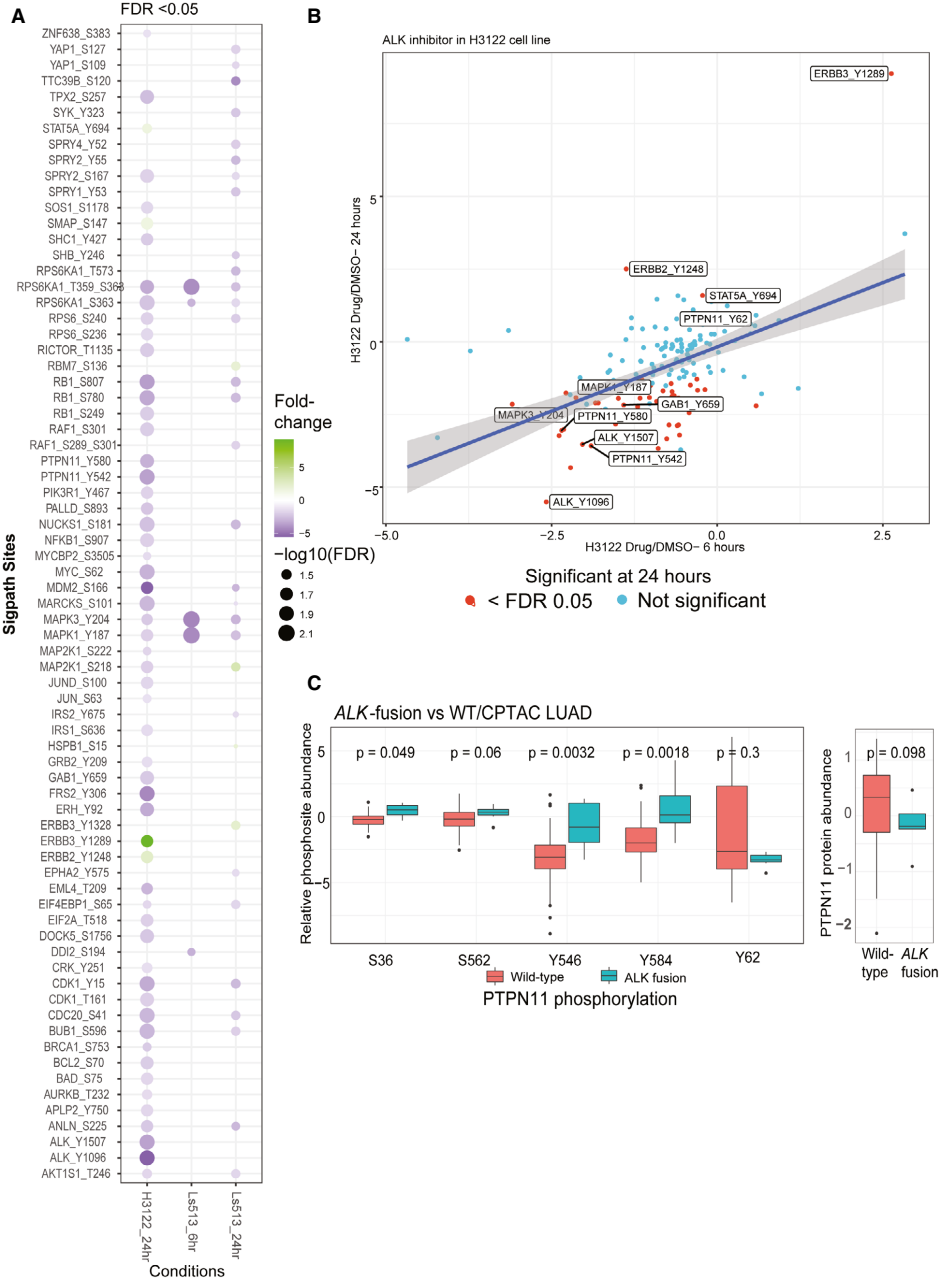
SigPath analyses of cancer cell line perturbation experiments ASummary of all significantly regulated phosphosites observed in H3122 and Ls513 cell lines. The log_2_‐transformed light/heavy peak area ratios for two replicates per time point and treatment were used to compare drug treatment to DMSO. SigPath phosphopeptides differentially regulated upon treatment in each of the conditions in a moderated two‐sample *t*‐test (adj. *P*‐value < 0.05) are shown as circles. H3122 6‐h experiment did not yield any significant regulation and hence not shown on the figure. The color indicates fold change relative to DMSO, and the size of the circle indicates log_10_ (FDR).BScatter plot showing fold change of SigPath sites relative to DMSO for H3122 cell line treated with ALK inhibitor. X‐axis and Y‐axis show 6‐h and 24‐h time points, respectively. The red dots indicate sites with FDR < 0.05. Highlighted are a subset of key differentially regulated phosphosites.CBox plot showing relative abundance of detected PTPN11 phosphosites and PTPN11 protein in the CPTAC LUAD tumors with and without *ALK* fusion (Gillette *et al*, 2020). The box represents interquartile range (IQR) with the lower, central, and the upper bands representing 25^th^ percentile (Q1), median, and 75^th^ percentile (Q3), respectively. The lower and upper whiskers represent Q1‐1.5*IQR, and the upper whisker shows Q3+1.5IQR. The data summarized represents patients wild type (*n* = 103) and mutant (*n* = 7). PTPN11 pY546 and pY584 sites showed the most significant upregulation (*P*‐value < 0.01, Wilcoxon test) in tumors with *ALK*‐fusion.

Ceritinib treatment of H3122 cells resulted in significant regulation of 57 phosphosites, 93% of which were downregulated after 24 h of drug treatment (Fig 2A). Consistent with ALK inhibition, pY sites on ALK (pY1096, pY1507) showed dramatic downregulation (Fig 2B). We also observed downregulation of pathway members downstream of ALK, including in PI3K/AKT and ERK/MAPK pathways as described previously (Miyawaki *et al*, 2017). Interestingly, we also observed differential regulation of PTPN11 phosphosites upon ALK inhibition, with two C‐terminal sites (pY542 and pY580) showing significant reduction at 24 h. ALK activation has been shown to increase PTPN11 phosphorylation at Y542 and Y580 in a neuroblastoma cell line (Sattu *et al*, 2013). Deep‐scale discovery phosphoproteomic analysis of *ALK* fusion‐driven patient‐derived lung adenocarcinoma tumors (Gillette *et al*, 2020) also displayed elevated phosphorylation of PTPN11 Y542 and Y580 (Fig 2C; note that these correspond to Y546 and 584 in the alternative splice isoform of PTPN11), both of which have been implicated in its activation (Bennett *et al*, 1994; Lu *et al*, 2001). PTPN11 regulates cell survival and proliferation (Matozaki *et al*, 2009), and its inhibition suppresses tumorigenesis (Schneeberger *et al*, 2015). ALK resistance is an inevitable consequence of targeted therapy (Rothenstein & Chooback, 2018); in resistant ALK‐driven non‐small‐cell lung cancers, PTPN11 inhibition restores sensitivity to ALK inhibitor therapy (Dardaei *et al*, 2018). Notably, we also observe downregulation of Gab1 phosphorylation at Y659 (Fig 2B), which is required for Gab1‐PTPN11 binding and activation of downstream ERK/MAPK signaling initiated by PTPN11 (Cunnick *et al*, 2001). Our current data showing that ALK inhibition leads to significant downregulation of phosphosites on both the C‐terminal of PTPN11 and Gab1 fits with these other lines of evidence in indicating a key and therapeutically tractable role of this phosphatase in ALK‐mediated downstream signaling both in cell lines and in human tumors. The highly parallel SigPath readout also shows significantly increased phosphorylation at activating sites on ERBB2 (Y1248) and ERBB3 (Y1289), both shown to be involved in the development of resistance during ALK inhibition (Choi *et al*, 2017) and representing possible bypass tracks that can be targeted in resistant disease (Yamaguchi *et al*, 2014). Collectively, these data suggest that in the context of therapeutic perturbation experiments, SigPath can provide key readouts and guide specific, testable hypotheses about response, mechanisms of resistance, and therapeutic alternatives.

In the trametinib‐treated Ls513 colorectal cells, we observed strong downregulation at both 6 and 24 h of MAPK3 (ERK1) pY204 and MAPK1 (ERK2) pY187, both downstream of MEK and consistent with MEK inhibition (Fig 2A). Certain drug treatment effects were exclusively observed at either 6 or 24 h. For example, downregulation of RPS6K1 (pT359 and pS363), which is downstream of ERK, was observed at 6 but not 24 h after Trametinib treatment. Conversely, downregulation of RB1 (pY780 and pS807) and CDK1 (pY15) phosphorylation was significant only after 24 h, suggesting the long‐term impact of MEK inhibition on cell cycle progression via CDK1 and also likely via CDK4/6 inhibition upstream of RB1 phosphorylation (Otto & Sicinski, 2017).

### Application of the assay to breast cancer xenograft tissue samples

The ability to robustly quantify protein phosphorylation events in tumor samples remains limited. Hence, we sought to test the performance of the SigPath assays in tumor cells undergoing *in vivo* treatment. Previously, we carried out a deep proteome and phosphoproteome study of six patient‐derived xenograft (PDX) models of triple‐negative breast cancer (WHIM (Washington University human in mouse) 2, 4, 6, 12, 21, 30) each carrying unique mutations in the PI3K pathway (Mundt *et al*, 2018). These PDX models were selected based on a range of sensitivity to the PI3K inhibitor buparlisib with WHIM4 being the most sensitive and WHIM12 the most resistant. After buparlisib treatment, a differential effect was observed in the phosphoproteome, with the downregulation of phosphosites involved in PI3K signaling seen more in the sensitive than the resistant model. However, due to limits on sensitivity and the stochastic nature of data‐dependent MS‐based proteomics especially for modified peptides, the canonical AKT phosphorylation sites at threonine 308 and serine 473 were neither quantified nor detected.

**Figure 3:**
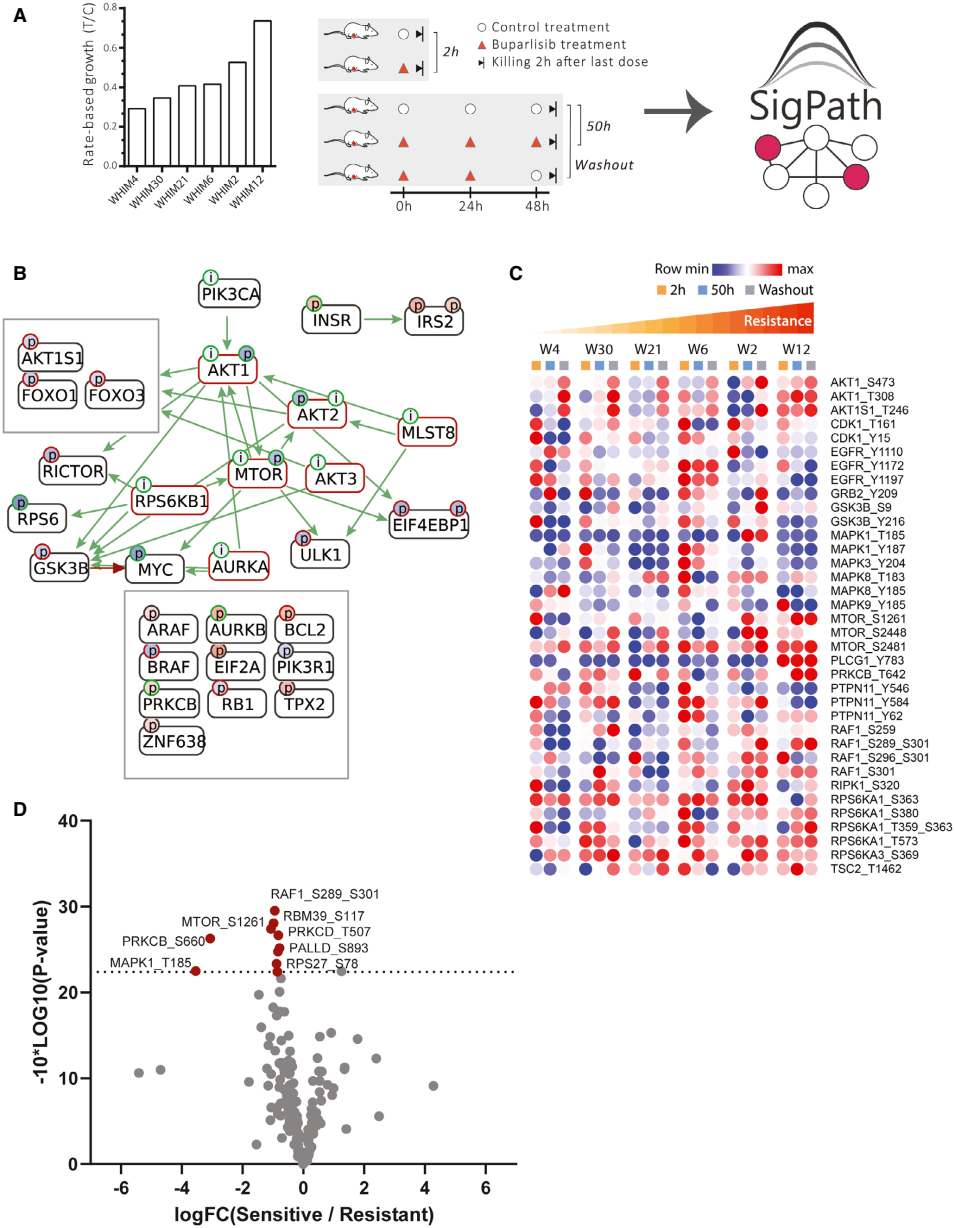
Application of SigPath to understand mechanisms of response and resistance of triple‐negative breast cancer to therapy ASix patient‐derived xenograft models of triple‐negative breast cancer were assessed for their resistance to buparlisib, a PI3K inhibitor, and analyzed for their proteome and phosphoproteome (Mundt *et al*, 2018). The six models ranked after their resistance, from most sensitive to the left (WHIM4), to most resistant to the right (WHIM12). The resistance is calculated as rate‐based growth (treatment over control; T/C). Each PDX model was then treated with buparlisib or vehicle and tumors were collected at hours 2 or 50 (buparlisib/vehicle administered at hours 0, 24, and 48). Each of these six models subjected to five different treatments results in a total of 30 samples that were analyzed with the SigPath assay.BCausalPath (www.causalpath.org) analysis of 2‐h drug/vehicle treatment data. Log_2_ (L/H PAR) for all 6 WHIM models was used for this analysis. Moderated one‐sample *t*‐test was used to analyze 2‐h treatment data. Resulting table was used for the CausalPath analysis. CausalPath network generated by comparing drug‐treated PDX samples to the controls at 2 h. Nodes represent proteins, and the (p) labels on the nodes represent significant differences in site‐specific phosphopeptide measurements. (p) Blue background color indicates a downregulated site, red background color indicates an upregulated site. Green border color around (p) indicates an activatory site, and a red border color indicates an inhibitory site. Green edges represent known site‐specific phosphorylations, and red edges represent dephosphorylations. The label (i) indicates an inhibited protein. In the case of PI3KCA, the label (i) indicates our manually inserted hypothesis of inactivated PIK3CA due to the drug effect. All other (i) labels on the graph are generated automatically by the CausalPath algorithm through statistical evaluation of the changes at the downstream of the protein. CausalPath infers the PI3KCA ‐> AKT1 relation, indicating the downregulated phosphorylation of AKT1 is likely due to inhibition of PIK3CA. Additionally, statistical measurements on the downstream of AKT proteins indicate their inactivation. We observe that this effect extends over downstream targets of AKT such as mTOR.CHeat map of 36 sites from Hallmark’s PI3K_AKT_mTOR pathway and mTOR, including MAPK3_Y204, detected in SigPath assay. Ratio of buparlisib treatment to vehicle for each time point is used. WHIMs are listed in the order of their resistance to buparlisib treatment. The row min, row max color scheme has been applied after the rows have been adjusted to robust Z‐scores (subtracted median and divided by the median absolute deviation; median‐MAD).DVolcano plot comparing resistant versus sensitive models in 50‐h treatment samples. Sensitive (WHIMs 4, 30, 21, and 6) and resistant (WHIMs 2, and 12) are compared in a two‐sample moderated *t*‐test. Log_2_ fold changes are shown on the x‐axis, −10*log_10_ (*P*‐value) derived from the two‐sample moderated *t*‐test are shown on the y‐axis. Red dots indicate the 10 peptides significantly regulated with adj. *P*‐value threshold of < 0.1.

**Figure EV4:**
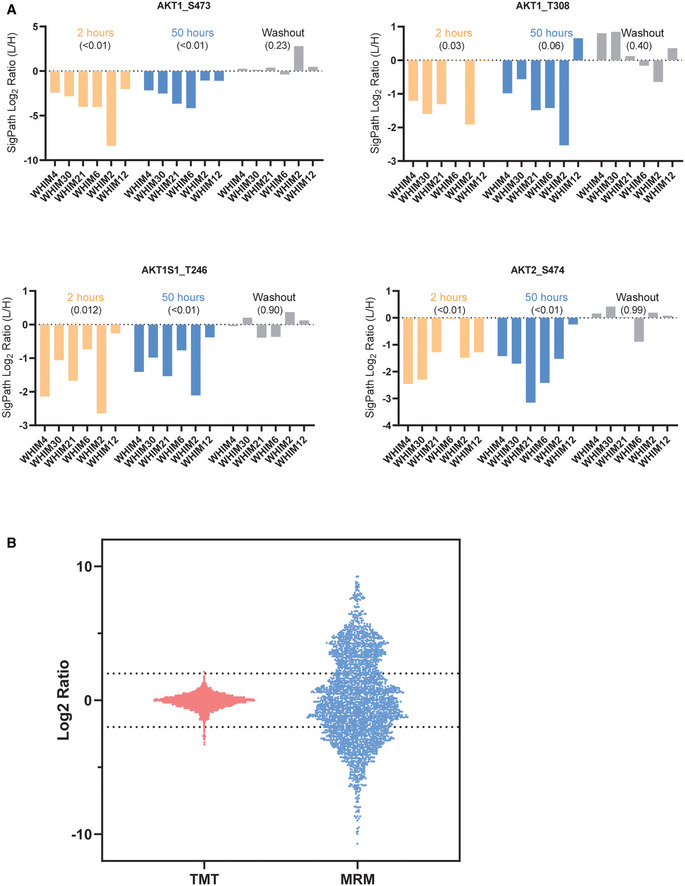
Application of the assay to breast cancer PDX tissue samples ALevels of AKT pS473, AKT pT308, AKT1S1 pT246, and AKT2 pS474 which are all pharmacodynamic markers for PI3K inhibition observed using SigPath. For each graph, and treatment in each graph, the WHIM/PDX models are sorted in the order of their resistance to the drug with least resistance on the left. Across all markers, the most resistant model (WHIM12) is the least affected at 50 h of buparlisib treatment. *P*‐values, in round brackets “()”, are calculated from a one‐sample *t*‐test, compared to a hypothetical mean of 0, using all WHIMS as replicates for a certain treatment.BBar and whisker plot showing the range of ratios obtained for the overlapped 115 peptides in discovery study using TMT and SigPath. For the discovery study, Log_2_ ratio of all the peptides to the pooled reference was used for the plot. For SigPath, Log_2_ ratio of light to heavy peptide was used for the plot.

To assess the SigPath performance in this context, we applied the full SigPath assay, including pY Ab and IMAC enrichments, to tissue samples from the six PDX models (Fig 3A). 185/298 (62%) phosphopeptides in the SigPath assay were detected at the endogenous level in this study (Dataset EV5). The well‐known PI3K inhibition marker for AKT1S1 pT246 was readily detected and quantified, as were the AKT markers pT308 and pS473 that had been missed in the discovery experiment. These sites were readily downregulated after buparlisib treatment, consistent with their status as pharmacodynamic markers of PI3K inhibition. Inhibition of these phosphorylation sites is dependent on the continuous administration of the drug, as evident from the upward trend of the sites following drug washout (Fig EV4A). Interestingly, the most resistant PDX model, which harbors a PIK3CA mutation, is the model that often shows the least effect of PI3K inhibition on either of these sites. CausalPath analysis (Babur *et al*, 2021) applied to the buparlisib/vehicle treatment dataset shows strong inhibition of several members of the PI3K‐AKT‐mTOR pathway after 2‐h buparlisib treatment (Fig 3B). Residual inhibition of the PI3K‐AKT‐mTOR pathway is still visible after 50‐h treatment, but is less pronounced (see CausalPath analysis in Data availability section). These findings substantially recapitulate those from the discovery data (Mundt *et al*, 2018). Interactive exploration of pathway connectivities in this and other datasets described below is available through Data availability section.

To further investigate PI3K‐AKT‐mTOR signaling in the context of buparlisib treatment, we looked at the subset of the SigPath data that include all proteins in Hallmark’s PI3K_AKT_mTOR_signaling pathway and at mTOR itself (Fig 3C). Of the 48 phosphosites represented in the SigPath assay, 36 were readily quantified across the 6 models and treatment conditions. The ratio of buparlisib to vehicle treatment for all models listed in the order of their sensitivity to the treatment is shown in Fig 3C. The resistance of the PDX models to buparlisib does not seem to be mediated at the level of AKT phosphorylation, which suggests that the resistance arises from cross‐talk with other pathways and signaling hubs. To identify response markers, we applied a two‐sample moderated *t*‐test to compare the two most resistant PDX models (WHIM12 and WHIM2) with the other four models (Fig 3D). Among the highly upregulated sites in the resistant models were pS289 and pS301 of RAF1, which is regulated by MAPK3 (ERK1) (Balan *et al*, 2006). The original PDX study (Mundt *et al*, 2018) showed that some of the resistance in the most resistant model (WHIM12) was mediated by MAPK3 activation. Our observations using the SigPath assay strengthen the hypothesis that RAF1 is involved in resistance to buparlisib.

More global comparison of the 146 peptides quantified in both the original Tandem Mass Tag (TMT)‐based and SigPath analyses highlighted the effect of ratio compression in TMT datasets and illustrated how targeted MS overcomes this issue (Fig EV4B).

### Application of the assay to tumor tissue from medulloblastoma patients

To test SigPath in primary human tumor samples, we applied the IMAC portion of the assay (Dataset EV1) to brain tissue specimens from 39/40 medulloblastoma patients representing all established subgroups (WNT, SHH, Gr3, and Gr4), previously analyzed by deepscale proteomics, phosphoproteomics (including IMAC and pY enrichment), and acetylproteomics (Archer *et al*, 2018; Fig EV4A). The pY subset of SigPath was not used due to limited sample availability. The original study showed that tumors with similar RNA expression varied extensively at the post‐transcriptional and post‐translational levels, while proteome profiling revealed subgroups within the SHH and Gr3 groups, providing additional prognostic information and hinting at previously undescribed signaling pathways amenable to SigPath analysis.

**Figure EV5:**
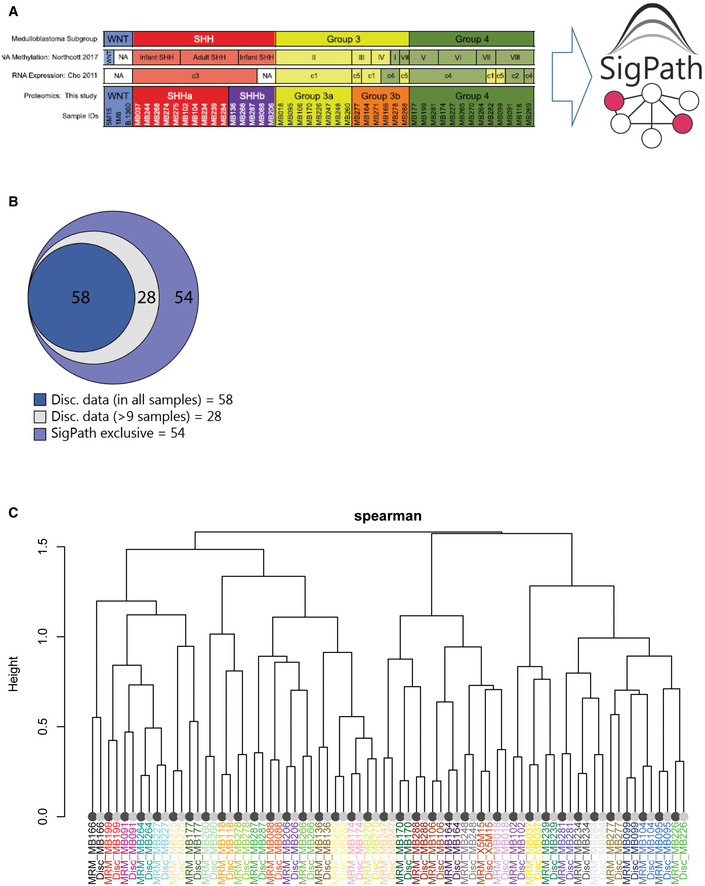
Application of the IMAC subset of the SigPath assay to human tissue samples from medulloblastoma patients AList of samples used for this experiment and their classification to SHH, GR3, and GR4 as well as subgroups within SHH and GR3 (Archer *et al*, 2018). Only IMAC subset of the assay has been applied to these samples.BVenn diagram showing the overlap of the 140 sites detected in SigPath assay with the discovery data. 86 peptides were detected in both datasets, 58 of these were detected in the discovery dataset in all samples (dark blue) while another 28 detected in at least 9 samples (light blue) of the discovery dataset. 54 sites (39%) were unique to SigPath assay (violet).CDendrogram illustrating the clustering of MRM (dark leaves) and discovery data (light leaves) of 86 phosphosites detected in both assays. Colors of sample identifiers are coordinated by patients. Log_2_ TMT ratio of each sample to the pooled reference was used for the discovery data after median‐MAD normalization. Log_2_ light/heavy ratios of SigPath data were used after normalizing each peptide by the median log_2_ light/heavy ratio across all samples. The dendrogram was derived from complete‐linkage hierarchical clustering using 1‐Spearman correlation as the distance metric. All sample pairs (MRM/discovery) cluster adjacent to each other.

A total of 140 of the phosphopeptides targeted in the SigPath IMAC assay (about 60%) were detected across the 39 samples analyzed (Dataset EV6). We looked at the overlap of the 140 phosphosites detected in the SigPath assay with those from the discovery data (Archer *et al*, 2018) and found that 58/140 peptides were detected in all samples in the discovery dataset, while another 28 were detected in at least nine samples of the discovery dataset (Fig EV5B). Correlation analysis of discovery and SigPath results for the 86 sites detected in both demonstrated a high level of agreement between phosphosite abundances as measured by both platforms (Fig EV5C).

**Figure 4:**
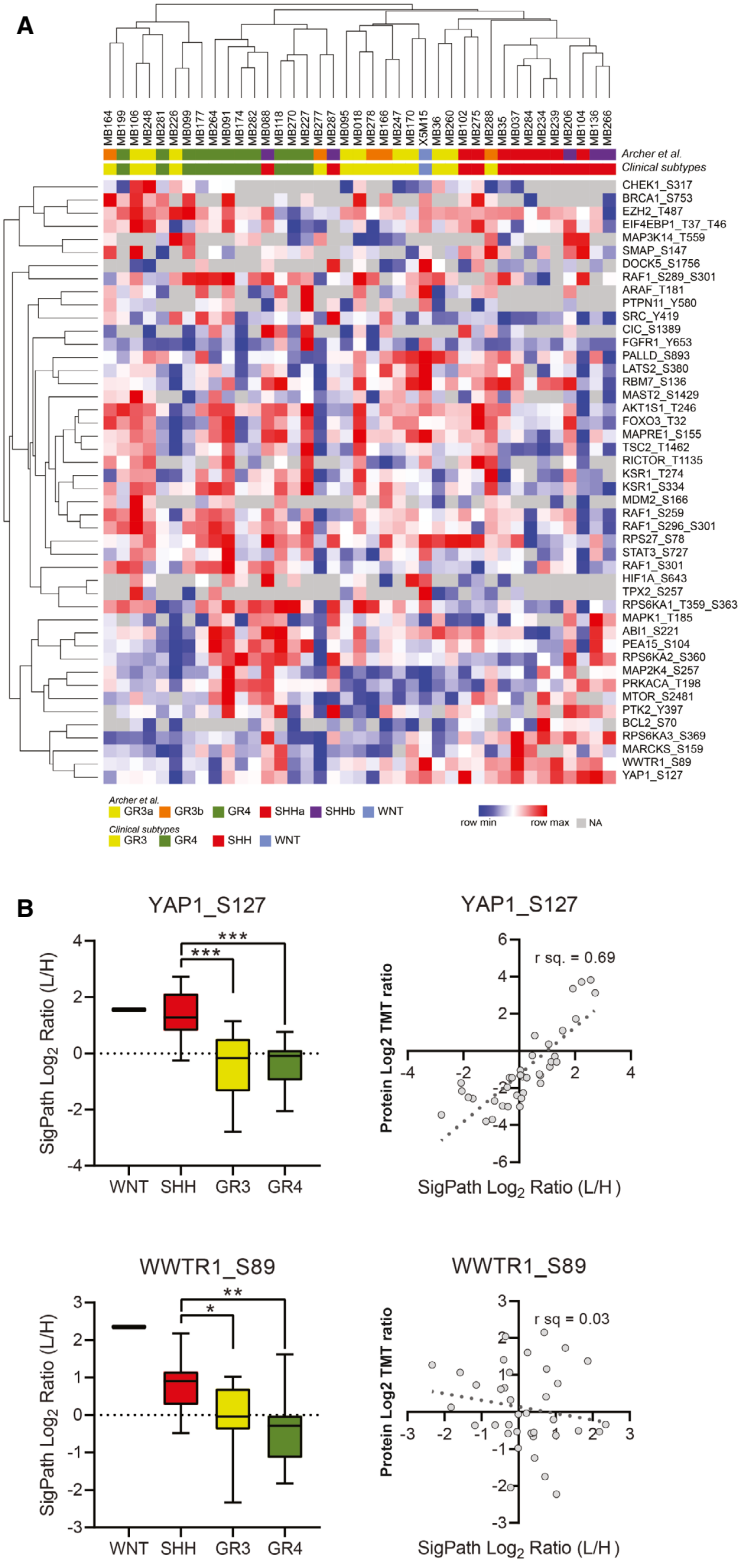
Application of the IMAC subset of SigPath to cancer tissue samples from 39 medulloblastoma patients These patients represent all the known clinical subtypes; WNT (*n* = 1), SHH (*n* = 13), group 3 (GR3; *n* = 13), and group 4 (GR4; *n* = 12).
AA heat map of 39 samples with medulloblastoma showing 46 phosphosites uniquely detected in the SigPath assay. Samples are clustered by their original clinical subtypes as well as by new classification in ref. Archer *et al* (2018) where discovery analyses split subgroup 3 into 3b and 3a, and subgroup SHH into SHHa and SHHb. The heat map was generated using Morpheus online tool, the data are median‐MAD normalized, and colors are relative across rows, from row min to row max.BBox plots comparing all the data for YAP1 pS127 and WWTR1 pS89 for all the samples in different groups of medulloblastoma (SHH, GR3, GR4, and WNT). One‐way ANOVA with an *ad hoc* Tukey’s test (with adj. *P*‐values for multiple comparisons) was applied for the comparison. The box represents interquartile range (IQR) with the lower, central, and upper bands representing 25^th^ percentile (Q1), median, and 75^th^ percentile (Q3), respectively. The whiskers extend from 5 to 95 percentile of the data. Scatter plots comparing TMT protein‐level Log_2_ ratios for YAP1 and WWTR1 to SigPath Log_2_ light to heavy ratios for YAP1 pS127 and TAZ pS89, respectively. Pearson correlation coefficient is shown on the plots.

Fifty‐four phosphosites were uniquely detected and quantified with the SigPath assay (Fig EV5B). The behavior of 46 out of the 54 sites quantified in at least 50 percent of the patient samples is illustrated in Fig 4A. While verification is required, some of the unique sites could constitute novel markers for medulloblastoma subtypes. For example, the pS127 phosphosite of YAP1 is upregulated in the sonic‐hedgehog subtype as compared to both groups 3 and 4 in a one‐way ANOVA test (Fig 4B). Yap1 protein is amplified and upregulated in hedgehog‐associated medulloblastomas (Fernandez *et al*, 2009), while the quantified YAP1 pS127 site indicates inactivation of the protein in this subtype (Artinian *et al*, 2015). YAP1 and WWTR1 have both been shown to be regulated by, and regulators of, the Hippo pathway, specifically phosphorylated at serine 127 and serine 89, respectively, by the LATS tumor suppressor (Totaro *et al*, 2018). LATS1 and LATS2 activation is substantiated by CausalPath analysis in the sonic‐hedgehog subtype, compared to group 3 (see Data availability section for CausalPath analysis link for SHH over GR3 comparison). Phosphorylation of YAP1 at S127, and WWTR1 at S89 results in 14‐3‐3 binding and cytoplasmic retention (Kanai *et al*, 2000; Zhao *et al*, 2007), limiting its ability to co‐activate TEAD transcription factors and inhibiting proliferation (Vassilev *et al*, 2001; Zhao *et al*, 2008; Kofler *et al*, 2018). CausalPath analysis (see Data availability section) also indicated activation of YES1, an upstream effector of YAP1 (Hamanaka *et al*, 2019), in the sonic‐hedgehog subtype compared with group 3. To investigate whether the phosphorylation status of each protein and site could be attributed to protein‐level differences, we extracted protein ratios from the proteomic discovery study for all patient samples and performed correlation analysis with the SigPath assay ratios. High correlation (*R* ^2^ = 0.69) indicates that in SigPath assay YAP1 pS127 is acting as a proxy for the protein‐level difference, while by contrast the pS89 site of WWTR1 seen upregulated in sonic hedgehog versus group 4 is not due to protein‐level difference, as correlation of this phosphosite to the protein is very low (*R* ^2^ = 0.03) (Fig 4B). High expression of the transcriptional coactivator WWTR1 has been shown to be associated with a worse prognosis and affects cell proliferation in patients with medulloblastoma, regardless of subtype (Wang *et al*, 2019); however, phosphorylation of the S89 site leads to an inhibition of carcinogenesis and cell growth (Cordenonsi *et al*, 2011; Zhang *et al*, 2015). Furthermore, CausalPath analysis indicated activation of HCK and YES1 in the sonic‐hedgehog subtype, compared to group 3. The HCK protein product is a member of the Src family of tyrosine kinases. A positive feedback loop between GLI1 and the tyrosine kinase HCK has been shown to amplify sonic‐hedgehog signaling in medulloblastoma (Shi *et al*, 2015). These new phospho‐level findings may be of significant interest to the medulloblastoma community.

## Discussion

Targeted MS assays have largely been developed for purposes of biomarker verification, but are increasingly being used to validate findings in biological and preclinical studies (as reviewed in (Rifai *et al*, 2006; Parker & Borchers, 2014). The highly multiplexed, targeted quantitative assay we developed and applied here was designed to measure phosphosites in nodes of biological pathways known to be modulated via phosphorylation, including the RAS, MAPK, PI3K, PKC, SRC, and JAK signaling pathways, transcription factor circuit nodes including FOXO, STAT, NFKB, TGFB, and Wnt pathways, as well as protein phosphosites useful as readouts of DNA damage, cell cycle arrest, apoptosis, spindle checkpoint activation, hypoxia, autophagy, cell stress, and epithelial‐to‐mesenchymal transition. It is important to note that the coverage of these pathways by the current SigPath assay is variable and incomplete, but the sites measured provide some preliminary information that can be used as starting points for further exploration. Furthermore, the assay can be expanded to include additional sites of interest. Through a range of applications in cell lines, preclinical models, and clinical samples, we demonstrated the utility of the assay to not just verify prior findings, but to detect and quantify a large number of differentially regulated phosphosites newly associated with drug perturbation and disease subgroups. For example, in an *ALK* fusion cell line with the treatment of Ceritinib, we observed differential phosphorylation of N‐ and C‐terminal phosphosites from PTPN11 phosphatase consistent with prior observations in primary LUAD tissue samples (Gillette *et al*, 2020). These results suggest that SigPath, and targeted MS assays in general, should be more routinely used in the development and optimization of therapeutics. Our results also highlight the potential of SigPath to monitor phosphoproteomic signaling events and to nominate mechanistic hypotheses regarding oncogenesis, response, and resistance to therapy in disease models and human tumors.

The ca. 300‐plex SigPath assay was designed to flexibly allow measurement of phosphosites having phosphotyrosine alone, chiefly phosphoserine‐ and phosphothreonine sites, or a mix of all three. Any proteomic lab skilled in phosphopeptide enrichment can implement the assay, and phosphopeptide sample enrichment is readily automated (Abelin *et al*, 2016). The SigPath assay is readily extendable to measure other phosphosites by synthesis of the new phosphopeptides in heavy‐labeled form and repeating the QC processes described with a focus on the new sites. Three hundred targets does not represent the maximal level of multiplexing that can be achieved. Using modern MS instrumentation and techniques such as internal standard‐triggered parallel reaction monitoring (Gallien *et al*, 2015), assay panel sizes can be increased to 500 targets or more. In contrast, assays employing anti‐peptide antibodies (Kuhn *et al*, 2009; Whiteaker *et al*, 2011, 2014; Keshishian *et al*, 2015; Sperling *et al*, 2019) take a long time to develop and qualify, are very costly even for small numbers of targets, have yet to be multiplexed to the level demonstrated here, and are currently available in only a few expert labs, limiting their utility for the biology community. A limitation of SigPath as configured is that it profiles and quantifies changes in phosphorylation levels only, and therefore cannot determine whether the observed change in phosphopeptide level was due to altered protein expression or a site‐specific post‐translational effect. Assessing if the change in phosphorylation observed is more likely due to protein‐level change vs. site‐specific phosphorylation change could, in principle, be accomplished by adding heavy non‐phosphorylated peptide standards to the flow‐through from the IMAC enrichment and measuring these non‐phosphorylated peptides in a separate and parallel assay. The success of this method is entirely dependent on the ability to detect and quantify the unmodified peptide in the complex digest using a single shot approach.

While Western‐competent antibodies exist for many of the targets in SigPath, they are generally deployed in a highly selective and individual manner and are non‐quantitative, resulting in large swaths of biology remaining opaque to the investigator. Multiplexed Western and ELISA panels are commercially available, but typically measure only 10–20 analytes per well in order to avoid cross‐reactivity and maintain sensitivity (e.g., Quanterix, MSD, Myriad, Abcam, AbcamReview). Large protein assay panels employing two different antibodies for each protein target with detection based on proximity extension are also now commercially available from Olink (Olink). However, these assay panels primarily measure proteins, not phosphosites.

The depth of detection in any targeted MS approach is governed by the abundance of the target peptide in the sample being analyzed, the efficiency of the enrichment process and the amount of input peptide that was enriched. When IMAC phosphopeptide enrichment alone is used, the efficiency is ca. 95%; that is, less than 5% the peptides observed after enrichment do not contain a phosphorylated amino acid. This is important, as non‐phosphorylated peptides are generally present at much higher abundance than the phosphopeptides and so can interfere with detection of phosphopeptides. In the three studies presented here, 500–1,000 μg of peptides was used for the IMAC portion of the assay; however as little as 50 μg can be used, with consequent loss of detection of a subset of the least abundant phosphopeptides. Due to the lower abundance of phosphotyrosine‐containing peptides, antibody‐based phosphotyrosine peptide enrichment typically requires a larger sample input, often in the range of 1–5 mg, to get high coverage of the sites in the SigPath assay.

Once targeted MS‐based assays achieve the large numbers presented here in SigPath, they can also be viewed as a different, complementary way to do discovery, with a narrower spectrum but more consistent measurements and vastly greater throughput. For example, the bona fide PI3K inhibition marker AKT S473 was missed completely in the PDX discovery dataset, while the SigPath assay consistently quantified this site. Sample preparation for SigPath is simple, requiring only digestion, phosphopeptide capture and analysis of the captured peptides together with spiked heavy peptide standards. While sample processing was done manually in the present study, throughput for SigPath can be greatly increased using automated digestion and IMAC enrichment on liquid handling robots as we have previously demonstrated (Abelin *et al*, 2016). Antibody‐based capture of pY peptides will also become much faster and more reproducible once these antibodies are conjugated to magnetic beads for processing on systems like the Kingfisher as we have done in the case of KGG‐peptide capture for ubiquitylation profiling (preprint: Rivera *et al*, 2021). The assay as presented here requires a total of 5 h of on‐instrument time for the analysis of both pY Ab‐ and IMAC‐captured samples. This time can be shortened with faster MS instrumentation, use of shorter gradients or by mixing pY and IMAC captures and analyzing these together in a single LC‐MRM/MS run. The use of FAIMS for post‐translationally modified peptides would also provide another level of separation and potentially increase sensitivity (Udeshi *et al*, 2020; Popow *et al*, 2021). Quantification of phosphopeptides using SigPath is far more precise than label‐free discovery experiments or those using isobaric chemical labels like TMT, as targeted MS methods do not suffer from the ratio compression challenges of the latter. While the breadth of coverage in SigPath is much smaller than discovery phosphoproteomic analyses (> 300 phosphopeptides compared to > 30,000 phosphopeptides per sample), the quantitative precision and repeatability for targets measured by the assay confer their own advantages, as shown here in the replicates of the ALK inhibitor study and in other phosphopeptide assays we have uploaded to the CPTAC assay portal. Since SigPath detects and measures the spiked heavy peptide forms of all of the phosphopeptides targeted, determining the presence and level of the endogenous form by ratioing the intensities of the endogenous and spiked peptide‐specific fragment ions, it, like other targeted MS assays, is far less susceptible to false positives, meaning that if the target was not detected, it was below detection limits rather than due to stochastic sampling. Therefore, we view SigPath as an impactful resource for the cancer research community, suitable for discovery, as a verification assay for targets of biological import, and for preclinical studies in human cancers and other diseases.

## Materials and Methods

### Reagents and Tools table

**Table:** 

Reagent or Resource	Source	Identifier
**Antibodies**		
PTMScan‐pY 1000 Rabbit mAB kit	Cell Signaling Technology	Catalog: 8803A
**Biological Samples**		
Primary tumor samples	See Experimental Model and Subject Details	N/A
**Chemicals and Reagents**		
Synthetic [C13,N15] labeled peptides	New England Peptide	N/A
HPLC‐grade water	J.T. Baker	Catalog: 4218‐03
Urea	Sigma	Catalog: U0631
Sodium chloride	Sigma	Catalog: 71376
1M Tris, pH 8.0	Invitrogen	Catalog: AM9855G
Ethylenediaminetetraacetic acid	Sigma	Catalog: E7889
Aprotinin	Sigma	Catalog: A6103
Leupeptin	Roche	Catalog: 11017101001
Phenylmethylsulfonyl fluoride	Sigma	Catalog: 78830
Sodium fluoride	Sigma	Catalog: S7920
Phosphatase inhibitor cocktail 2	Sigma	Catalog: P5726
Phosphatase inhibitor cocktail 3	Sigma	Catalog: P0044
Dithiothreitol, No‐Weigh Format	Fisher Scientific	Catalog: 20291
Iodoacetamide	Sigma	Catalog: A3221
Lysyl endopeptidase	Wako Chemicals	Catalog: 129‐02541
Sequencing‐grade modified trypsin	Promega	Catalog: V511X
Formic acid	Sigma	Catalog: F0507
Acetonitrile	Honeywell	Catalog: 34967
Trifluoroacetic acid	Sigma	Catalog: 302031
Methanol	Honeywell	Catalog: 34966
Ni‐NTA agarose beads	Qiagen	Catalog: 30410
Iron (III) chloride	Sigma	Catalog: 451649
Potassium phosphate, monobasic	Sigma	Catalog: P0662
Potassium phosphate, dibasic	Sigma	Catalog: P3786
MOPS	Sigma	Catalog: M5162
Sodium hydroxide	VWR	Catalog: BDH7225
Phosphate‐buffered saline	Fisher Scientific	Catalog: 10010023
**Equipment**		
Reversed‐phase tC18 Sep‐Pak, 1cc 100 mg	Waters	Catalog: WAT023590
Reversed‐phase tC18 Sep‐Pak, 3cc 200 mg	Waters	Catalog: WAT054945
Solid‐phase C18 disk, for stagetips	Empore 3M	Catalog: 66883‐U
Stage‐tip needle	Cadence	Catalog: 7928
Stage‐tip puncher, PEEK tubing	IDEX Health & Science	Catalog: 1581
PicoFrit LC‐MS column	New Objective	Catalog: PF360‐75‐10‐N‐5
ReproSil‐Pur, 120 Å, C18‐AQ, 1.9‐μm resin	Dr. Maisch	Catalog: r119.aq
Nanospray column heater	Phoenix S&T	Catalog: PST‐CH‐20U
Column heater controller	Phoenix S&T	Catalog: PST‐CHC
300 μl LC‐MS autosampler vial and cap	Waters	Catalog: 186002639
96‐well microplate for BCA	Greiner	Catalog: 655101
Microplate foil cover	Corning	Catalog: PCR‐AS‐200
Vacuum centrifuge	Thermo Fisher	Catalog: SPD121P‐115
Centrifuge	Eppendorf	Catalog: 5427 R
Benchtop mini centrifuge	Corning	Catalog: 6765
Benchtop vortex	Scientific Industries	Catalog: SI‐0236
Incubating shaker	VWR	Catalog: 12620‐942
15‐ml centrifuge tube	Corning	Catalog: 352097
50‐ml centrifuge tube	Corning	Catalog: 352070
1.5‐ml microtube w/o cap	Sarstedt	Catalog: 72.607
2.0‐ml microtube w/o cap	Sarstedt	Catalog: 72.608
**Instrumentation**		
Microplate Reader	Molecular Devices	Catalog: M2
Online LC for LC‐MS	Thermo Fisher	Catalog: LC140
Q Exactive Plus Mass Spectrometer	Thermo Fisher	Catalog: IQLAAEGA APFALGMBDK
TSQ Quantiva Triple Quadrupole Mass Spectrometer	Thermo Fisher	Catalog: IQLAAEGAAXFAOUMZZZ
**Critical Commercial Assays**		
BCA Protein Assay Kit	Thermo Fisher	Catalog: 23225
**Software and Algorithms**		
Software	Source (i.e., PMID or lab)	Identifier (i.e., links)
Spectrum Mill software package v7.0	Broad Institute, Cambridge, MA	https://proteomics.broadinstitute.org/
Protigy	Broad Institute, Proteomics Platform	https://github.com/broadinstitute/protigy
Skyline	University of Washington, Seattle, WA	https://skyline.ms/project/home/software/Skyline/begin.view ?
Panorama	University of Washington, Seattle, WA	https://panoramaweb.org/home/project‐begin.view ?
CausalPath	Computer Science Department, University of Massachusetts Boston, Boston, MA	www.causalpath.org

### Methods and Protocols

#### Human subjects

Primary medulloblastoma patient samples were collected as described in ref. Archer *et al* (2018). Patient samples, including FFPE slides, were obtained with informed consent according to the International Cancer Genome Consortium (ICGC) guidelines as approved by the Ethics Committee of the Medical Faculty at Heidelberg University, and as approved by the institutional review board of contributing center Nikolay Nilovich Burdenko Neurosurgical Institute in Moscow. De‐identified tumor samples of 50 mg were freeze‐fractured using Covaris cryoPREP CP02 at setting ‘‘impact level 4’’, and the pulverized samples were aliquoted for the downstream analysis.

#### Patient‐derived Xenographs

Six triple‐negative breast cancer, patient‐derived xenograft (PDX) models with moderate‐to‐high PI3K pathway activity were selected from the Washington University Human in Mouse (WHIM) PDX collection as described in ref. Mundt *et al* (2018). All human tissues for these experiments were processed in compliance with NIH regulations and institutional guidelines, and approved by the institutional review board at Washington University. All animal procedures were reviewed and approved by the institutional animal care and use committee at Washington University in St. Louis. PDX models are available through the application to the Human and Mouse‐Linked Evaluation of Tumors core at http://digitalcommons.wustl.edu/hamlet/, and additional information can be found in ref. Li *et al* (2013).

#### Peptide selection by nomination and selection from phosphoprofiling studies

For the list of nominated phosphosites, the first step was to convert them into tryptic peptides containing the sites. An *in silico* tryptic digest of the UniProt protein database was generated to identify fully cleaved tryptic peptides containing the nominated phosphosites. Peptides longer than 40 amino acids in the list were dropped at this step of the selection process. Short versions (< 6 amino acids) of fully cleaved tryptic peptides were still considered at this step in case missed cleaved versions with reasonable length were observed in the existing datasets (Fig EV1A).

Next step was to ascertain whether they had been detected by mass spectrometry. For this purpose, we used the large collection of high‐quality phosphopeptide data generated by the proteomics group at the Broad Institute over the past 15 years to identify whether and in which form(s) the tryptic phosphopeptide had been most frequently observed (e.g., full tryptic, incomplete cleavage, etc.). For the search, we utilized in‐house developed R and Perl scripts specifically tailored toward database search results created by Spectrum Mill Software (Broad Institute, Cambridge, MA) or MaxQuant (Cox & Mann, 2008). Only validated peptides at 1% false discovery rate (FDR) were considered in subsequent analysis. Missed cleaved versions of the query peptide were allowed. Peptides identified by this approach were manually analyzed for each peptide to select a version for synthesis. In the cases where more than one version of the peptide was observed (fully cleaved or missed cleaved, singly or multiply phosphorylated), priority was given to the version with the highest frequency of observation in the datasets. In selecting the singly versus the multiply phosphorylated version of a peptide, priority was given to singly phosphorylated version unless doubly phosphorylated version was much more prevalent in existing datasets. Moreover, for MAPK1, MAPK14, MAPK3, MAPK8, MAPK9, RAF1, and RPS6KA1 both singly phosphorylated and doubly phosphorylated forms of the peptides were included (see Dataset EV1). There are no triply phosphorylated peptides in the assay panel.

Seventy percent of sites nominated (234 out of 343) were previously observed as fully tryptic phosphopeptides or in a missed cleaved form in our experimental datasets. Dataset EV1 lists all of the previously observed nominated phosphosites, the phosphoprotein of origin, the dominant tryptic peptide form containing each site, and the enrichment methodology required for detection of the site (i.e., either immobilized metal affinity chromatography (IMAC) or phosphotyrosine (pY) antibody). Sixty‐two phosphopeptides containing 66 of the 343 nominated phosphosites were not observed in our datasets (Datasets EV1 and EV7). Despite the lack of prior observation in discovery data, we included 37 of these phosphopeptides due to their importance in cancer biology and the potential for the targeted MS method to have greater sensitivity for their detection than the discovery methods used. The remaining 29 sites were excluded from consideration (Dataset EV7).

In addition to lack of detection in experimental data, there were a number of other reasons to not advance the assay configuration for some of the nominated phosphosites, including target peptides being too long or too short or having failed synthesis. Nineteen of the nominated sites were located in tryptic peptides that were deemed to be too long (> 40 amino acids) and were eliminated because of anticipated issues with synthesis, chromatography, and/or assay development (Dataset EV7). In addition, a number of nominated sites were located in short tryptic peptides of 6 amino acids or less. To improve likelihood of detection, specificity and chromatographic retention on C18 reverse phase matrix, we instead searched the data for longer, missed cleaved forms of these peptides. Seventeen of the total 25 short peptides were found in longer, missed cleaved peptides (> 6 but < 40 amino acids) and were included for assay development. For example, the nominated site pS380 in serine/threonine–protein kinase LATS2 is present in tryptic peptide form D(pS)LQK. The longer, missed cleaved version of this peptide RD(pS)LQKPGLEAPPR was found in the discovery data and selected for assay configuration (Dataset EV1). Missed cleaved forms for eight additional short phosphopeptides were not detected in the data and so these were removed for further consideration (Dataset EV7).

Seventy‐three phosphosites in the panel were derived from quantitative proteomic discovery studies in our laboratory and were among the most significantly regulated sites in those studies. 17 phosphopeptides were selected from a discovery phosphoprofiling study investigating impact of ischemia in ovarian cancer and breast cancer xenograft tissues (Mertins *et al*, 2014). The remaining experimentally derived sites were included based on the analysis of several discovery experiments done in cancer cell lines treated with specific inhibitors.

During the selection process, the uniqueness of each peptide in the human proteome was also taken into consideration with priority given to peptides unique to one protein. Seven of the peptides in the final assay panel are shared with more than one protein (Dataset EV1). Among these are: pS909 of LATS1 and pS872 of LATS2; pT1079 of LATS1 and pT1041 of LATS2; pT35 of MOB1A and MOB1B; pS907 of NFKB1 and pS276 of RELA; pT198 of PRKACA, PRKACB, and PRKACG; pS621 of RAF1 and pS582 of ARAF: and pY706 of NTRK2 and pY709 of NTRK3.

#### Peptide synthesis

The final list of 352 tryptic phosphopeptides representing 344 phosphosites (17 peptides having more than one site) corresponding to 234 phosphoproteins were synthesized containing single stable isotopically labeled (SIL) amino acid. Most peptides contained [13C, 15N] lysine or arginine at the C‐terminus. Nine peptides representing C‐terminal of the protein were synthesized with either heavy N‐terminal lysine or arginine, or heavy internal leucine and proline. All synthetic peptides were purified by the vendor to greater than 95% purity, quantified by amino acid analysis, and received at micromolar concentrations in a buffer containing 0.1% formic acid/30% acetonitrile. Aliquots of these solutions were diluted to 100 pm/μl for preparation of the peptide mixtures. Peptides are stored at −80⁰C at the Broad Institute and are intended for in‐house use only.

#### Peptide organization

Synthetic heavy‐labeled internal standard peptides were organized in mixtures first by their enrichment methodology (immobilized metal affinity chromatography (IMAC) and/or phosphotyrosine (pY) antibody). Two hundred thirty one peptides containing phosphoserine (pS) or phosphothreonine (pT) were organized in five IMAC mixtures alphabetically by gene name containing 43–50 phosphopeptides each. Seventy‐one pY‐containing peptides were organized in 2 pY mixes alphabetically by gene name, each containing 31 or 40 peptides. Finally, the remaining 50 peptides (predominantly pY) with previous detection information after IMAC as well as pY antibody enrichments were organized in a separate mixture (IMACpY) and used in combination with either IMAC or pY mixtures. Peptides were at 2 pm/μl equimolar concentration in all of the mixtures.

#### Assay configuration

Multiple reaction monitoring (MRM) assay configuration using heavy‐labeled synthetic peptides was done on TSQ Quantiva Triple Quadrupole Mass Spectrometer (Thermo Fisher) coupled with Easy‐nLC 1200 ultra‐high pressure liquid chromatography (UPLC) system (Thermo Fisher) in several batches of 50–100 peptides each. Skyline Targeted Mass Spec Environment was used throughout assay configuration and all data analysis. First, spectral libraries for the peptides were generated on a Q Exactive mass spectrometer. Spectral libraries were uploaded to Skyline, and 5–10 most intense fragment ions (transitions) for each peptide were selected for MRM assay configuration. Transitions containing phosphosite or helping with the assignment of the phosphosite were included in the transition list for assay configuration. Next, collision energies (CE) were optimized for all the transitions and peptides by liquid chromatography–multiple reaction monitoring mass spectrometry (LC‐MRM/MS) on TSQ Quantiva using Skyline’s CE optimization module. For every transition starting with the instrument‐specific calculated CE tested 10 additional CEs (5 below and 5 above the calculated CE) in increments of 2. The list of transitions with varying CE values was exported from Skyline and used for building the MRM method in Xcalibur software. Equimolar mixture of peptides at 50 fm/μl was analyzed by LC‐MRM/MS on Quantiva using this method. Resulting data were analyzed on Skyline which then selected the CE that resulted in the highest peak area for each transition. In the final step of CE optimization MRM data were acquired with optimized CE values for every transition. Using this dataset in Skyline, manually selected the best 3–6 transitions for every peptide giving highest priority to fragment ions of y‐series with mass to charge (m/z) above the precursor, and ions containing phosphosite or helping with the site localization. For the peptides where the options were more limited also included ions of y‐series with m/z below the precursor and b‐series.

While we configured the assay on TSQ Quantiva MS, other triple quadrupole instruments can be used for this assay with further optimization of MS‐specific parameters for each instrument (Kuhn *et al*, 2012; Abbatiello *et al*, 2015).

After the CE optimization compiled 2 LC‐MRM/MS methods, one for peptides enriched by IMAC strategy (231 IMAC and 50 IMACpY mixtures) and the second for peptides enriched by pY antibody strategy (71 pY and 50 IMACpY mixtures).

Liquid chromatography was performed on 75 μm ID PicoFrit columns packed in‐house to a length of 28–30 cm with Reprosil C18‐AQ 1.9 μm beads (Dr Maisch GmbH) with solvent A of 0.1% formic acid (FA) / 3% acetonitrile (ACN) and solvent B of 0.1% FA / 90% ACN at 200 nl/min flow rate. Below are the details of the IMAC and pY LC‐MRM/MS methods:

*IMAC LC‐MRM/MS method*: method duration – 160 min, gradient – 2–6% solvent B in 1 min, 6–30% B in 124 min, 30–60% B in 9 min, 60–90% B in 1 min, followed by a hold at 90% B for 5 min, and subsequent hold at 50% B for 19 min. MS parameters include 3‐sec cycle time, Q1 and Q3 resolution of 0.4 and 0.7, respectively, retention time (RT) scheduling window of 10 min.

*pY LC‐MRM/MS method*: method duration – 120 min, gradient – 2–6% solvent B in 1 min, 6–30% B in 84 min, 30–60% B in 9 min, 60–90% B in 1 min, followed by a hold at 90% B for 5 min, and subsequent hold at 50% B for 19 min. MS parameters include 1.5‐sec cycle time, Q1 and Q3 resolution of 0.4 and 0.7, respectively, RT scheduling window of 10 min.

#### Lysis and digestion of cell line and tissue samples

1. Add lysis buffer (8 M urea, 75 mM NaCl, 50 mM Tris pH 8.0, 1 mM EDTA, 2 μg/ml Aprotinin, 10 μg/ml Leupeptin, 1 mM PMSF, 10 mM NaF, 1:100 phosphatase inhibitor cocktail 2 and 1:100 phosphatase inhibitor cocktail 3) to the cell pellets or cryopulverized tissue samples, vortex lightly, and incubate at 4°C with end‐over‐end rotation for 15 min.
2. Vortex samples for 10 s on the highest setting and allow to incubate at 4°C with end‐over‐end rotation again for 15 more minutes.
3. Centrifuge samples at 20,000 rcf for 10 min at 4°C to pellet insoluble cell debris.
4. Transfer the supernatant to a new 2‐ml Eppendorf tube and quantify by the Pierce BCA Protein Assay Kit.
5. Equalize concentrations of a set of samples in a study and digested together by adding more lysis buffer to samples that are more concentrated to match the sample with the lowest concentration.
6. Reduce samples with 5 mM dithiothreitol (DTT, Pierce, A39255), mixing at 800 rpm for 45 min at room temperature.
7. Alkylate samples using 10 mM iodoacetamide (IAA, Sigma‐Aldrich, 144489) for 45 min in the dark at room temperature.
8. Dilute the samples 1:4 with 50 mM Tris–HCl pH 8.0 and digest with LysC (Wako) at an enzyme to substrate ratio of 1:50 for 2 h at 30°C and shaking at 800 rpm.
9. Add trypsin (Promega) at an enzyme to substrate ratio of 1:50 overnight at 37°C and shaking at 800 rpm.
10. Quench the digestion with 10% formic acid to a final concentration of 1% and pH 3.

#### Peptide cleanup by cartridge desalt

1. Condition 200 mg (3 cc) Sep‐Pak C18 Vac Cartridges (Waters) with 3 ml of acetonitrile followed by 3 ml of 0.1% FA / 50% ACN.
2. Equilibrate them with four 3 ml injections of 0.1% trifluoroacetic acid (TFA).
3. Load the samples onto the cartridges and collect the flow‐through.
4. Wash the samples three times with 3 ml 0.1% trifluoroacetic acid and one time with 3 ml 1% FA.
5. Elute the samples off the cartridges with two injections of 1.5 ml 0.1% FA / 50% ACN into 15‐mL centrifuge tubes.
6. Freeze the samples and dry them down with vacuum centrifugation.
7. Reconstitute the samples in 0.1% FA / 3% ACN and quantify by the Pierce BCA Protein Assay Kit.
8. Make 5 mg aliquots of the samples for pY Ab enrichment, freeze the aliquots, and dry down by vacuum centrifugation.
9. If pY Ab enrichment is skipped, then make 500 μg aliquots for the IMAC enrichment step (see below).

#### Phosphotyrosine enrichment

1. Reconstitute peptide aliquots for pY Ab enrichment with 1.5 ml of IAP buffer (50 mM MOPS/NaOH pH 7.2, 10 mM Na_2_HPO_4_, 50 mM NaCl) and keep on ice throughout the experiment.
2. Add 30 fmol of the pY and IMACpY heavy peptide mixtures into each sample, then vortex, and spin down at 5,000 rcf for 5 min.
3. Wash the pY 1000 Immunoaffinity beads (Cell Signaling Technology) 3 times, each time with 1.5 ml of IAP buffer. Remove the supernatant after each wash.
4. Add reconstituted peptide samples onto the Immunoaffinity beads and mix end over end at 4°C for 1 h.
5. After the hour incubation, spin down the beads at 1,500 rcf for 1 min and collect the supernatant as the pY flow‐through for IMAC enrichment.
6. Wash pY 1000 beads four times with 1.5 ml cold phosphate‐buffered saline (PBS, Thermo Fisher).
7. After washing, resuspend the beads with 50 μl of 0.15% TFA and incubate at room temperature for 5 min.
8. Spin down the beads and transfer the supernatant onto a prewashed and pre‐conditioned stagetip (see below).
9. Repeat TFA incubation one more time for a total of 100 μl of supernatant transferred onto the stagetip. After the second elution, transfer the beads along with the supernatant to the stagetip.

#### Phosphotyrosine enrichment stage‐tip desalt

1. Condition the stagetips, prepared with two Empore C18 (3 M) punches with 100 μl of methanol, and followed by 100 μl of 0.1% FA / 50% ACN. Spin down after each one at 3,100 rcf for 1 min.
2. Equilibrate with 2 injections of 100 μl 0.1% FA.
3. Add the two 50 μl pY captured samples along with the beads to stagetips then spin down.
4. Wash the sample with two injections of 100 μl 0.1% FA and spin down after each.
5. Elute the bound peptides off the stagetips using 50 μl of 0.1% FA / 50% ACN.
6. Transfer the eluates to autosampler vials, freeze, and dry down.
7. Reconstitute in 5 μl of 0.1% FA/3% ACN solution and inject 4 μl for LC‐MRM/MS analysis on TSQ Quantiva using pY LC‐MRM/MS method (see above).

#### Phosphotyrosine flow‐through desalt

1. Condition 100 mg (1 cc) Sep‐Pak C18 Vac Cartridges (Waters, WAT023590) with 1 ml of acetonitrile followed by 1 ml of 0.1% FA / 50% ACN.
2. Equilibrate with four 1 ml injections of 0.1% TFA.
3. Acidify pY Ab enrichment flow‐through samples with 150 μl of 10% FA and load onto the prepared cartridges in two steps of 750 μl.
4. Wash them three times with 1 ml of 0.1% TFA then one time with 1 ml 1% FA.
5. Elute the samples off the cartridges and into 2‐mL Eppendorf tubes with 2 injections of 750 μl 0.1% FA / 50% ACN.
6. Freeze the samples and dry down by vacuum centrifugation.
7. Reconstitute the samples in 0.1% FA / 3% ACN and measure the concentrations using the Pierce BCA Protein Assay Kit (Thermo Fisher).
8. Make aliquots at appropriate amounts for each study (500–1,000 μg) for the immobilized metal affinity chromatography (IMAC) enrichment step. Freeze the aliquots and dry down by vacuum centrifugation.

#### Immobilized metal affinity chromatography (IMAC) phosphopeptide enrichment

1. Remove 1,200 μl slurry, or 600 μl of Ni‐NTA Agarose beads (Qiagen) and transfer to a 1.5‐ml Eppendorf tube.
2. Wash the beads three times by adding 1 ml of water onto the beads, inverting the tube to suspend the beads, then spinning down for 1 min at 1,500 rcf and removing the supernatant. Strip the beads of the nickel by incubating end over end with 1,200 μl of 100 mM ethylenediaminetetraacetic acid (EDTA (Sigma‐Aldrich)) at room temperature for 30 min.
3. Wash three times with HPLC water then incubate with 1,200 μl of 10 mM FeCl_3_ end‐over‐end at room temperature for 30 min.
4. Wash the agarose beads again three times with HPLC water and resuspend with 1:1:1 acetonitrile: methanol: 0.01% acetic acid to a ratio of 1:3 beads to slurry volume.
5. Aliquot 60 μl slurry, or 20 μl beads, into 1.5‐ml Eppendorf tubes for each 1 mg sample undergoing phosphopeptide enrichment.
6. Reconstitute the dried peptide aliquots in 0.1% TFA / 50% ACN and vortex until the peptides were fully dissolved. Add 0.1% TFA / 100% ACN to each aliquot to bring the final concentration to 0.5 mg/ml in 80% ACN solution.
7. Add 30 fmol of heavy‐labeled IMAC peptides into each sample, and then add the peptide solutions on top of the prepared beads and incubate end‐over‐end for 30 min at room temperature.
8. Spin down the beads for 1 min at 1,500 rcf. Remove the supernatant and save.
9. Add 200 μl of 0.1% TFA / 80% ACN to the beads. Transfer onto a prepared stagetip for desalting.

#### Immobilized metal affinity chromatography stage‐tip desalt

1. Condition the stagetips, prepared with two Empore C18 (3 M) punches, first with 100 μl of methanol, then with 50 μl of 0.1% FA / 50% ACN and spin down at 3,100 rcf for 1 min after each step.
2. Equilibrate the tips with 2 injections of 100 μl 1% FA.
3. Load the resuspended beads onto the stagetips and spin down.
4. Desalt the stagetips with two 50 μl injections of 0.1% TFA / 80% ACN then one 50 μl injection of 1% FA.
5. Elute the phosphopeptides off the agarose beads and onto the stagetips by three 70 μl injections of 500 mM K_2_PO_4_.
6. Wash the samples once with 100 μl of 1% FA,
7. Elute the samples off the tips with 60 μl 0.1% FA / 50% ACN.
8. Transfer the eluates from Eppendorf tubes to HPLC vials, freeze, and dry down by vacuum centrifugation.
9. Reconstitute in 9 μl of 0.1% FA / 3% ACN solution and inject 4 μl for LC‐MRM/MS analysis on TSQ Quantiva using IMAC LC‐MRM/MS method (see above).

##### Titration curve experiment

Five cell lines (OVCAR, Meljuso, H3122, PC9, and A375) were lysed, digested, and desalted as described above. Equal amounts of peptides from each digest were combined to create the peptide mix used in the study. In triplicate, 1 mg and 5 mg aliquots of this peptide mix were reconstituted in 1.5 ml IAP buffer (50 mM MOPS/NaOH pH 7.2, 10 mM Na_2_HPO_4_, 50 mM NaCl). Aliquots were then spiked with 30 fmol of the pY and IMACpY heavy peptide mixtures, then enriched by pY 1000 antibody using the phosphotyrosine enrichment method detailed above. Enriched samples were stagetip‐desalted (see method above) then analyzed on TSQ Quantiva using the method for pY LC‐MRM/MS described above. The flow‐through samples from the pY Ab enrichments were combined and desalted together. The resulting mix was aliquoted in triplicate at 1, 0.5, 0.25, 0.1, and 0.05 mg. Each aliquot was reconstituted in 1 ml 0.1% TFA / 50% ACN (1 mg aliquots were reconstituted in 2 ml) then spiked with 30 fmol of the IMAC heavy peptide mixture. The IMAC phosphopeptide enrichment procedure detailed above was used. Enriched samples were stagetip‐desalted (see method above) then analyzed on TSQ Quantiva using the method for IMAC LC‐MRM/MS described above.

##### Cell line processing for testing the assay

Ten cell lines (PC9, H3122, TMD8, Mino, PC3, OVCAR4, WM266.4, Meljuso, A375, and RT112) were lysed and digested as described above. Five milligrams of each was enriched by pY antibody and 1 mg of the flow‐through of that was enriched by IMAC and analyzed following SigPath workflow as described above.

##### Cell line perturbagen sample processing

H3122 and Ls513 cells were treated with either DMSO or drug for 6 h and 24 h (Fig EV3A). H3122 cells were treated with Ceritinib at 300 nM concentration, and Ls513 cells were treated with Trametinib at 30 nM concentration. Treatments and time points were done in two process replicates. Cells were collected, lysed, and digested as described above. Following digestion, 5 mg of each sample was enriched with pY Ab and 1 mg of the flow‐through of that by IMAC and analyzed according to the SigPath workflow described above.

##### Breast cancer xenograft (PDX) tissue processing

Six models were selected from Washington University human to mouse (WHIM) PDX collection (Mundt *et al*, 2018) (4, 30, 21, 6, 2, and 12). Each of the models was treated either with buperlasib or with vehicle (Fig 3A). For the 2‐h treatment group, the animals received one dose and tissue was collected 2 h after the treatment either by buperlasib or by vehicle. For the 50‐h group, animals received Buperlasib or vehicle at 0, 24, and 48 h, and the tissue was collected at 50 h. Only in the washout group at 48 h, the animals were treated with vehicle instead of the drug. Tissue lysis and digestion were performed as described above. Input peptide amount for pY Ab enrichment varied as follows for the different WHIM models due to limited amount availability of some of the samples: WHIM4 – 4.5 mg; WHIM30 – 4.5 mg; WHIM21 – 5 mg; WHIM6 – 5 mg; WHIM2 – 2 mg; WHIM12 – 4 mg. Input peptide amount for IMAC was 1 mg for all of the samples. Phosphotyrosine antibody enrichment for all the five samples of each WHIM was done the same day, but on different days for the different WHIMs. IMAC enrichment was performed over 3 days, samples for 2 WHIM models per day.

##### Medulloblastoma tissue processing

39 tissue samples from medulloblastoma patients belonging to all four groups (sonic hedgehog (SHH), group 3 (GR3), group 4 (GR4) and WNT) were digested as described above. Less than 1 mg of digested peptides was available for this study, and therefore, the pY Ab portion of the procedure was skipped and only the IMAC part of the assay was applied to all the samples with 500 μg input digested peptides for 85% of them. For 6 of the samples, 500 μg was not available; therefore, the peptide input varied as follows: MB088 (SHH) = 357 μg; MB136 (SHH) = 347 μg; MB206 (SHH) = 313 μg; MB284 (SHH) = 444 μg; MB287 (SHH) = 425 μg; MB091 (GR4) = 485 μg. Sample processing and data acquisition were performed in 4 batches. Samples were randomized in 4 batches making sure to include an equal number of samples from each group.

#### Data processing

All analyses of raw mass spectrometry data were performed in Skyline Targeted Mass Spec Environment (Broudy *et al*, 2014). Peak area ratios of endogenous light to stable isotope‐labeled (SIL) heavy internal standard peptide were calculated in Skyline (Skyline version (64‐bit) 20.2.0.343), https://brendanxuw1.gs.washington.edu/labkey/project/home/software/Skyline/begin.view). All the peaks were manually inspected to make sure accurate and equal integration of light and heavy versions. Peak area ratios of the most abundant, interference‐free transitions were used for further statistical analysis. Detection of endogenous (light peptide) signals required detection of all the transitions along with a minimal signal for the reporting transition based on manual inspection of the data. The height of the light peak had to be more than 250 and 300 counts for pY Ab and IMAC enriched samples, respectively.

#### Data imputation

Imputation of missing data points was performed for the perturbagen experiments (H3122, Ls513, and PDX), separately for each dataset and pY Ab / IMAC experiments. Phosphopeptides were divided into three categories: 1) confidently quantified peptides derived as described above; 2) quantified peptides not passing the minimal signal threshold; and 3) peptides not quantified at all. Log_2_‐transformed peptide peak area ratios for 3) were imputed by drawing from a normal distribution with parameters *mu* and *sd* (mean and standard deviation, respectively) estimated from the distribution of light/heavy ratios in 2). Parameter *mu* was further adjusted by subtracting one *sd* to resemble light/heavy ratios below the detection limit.

For statistical analysis of these datasets, peptide ratios of category 2) were kept in the dataset and imputed values for category 3) were used.

#### Statistical analysis

Peak area ratios were log_2_‐transformed. Visual inspection of the resulting log‐ratios in profile density plots revealed comparable distributions of samples within each dataset, and no further normalization was applied. A two‐sample moderated *t*‐test was applied to cell line perturbagen, medulloblastoma, and PDX datasets comparing sensitive versus resistant models using Protigy. (https://github.com/broadinstitute/protigy). Derived *P*‐values were adjusted for multiple hypothesis testing using Benjamini–Hochberg (BH) strategy. Significance was assessed with BH‐corrected *P*‐value of < 0.1.

A one‐sample moderated *t*‐test was applied to drug/vehicle ratio of ratios in the PDX dataset to compare drug to vehicle treatment for all 6 PDX models. Derived *P*‐values were adjusted for multiple hypothesis testing using Benjamini–Hochberg (BH) strategy. Significance was assessed with BH‐corrected *P*‐value of < 0.1.

A one‐way ANOVA with an *ad hoc* Tukey’s test (with (BH) adjusted *P*‐values for multiple comparisons) was performed in GraphPad Prism on medulloblastoma dataset.

Heat maps are generated using the Morpheus online tool (Morpheus, https://software.broadinstitute.org/morpheus

#### CausalPath analysis

CausalPath (Babur *et al*, 2021) analysis was used to identify the likely cause–effect relations between the correlated phosphopeptide measurements. For the comparison of drug/DMSO at 6 h and 24 h in H3122 and Ls513 cell lines, sensitive versus resistant in PDX study and groups in medulloblastoma study, BH‐corrected *P*‐values from a two‐sample moderated *t*‐test were used as input to the method. For the comparison of drug versus vehicle in PDX study at 2 and 50 h, BH‐corrected *P*‐values from a one‐sample moderated *t*‐test were used as input to the method. The method options to calculate network significance and to use the inferred activities in causal reasoning are turned on. FDR cutoff of 0.1 was used for both phosphopeptide change significance and network significance. For the drug inhibition studies, custom hypotheses indicating inhibition of direct drug targets were inserted. This allows CausalPath to identify the changes that are compatible with the hypothesis and use them in the resulting model. ChiBE (Babur *et al*, 2014) was used as a visualization tool to generate Fig 3B.

## Author contributions

HK, ERM, DAP, KW, WRS, and SAC conceived the study; HK, ERM, DAP, JJ‐V, FM, and SAC designed the study; HK, RM, LW, HS, FM, DF, BR, SEM, MLR, MB, MAM, and MEO generated the data; HK, KK, FM, SS, and DRM did formal analysis; KK, DRM, and OB provided and helped with software; ERM, DAP, JJ‐V, PMJB, OB, SS, EK, ET, MAG, WRS, and SAC provided resources; HK, ERM, DAP, FM, KK, JG, SS, DRM, MAG, and TR curated the data; HK, RM, FM, KK, SS, and SAC wrote the original draft; HK, FM, KK, SS, MG, TR, KW, WRS, and SAC reviewed and edited the manuscript; HK, ERM, JJ‐V, KW, WRS, and SAC supervised the study; WRS and SAC acquired the funding.

## Conflict of interest

SAC is a member of the scientific advisory boards of Kymera, PTM BioLabs, and Seer and a scientific advisor to Pfizer and Biogen.

## Supporting information



Expanded View Figures PDF

Dataset EV1

Dataset EV2

Dataset EV3

Dataset EV4

Dataset EV5

Dataset EV6

Dataset EV7

## Data Availability

The datasets and computer code produced in this study are available in the following databases: All mass spectrometry data generated during this study have been published in Panorama (Sharma *et al*, 2018) and are deposited at: https://panoramaweb.org/YyIDIy.url. The source code for Protigy used for all statistical analysis is on GitHub: https://github.com/broadinstitute/protigy. The data for CausalPath analysis have been deposited at GitHub: https://github.com/broadinstitute/proteomics‐SigPath‐supplemental‐data.
