# Supplementary material for: A highly multiplexed quantitative phosphosite assay for biology and preclinical studies
Source: Mol Syst Biol. 2021 Sep 27;17(9):e10156. doi: 10.15252/msb.202010156 (PMC8474009; doi:10.15252/msb.202010156)
Supplement: Supplementary file 1 — Expanded View Figures PDF [file MSB-17-e10156-s008.pdf]

Expanded View Figures

Figure EV1. Development of SigPath assay.

A Process for selecting phosphosites and phosphopeptides for SigPath assay development. Majority of phosphosites were nominated by experts, then converted into tryptic peptides, searched against existing datasets at the Broad for detection of them in MS data (see Materials and Methods section). One-fourth of the phosphosites were included based on them being differentially regulated in quantitative phosphoproteomic studies (Mertins *et al*, 2014). Once finalized [C13, N15], stable isotope-labeled versions of the peptides were synthesized for the assay.

B Assay configuration and testing statistics of SigPath. Twenty-four out of 352 peptides failed the assay configuration due to their LC or MS characteristics, while another 30 failed during the pY Ab or IMAC enrichment step. Final SigPath assay targets 298 phosphopeptides with 284 phosphosites.

C Pie graph showing range of phosphosites per protein in the assay panel. 71% of the proteins are represented by only one phosphopeptide, 19% by two phosphopeptides. The remaining varies from 3 to 9 phosphopeptides.

D Pathways represented by SigPath in MSigDB Hallmark pathway category. To be included in the plot, a pathway had to have at least 5% coverage, or be represented by a minimum of three proteins and five phosphosites in the assay. Both, number of genes (red) and phosphosites (pink) are shown on the plot. [1]Included from MSigDB WikiPathway pathway category.

© 2021 The Authors

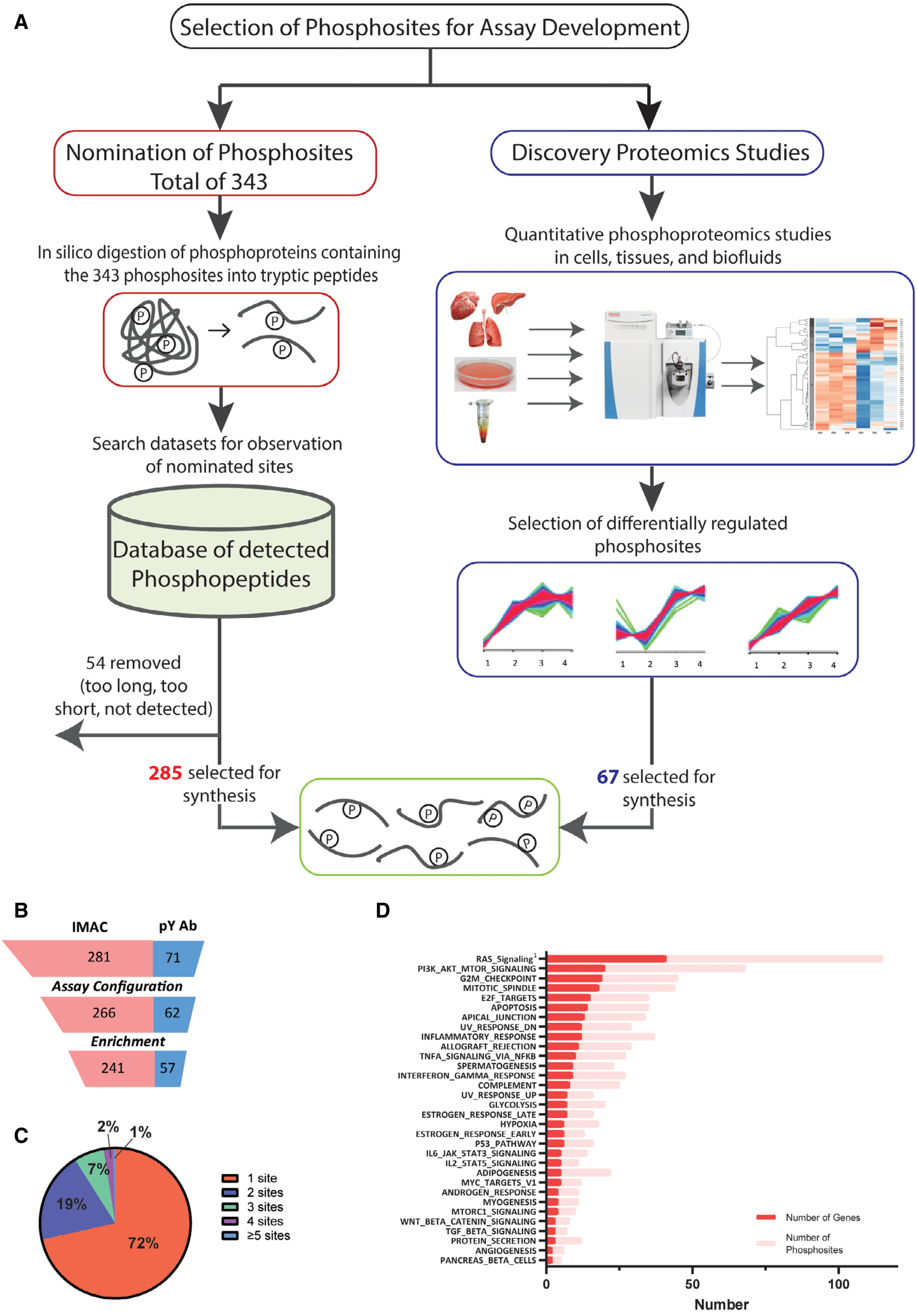

Figure EV1.

 © 2021 The Authors

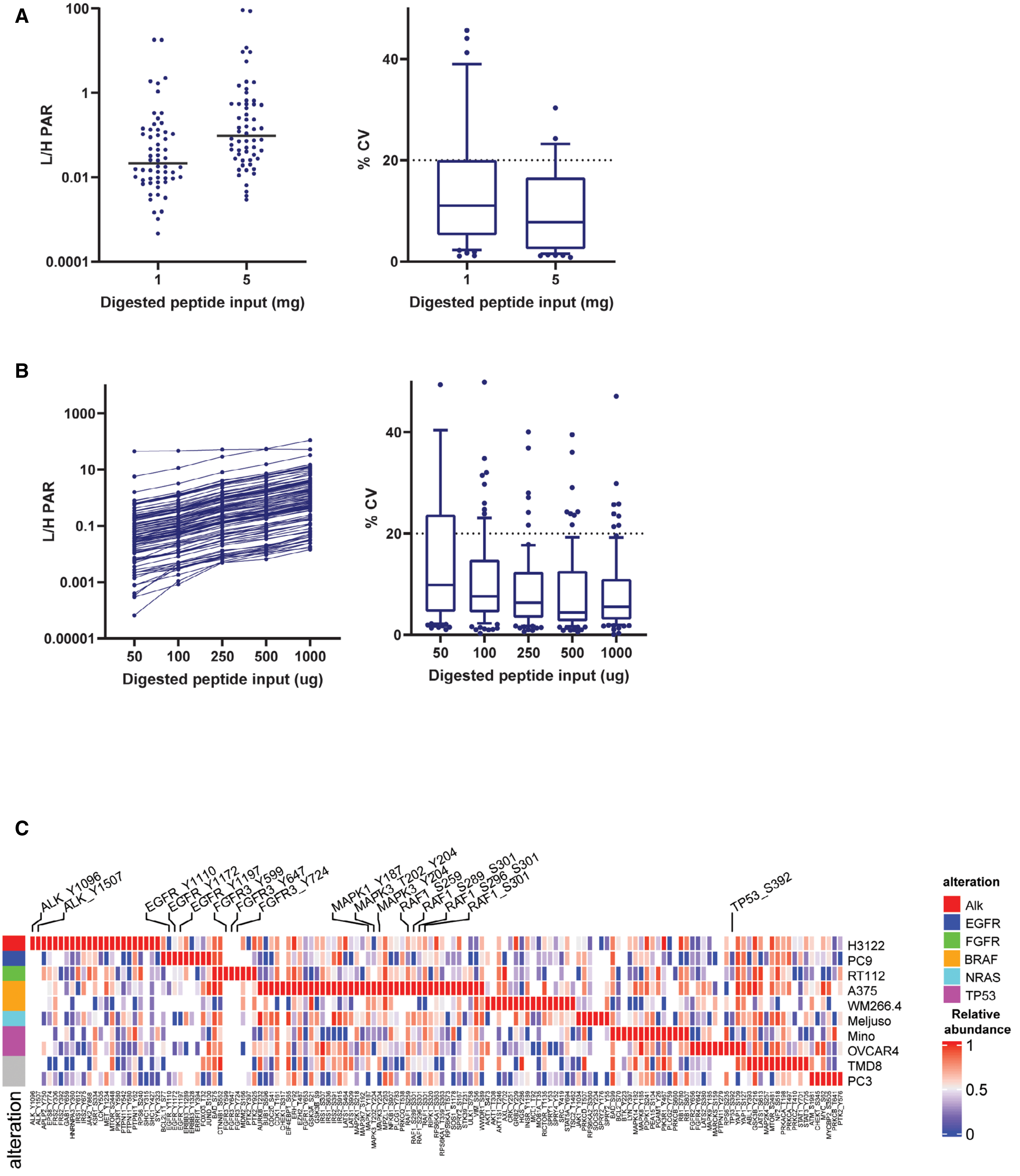
A
L/H PAR
100
1
0.01
0.0001
1
5
Digested peptide input (mg)
% CV
40
20
0
1
5
Digested peptide input (mg)
B
L/H PAR
1000
10
0.1
0.001
0.00001
50
100
250
500
1000
Digested peptide input (ug)
% CV
40
20
0
50
100
250
500
1000
Digested peptide input (ug)
C
ALK_Y1096
ALK_Y1507
EGFR_Y1110
EGFR_Y1172
EGFR_Y1197
FGFR3_Y599
FGFR3_Y647
FGFR3_Y724
MAPK1_Y187
MAPK3_T202_Y204
MAPK3_Y204
RAF1_S259
RAF1_S289_S301
RAF1_S296_S301
RAF1_S301
TP53_S392
H3122
PC9
RT112
A375
WM266.4
Meljuso
Mino
OVCAR4
TMD8
PC3
alteration
alteration
Alk
EGFR
FGFR
BRAF
NRAS
TP53
Relative abundance
1
0.5
0

Figure EV2.

Figure EV2. Evaluation of the SigPath assay.

A Average light/heavy peak area ratio and %CV of the three replicates for all quantified peptides in titration curve experiment after pY Ab enrichment. Plot on the left shows the average values for all the quantified peptides along with a line representing the median value at 1 and 5 mg input protein level. The plot on the right shows the spread of %CVs at 1 and 5 mg input protein. The box represents interquartile range (IQR) with the lower, central, and the upper bands representing 25th percentile (Q1), median, and 75th percentile (Q3), respectively. The whiskers extend from 10 to 90 percentile of the data.

B Average light/heavy peak area ratio and %CV of the three replicates for all quantified peptides in titration curve experiment after IMAC enrichment. Plot on the left shows the average values for all the quantified peptides along with a line representing the median value at 0.05, 0.1, 0.25, 0.5, and 1 mg input protein level. The plot on the right shows the spread of %CVs at all the input protein levels. The box represents interquartile range (IQR) with the lower, central, and upper bands representing 25th percentile (Q1), median, and 75th percentile (Q3), respectively. The whiskers extend from 10 to 90 percentile of the data.

C Heat map showing relative abundances of all detected phosphosites across the 10 cell lines. Labeled are phosphosites on known driver genes in these cell lines. One replicate of each samples was processed and analyzed for this experiment. The heat map was generated using Morpheus online tool, the data are median-MAD normalized, and colors are relative across rows, from row min to row max.

Figure EV3. Application of the assay to cancer cell line perturbagen experiments.

A Experimental design and details for drug treatment studies in H3122 and Ls513 cell lines. Table contains details about the cell lines as well as the inhibitor and concentration of it used. Cells were treated either with the inhibitor or DMSO for 6 and 24 h. Two process replicates were collected for each sample.

B Inhibition of pALK and pERK signaling in established human cell lines. Immunoblot analyses of cultured H3122 lung adenocarcinoma cells (on the left) treated with ALK inhibitor Ceritinib (+) or DMSO (−) for 6 and 24 h. Antibodies recognizing the phosphorylated 1507-Tyrosine site of the ALK protein and the Actin protein (loading control) were used. Immunoblot analyses of cultured Ls513 colorectal carcinoma cells (on the right) treated with KRAS inhibitor Trametinib (+) or DMSO (−) for 6 and 24 h. Antibodies recognizing the phosphorylated Thr 185/Tyr 18 sites of the ERK1/ERK2 proteins and the vinculin protein (loading control) were used.

C Scatter plot of two process replicates of Log_2 light/heavy PAR of each sample group. Shown on each plot is the Pearson correlation coefficient.

D Scatter plot of light/heavy peak area ratio of peptide 1 and peptide 2 measuring the same site for 12 of the sites measured in H3122 and Ls513 perturbagen experiments. X-axis and y-axis represent light/heavy peak area ratios. Shown on the graph is Pearson correlation coefficient.

 © 2021 The Authors

A

Cancer Type	Cell Line	Alteration	Target	Inhibitor	Conc. Of the Inhibitor
Lung	H3122	ALK-fusion	ALK	Ceritinib	300nM
Colon	Ls513	KRAS	MEK	Trametinib	30nM

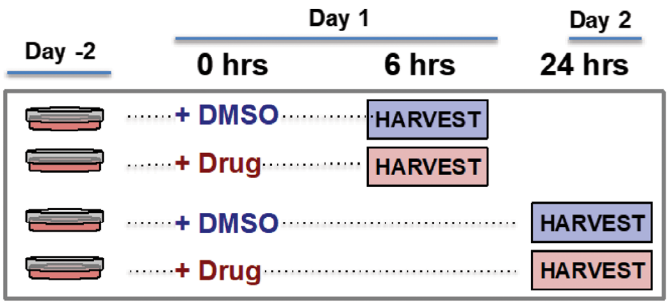

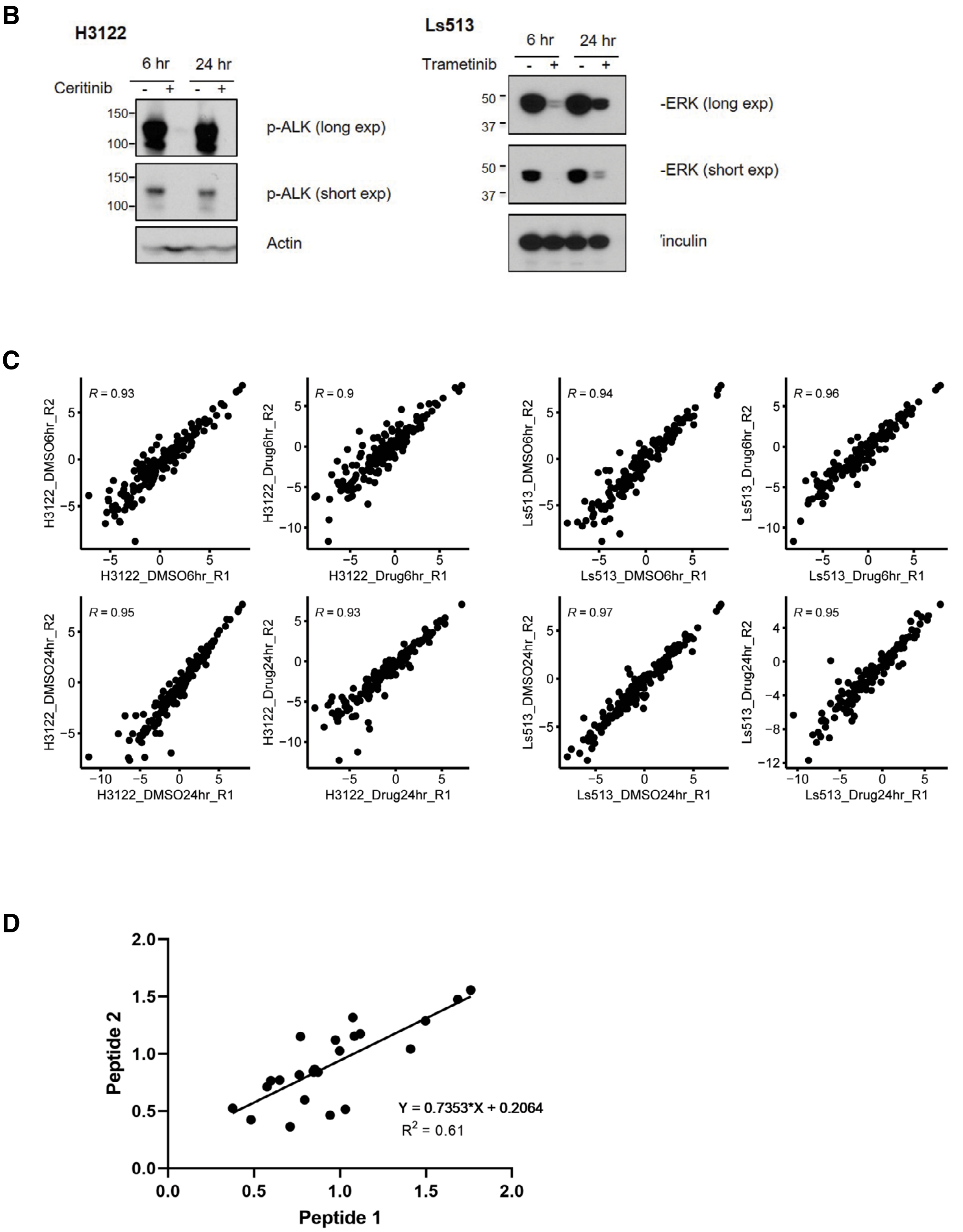

Figure EV3.

Figure EV4. Application of the assay to breast cancer PDX tissue samples.

A Levels of AKT pS473, AKT pT308, AKT1S1 pT246, and AKT2 pS474 which are all pharmacodynamic markers for PI3K inhibition observed using SigPath. For each graph, and treatment in each graph, the WHIM/PDX models are sorted in the order of their resistance to the drug with least resistance on the left. Across all markers, the most resistant model (WHIM12) is the least affected at 50 h of buparlisib treatment. *P*-values, in round brackets "()", are calculated from a one-sample *t*-test, compared to a hypothetical mean of 0, using all WHIMS as replicates for a certain treatment.

B Bar and whisker plot showing the range of ratios obtained for the overlapped 115 peptides in discovery study using TMT and SigPath. For the discovery study, Log_2 ratio of all the peptides to the pooled reference was used for the plot. For SigPath, Log_2 ratio of light to heavy peptide was used for the plot.

 © 2021 The Authors

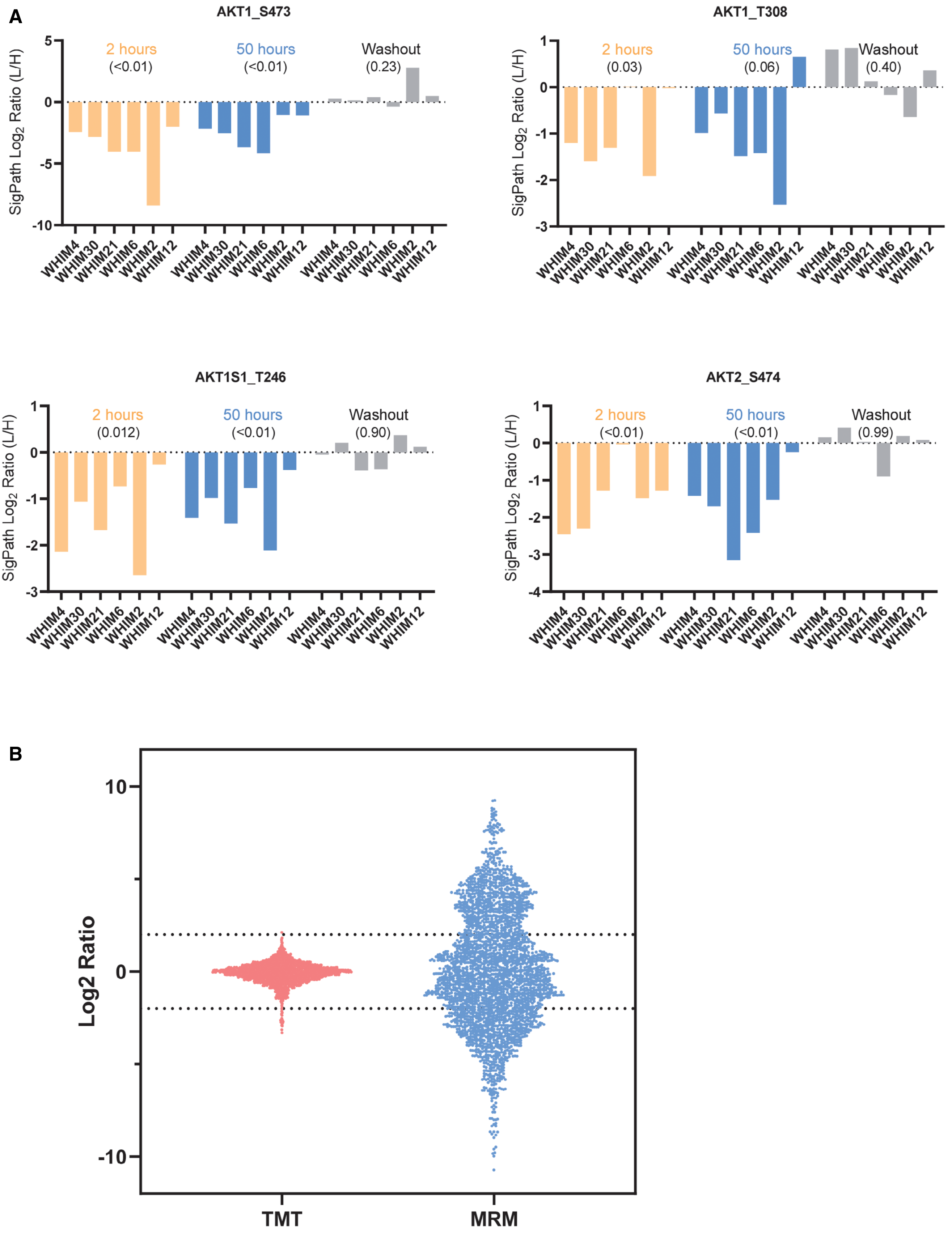
A
AKT1_S473
2 hours
(<0.01)
50 hours
(<0.01)
Washout
(0.23)
SigPath Log2 Ratio (L/H)
AKT1_T308
2 hours
(0.03)
50 hours
(0.06)
Washout
(0.40)
AKT1S1_T246
2 hours
(0.012)
50 hours
(<0.01)
Washout
(0.90)
AKT2_S474
2 hours
(<0.01)
50 hours
(<0.01)
Washout
(0.99)
WHIM4
WHIM30
WHIM21
WHIM6
WHIM2
WHIM12
B
Log2 Ratio
TMT
MRM

Figure EV4.

Figure EV5. Application of the IMAC subset of the SigPath assay to human tissue samples from medulloblastoma patients.

A List of samples used for this experiment and their classification to SHH, GR3, and GR4 as well as subgroups within SHH and GR3 (Archer *et al*, 2018). Only IMAC subset of the assay has been applied to these samples.

B Venn diagram showing the overlap of the 140 sites detected in SigPath assay with the discovery data. 86 peptides were detected in both datasets, 58 of these were detected in the discovery dataset in all samples (dark blue) while another 28 detected in at least 9 samples (light blue) of the discovery dataset. 54 sites (39%) were unique to SigPath assay (violet).

C Dendrogram illustrating the clustering of MRM (dark leaves) and discovery data (light leaves) of 86 phosphosites detected in both assays. Colors of sample identifiers are coordinated by patients. Log_2 TMT ratio of each sample to the pooled reference was used for the discovery data after median-MAD normalization. Log_2 light/heavy ratios of SigPath data were used after normalizing each peptide by the median log_2 light/heavy ratio across all samples. The dendrogram was derived from complete-linkage hierarchical clustering using 1-Spearman correlation as the distance metric. All sample pairs (MRM/discovery) cluster adjacent to each other.

 © 2021 The Authors

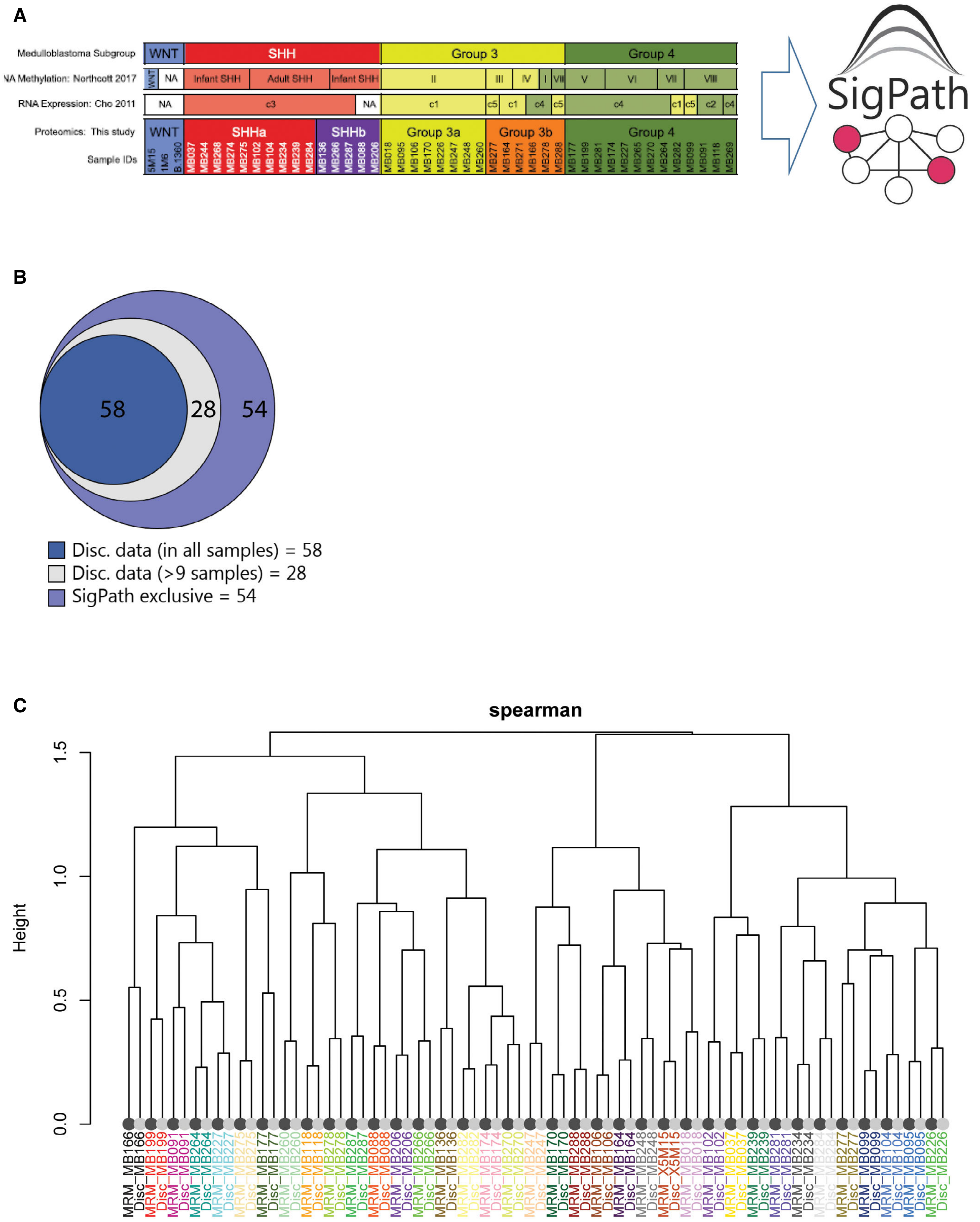
A
Medulloblastoma Subgroup
NA Methylation: Northcott 2017
RNA Expression: Cho 2011
Proteomics: This study
Sample IDs
WNT
SHH
Group 3
Group 4
Infant SHH
Adult SHH
NA
c3
c1
c4
c5
c2
SHHa
SHHb
Group 3a
Group 3b
SigPath
B
58
28
54
Disc. data (in all samples) = 58
Disc. data (>9 samples) = 28
SigPath exclusive = 54
C
spearman
Height
0.0
0.5
1.0
1.5

Figure EV5.
