# Supplementary material for: A highly multiplexed quantitative phosphosite assay for biology and preclinical studies
Source: Mol Syst Biol. 2021 Sep 27;17(9):e10156. doi: 10.15252/msb.202010156 (PMC8474009; doi:10.15252/msb.202010156)
Supplement: Supplementary file 9 [file MSB-17-e10156-s004.pdf]

Highly multiplexed quantitative phosphosite assay for biology and preclinical studies

Hasmik Keshishian, E. McDonald III, Filip Mundt, Randy Melanson, Karsten Krug, Dale Porter, Luke Wallace, Dominique Forestier, Bokang Rabasha, Sara Marlow, Judit Jane-Valbuena, Ellen Todres, Harrison Specht, Margaret Robinson, Pierre Jean Beltran, Ozgun Babur, Meagan Olive, Javad Golji, Eric Kuhn, Michael Burgess, Melanie MacMullan, Tomas Rejtar, Karen Wang, DR Mani, Shankha Satpathy, Michael Gillette, William Sellers, and Steven Carr
DOI: 10.15252/msb.202010156

Corresponding authors: Hasmik Keshishian (hasmik@broadinstitute.org) , Steven Carr (scarr@broadinstitute.org)

Review Timeline:

Submission Date:	4th Dec 20
Editorial Decision:	20th Jan 21
Revision Received:	24th Jun 21
Editorial Decision:	6th Aug 21
Revision Received:	19th Aug 21
Accepted:	20th Aug 21

Editor: Maria Polychronidou

Transaction Report:

(Note: With the exception of the correction of typographical or spelling errors that could be a source of ambiguity, letters and reports are not edited. Depending on transfer agreements, referee reports obtained elsewhere may or may not be included in this compilation. Referee reports are anonymous unless the Referee chooses to sign their reports.)

20th Jan 2021

RE: MSB-2020-10156, Highly multiplexed quantitative phosphosite assay for biology and preclinical studies

Thank you again for submitting your work to Molecular Systems Biology. We have now heard back from the three referees who agreed to evaluate your study. Overall, the reviewers acknowledge that the study seems potentially interesting. They raise however a series of concerns, which we would ask you to address in a major revision.

Without repeating all the comments listed below, some of the more fundamental issues are the following:

- The advance compared to previous related approaches needs to be better described and the potential pitfalls/challenges need to be discussed.
- Some follow up analyses providing biological context for the results obtained using SigPath would need to be performed. Reviewers #2 and #3 provide constructive suggestions in this regard (e.g. reviewer #2 points #3 and #8 and reviewer #3 point #4). Addressing this point is important as it would enhance the confidence regarding the ability of the approach to generate biologically relevant findings.

All issues raised by the reviewers need to be satisfactorily addressed. As you may already know, our editorial policy allows in principle a single round of major revision, so it is essential to provide responses to the reviewers' comments that are as complete as possible. Please contact me in case you would like to discuss in further detail any of the issues raised.

On a more editorial level, we would ask you to address the following points:

Reviewer #1:

Keshishian et al. present the most comprehensive targeted proteomics workflow for quantifying phospho-peptides in biological samples. It was designed for more than 300 phosphosites, of which more than 250 can be reliably targeted. This compares to assays with similar technology published some years ago, that aimed to target about 100 phosphosites.
The authors follow a process to determine the phosphosites to be monitored to allow a broad read out of signaling activity. The collection of heavy labelled phospho-peptides is synthesized and spiked into each sample. This sounds simple in theory but is quite difficult to control. The authors should comment on how accurately they were able to quantify the synthetic peptides, how stable they are and in what ratios they are combined. Are these phospho-peptides available to others and if not, how difficult would it be to re-create them?
The authors put the assay through its paces by first measuring cell lines with stimulation, PDX models and finally tumor material. There is mention of reproducibility such as rank order consistency but relatively little documentation of quantitative accuracy on the phospho-site level, which could be specifically addressed when several peptides cover the same site.
The idea with such targeted PTM assays is that they should allow very consistent read out of phosphosites, be quite quantitative and ideally high throughput and easy to do. They sit between label-free assays and TMT-assays. Especially the label-free assays with DIA methods on high resolution instruments have been gaining ground, whereas the author's technology is quite mature and still uses low resolution instruments (triple quadrupoles). As the Carr group also performs dia, it would have been interesting to see a comparison between them, especially in quantitative accuracy and completeness.
Overall, this is a substantial amount of work with many interesting aspects to it. The approach is not novel but has been under development for at least 10 years. Given the care that needs to be taken with the spike in peptides, an overall question would be if it is not easier to go with dia based method, which achieve very good CV values today. However, if the authors concentrated on the many points that are valuable to the community, this manuscript could still be a valuable addition to the literature.

Organization of the paper: The authors should move a lot of the material from the back of the paper to the front, especially where they explain their assay, its limitations and its context. This would help the reader. They should also state in the main text that it is an MRM assay on a triple quadrupole. This would also be a good place to mention practical aspects like throughput (2-3h gradients?), what is necessary to create the library, costs, stability etc. They should also mention amounts needed, that the assay does not correct for protein level changes, quantitative accuracy expected and so on, in the beginning before they go to biological applications. The authors also claim 85% success rate but this can be much lower depending on the amount available, as they show later.

The authors cite their own efforts in MRM assay development and the CPTAC consortium extensively but are less inclusive in citing work outside. In the supplementary material, the authors say that all MS data was published in 2018, which is confusing.

Specific points:
1. The title should be changed. E.g. Multiplexed phosphosite detection assay for...

2. Phosphosites of the assay (Figure 1C and 1D) should be depicted in a signaling pathway scheme.
3. Describe the assay in the main text (Figure 1A) and the figure legend has to be explained clearly.
4. Conclusions drawn from the correlation in Figure 2B should be described in the main text.
5. Exchange Figure 4B on page 8 with Figure 2B.
6. Figure 2A legend contains the same sentence twice.
7. 2C, 2D has to be better explained in the text. It is not clear that Y542 and Y580 are outliers in patient samples with ALK fusion; also, it looks like Figure 2C has a different pt of the lines than Figure 2D.
8. Was the proteome of the cell line and PDX samples measured to check whether changes in phosphorylation underlie proteome changes? In general, can authors also target nonphosphorylated peptides on the same proteins and measure their levels before enrichment to investigate whether changes in phosphosite levels can be just attributed to protein level differences instead of analyzing TMT proteome data (like done in Figure 4B)? Proteome data must also suffer from ratio compression.
9. If a figure in the text is described, please mention the figure as well and make sure it is in the correct position, e.g. the description of Trametinib treatment outcome (Figure 2A) (page 9) or the description of the sensitivity for the PDX models (Figure 3A).
10. Figure legend for S3: The last sentence should be finished.
11. Figure 3C, 4A, S2A: What are the units/legends? Figure 3D, are fold changes on log2 scale?
12. Were PTPN11 substrates measured? Are they differentially phosphorylated upon treatment or in cells harboring ALK fusions and EGFR mutants?
13. In light of the amount of material required for phosphoenrichment, how practical is the approach for clinical/biological applications?
14. Another limitation of their assay would be its throughput when it is applied to large sample collections. It utilizes and requires two laborious steps to enrich phosphopeptides to quantify them with high sensitivity. Furthermore, each LC-MRM/MS run takes considerable time (160min for IMAC method and 120min for pY method), allowing them to analyze only five samples per day. How possible is it to shorten gradients and simplify the workflow in future? This should be discussed in the manuscript in light of current developments in technology. Authors may consider revising their following sentence 'Sample preparation for SigPath is much simpler than methods commonly used for deep-scale discovery...' as several streamlined phosphoproteomics platforms for in-depth analysis have been described in the literature.
15. What is the assay quality without phosphoenrichment? Are enrichment procedures automated? If so, in which platform were they performed?
16. Figure legend 4B: was Pearson intended instead of Pierson correlation?
17. Figure 4B: Is the fold change indicated? This should be added to the figure legend.
18. The precision and reproducibility of the assay should be explored further; for instance, workflow and analytical variabilities can be determined.
19. Were SIL peptides AQUA peptides with known quantities? It is unclear if the authors determined their purity and asses light peptide contamination levels.
20. Please read the manuscript carefully and remove all the typos.

Reviewer #2:

In this manuscript, Keshishian et al design a MS-based assay to systematically detect and quantify

phosphorylation events in key regulatory proteins across a range of signaling pathways. They selected and curated a list of candidate peptides inferred from the literature, and combined this with available large-scale phosphoproteomics data to eventually compile an assay termed SigPath that spans 284 phosphosites in 200 phosphoproteins. They apply the assay to monitoring changes in the targeted phosphoproteome in cancer cells upon drug treatment, in cancer xenografts, and in tumor tissues. They identify phosphorylation events that were expected for the treatment or condition, in addition to novel ones that were unexpected and (in part) not detected in previous unbiased approaches. This leads the authors to conclude that SigPath should be a useful assay to examine the activation status of signaling pathways in various clinical and pre-clinical scenarios.

This is an interesting study with a clear rationale, namely that it may be more informative to accurately quantify a limited set of biologically important phosphorylation events, instead of doing this in a more classical unbiased way identifying thousands of phosphopeptides however whose function and relevance is less clear. Setting up such an assay is a considerable effort, requiring the selection of phosphopeptides (based on prior knowledge), optimization of MS conditions to reliably detect these peptides, and synthesis of isotope-coded versions of these peptides to be spiked into biological samples for quantification of endogenous phosphopeptides. Indeed, and rightfully, the description of the assay development takes a considerable portion of the manuscript.

Some critical issues remain as to the novelty of the manuscript, both conceptually using multiplexed MRM as an assay for cellular signaling activity, and with regard to biological findings. This paper is not the first to develop such an assay, two of which are referred to in the manuscript (Abelin et al, and Kennedy et al), where it remains a bit unclear what SigPath adds especially with regard to the Abelin tool (P100). Also there is some previous work that was not cited. With regard to the applications of SigPath that are shown, this reviewer had some mixed feelings about the few observations that were highlighted from the data set, against a range of results that remained uncommented. In particular, it is unclear why so little consistency was observed both in the drug-treated xenograft mice and among the sampled breast cancer phosphoproteomes. It either means that the assay is less conclusive than anticipated, or the diversity in signaling activity (both qualitatively and quantitatively) is much bigger than initially thought, which would be a sobering conclusion. Either way, it would have been advantageous if this had been exposed/investigated more deeply to point to potential weaknesses in the assay, or to challenges that to be addressed to increase our general understanding of cellular signaling.

Specific comments:

1. Abelin et al used a very similar strategy to design an MRM assay (termed P100, later used in a more elaborate application PMID 29655704) with a very similar target space as SigPath. How do P100 and SigPath differ, and why was it necessary to develop SigPath having P100 at hand? And why was it needed to build and optimize SigPath from scratch, having much of the foundation (probably some shared peptides/phospho-sites) already in place? What can SigPath do that P100 cannot?
2. Apart from Abelin et al and Kennedy et al, Picotti et al were probably the first to introduce the concept of a MRM-based 'sentinel assay' 6 years ago (PMID: 25194849), and to implement this at a very similar scale as in this manuscript. In particular, it included 157 proteins and 152 phosphopeptides in a multiplexed fingerprint assay to report on the activity of 188 biological processes. Although this was performed in yeast, the study should be highlighted in the manuscript because conceptually it set the stage for SigPath.
3. A main concern about the manuscript is that it treats data obtained by SigPath as individual observations, despite the fact that it was set up to obtain a system-wide perspective of signaling. In addition, a tool like SigPath provides the unique possibility to explore (causal) connectivity

between regulatory events. Unfortunately, this was not attempted, and can be seen as a missed opportunity.
4. Even for the single observations of changes in protein phosphorylation the authors did not go into much (mechanistic) depth. For instance (page 9), they find that phosphorylation of RB1 changes upon MEK-inhibition by trametinib, and suggest this as a path how the drug regulates cell proliferation. This is not entirely unexpected, if not to say that it is very well established that this is one of the key ways how MEK drives proliferation (via CDK4/6, e.g. PMID 28127048)
5. To what extent is the assay portable to other mass spectrometric platforms beyond this particular TSQ Quantiva that was used? This is of particular importance since optimal collision energies may differ between mass spectrometers.
6. Figure 4A: Some samples show extremely high or extremely low phosphorylation levels across many proteins (blue and red vertical streaks in the plot). Can this be assigned to any particular status of the patient the sample originates from, and can something be learned from this? Or can it be excluded that this is a technical artifact (e.g. different sample amount, failing enrichment, age of the sample etc)?
7. Fig 4A: Among the displayed proteins, the authors highlight YAP1 as a potentially interesting protein, representing one of few proteins showing consistent expression/phosphorylation in the subgroups. For the remainder of the data, the general impression from the figure is that phosphorilation status is highly heterogeneous across these samples, and even within patient subgoups. Now with the notion that the sampled phosphorylation sites have been carefully selected to reflect biological activity, what does this mean for the signaling status of these patients/tissues, and the utility of (SRM-based) phosphoproteomics for their analysis/understanding? Should any of the conclusions of the discovery paper be revised (Archer et al, 2018), where different kinase activities were associated with disease subgroups?
8. In principle SigPath could have great potential, however it is under-used here. For instance, it would be great to plot the results of a SigPath analysis on the included pathways, visually connect observations, and identifies intermediate signaling hubs that may have been excluded from the panel but that may function as signaling hubs/branching points.
9. PY-enrichment was not used in SigPath for the analysis of medulloblastoma tissue (p11 top of page). The authors should indicate how much material is needed for the assay, anticipating that the required input may be lower than for a regular phosphor-proteomics analysis benefiting from enhanced sensitivity afforded by MRM.
10. The discussion enumerates the conceptual advantages of SigPath, and MRM-based methods in general, to all of which I can agree. However it does not address the key question how its implementation works out in practice, and what can be learned from the data it delivers. For instance, by encompassing key nodes across a wide range of pathways and cellular processes, it should be possible to deduce (causal) relationships between changes in phosphorylation along or across pathways, instead of treating MRMs in the assay as individual measurements. The ability to do so would be a distinct advantage of an integral assay such as SigPath over e.g. western blots that can test far fewer events simultaneously.

Reviewer #3:

Review of Keshishian et al., "Highly multiplexed quantitative phosphosite assay for biological and preclinical studies".

In this manuscript Keshishian and colleagues address an important issue with respect to cell signaling - namely the limited ability of most laboratories to profile more than a few biologically relevant signaling pathways at a time, using convenrtional approaches like Western blotting or other kinase assay-based techniques. While mass spectrometry has emerged as an extraordinarily useful approach to do this, most proteomics labs have concentrated their phosphoproteomic studies on accumulating as many substrates as possible rather than drilling down deeply into the underlying biology of a moderate but important set of known pathways and substrates. Some advances were made using reverse phase protein arrays, but this approach suffers from limited availability of high-quality antibodies, very limited dynamic range, and high background. One of the problems with most phosphoproteomic analysis platforms is the stochastic selection of peptides for analysis, and the complexity of the data, which limits direct comparisons between many samples. The authors build a collection of 298 phosphopeptides representing 284 phosphosites on 280 proteins, of which they appear to be able to detect ~60-70% reliably in subsequent experiments.

Major Comments:

1- Overall, the manuscript is more of a resource or technical article than a true description of new biology. Nevertheless, I think, if the appropriate limitations of the panel are adequately addressed in the text, it makes a very valuable contribution to systems-based studies of signaling, and is appropriate for Molecular Systems Biology.

2- A major shortcoming of the manuscript is the complete absence of a comprehensive signaling diagram showing exactly which proteins and sites are being monitored with the technique. While the selection of phosphosites is, of course, a matter of personal choice, it would have made more sense to ensure that many of the key signaling pathways were represented. As best I can tell from the Supplemental data and Excel spreadsheet, the selection of pathways captures some, but not all, sites on proteins that are critically important in cell decision processes. For example, at least 5 sites on mTOR and half a dozen mTOR substrates are measured, but there are no phosphorylation sites on PI3KR1, DNA-PK, Cdc25A, B, or C, etc. As such, the panel seems to be heavily biased towards measuring signaling in the MAPK, mTOR and Hippo space, with some, but not nearly comprehensive elements of the PI-3K and apoptosis pathways, and other pathways much more lightly sampled. This needs to be explicitly shown using a pathway diagram, or series of pathway diagrams, so that the readers and adopters of the technology will know what is and is not adequately sampled.

3- The statement protein "phosphosites were selected to provide readouts on DNA damage, cell cycle arrest, apoptosis, spindle checkpoint activation, hypoxia, autophagy, cell stress and epithelial-to-mesenchymal transition" seems to overstate the conclusions that can be drawn from what is actually being measured. To really understand the signaling in these pathways many additional components would need to be measured. The sentence should be revised to indicate the preliminary nature of the readouts that the panel provides. It is certainly an overstatement to claim that the readout of a single site on ATM and Chk1, for example, is an adequate proxy for reading out DNA damage and cell cycle arrest for example. Instead, the sites provide some preliminary information that could be used in subsequent experiments to focus on things like DNA damage, cell cycle arrest, etc. and this should be explicitly acknowledged.

4- The panel is then used to examine phosphorylation site changes at 2 time points - 6 and 24 hours in LUAD H1322 cells treated with the ALK inhibitor ceritinib and CRC Ls513cells treated with the MEK inhibitor Trametinib. The findings that the authors observe are consistent with what has

been observed in CPTAC data from patients with these tumor types. However, other than validating that the panel can detect changes, we really learn almost nothing of biological consequence here. The authors should add a diagram mapping the ALK signaling pathway including PTPN11, and the adjacent relevant pathway such as ERBB3, EGFR, and JAK-STAT, showing which sites downstream from ALK on which proteins were the most and least down and up-regulated, which would provide much more biological context for the results. For example, the finding that PTPN11 Y542/580 phosphorylation is reduced might be expected to result in reduced Erk phosphorylation (Miura et al, Oncogene, 2013), yet no such decrease in Erk phosphorylation is observed, possibly due to upregulation of ERBB3 and EGFR. This should be explained in a few more sentences, citing the appropriate references that support this. Can the authors provide some direct evidence of this by simply co-treating the cells with ceritinib plus and ERBB3/EGFR inhibitor and simply blotting for phosphoErk? This would markedly improve the manuscript, clearly extend the mass-spectrometry observations and confirm the tentative conclusions about activation of alternative Tyr kinase receptor pathways.

5- The panel is then applied to six TNBC PDX model samples, with baseline values and values at 2 hours after a single dose, and at 50 hours after treatment with the PI-3K inhibitor buparlisib, aapparent 2 or 3 doses. These studies appear to have been done with one mouse/tumor from each of the PDX samples, but the caption to Figure 3A seems to indicate a total of 30 samples. Can the authors clarify this discrepancy? The data shown in Figure 3 indicates single values of phosphorylation - are these average values from multiple repeats, and if so, how much spread is there in the data?

6- The main thing that one can conclude from this limited study is that the single most resistant PDX model appears to show the least effect of the drug on AKT phosphorylation at three well-established sites. In this regard, the statement "Importantly, the SigPath targeted MS data clearly shows quantitative differences in site modulation between the sensitive and resistant models upon inhibition" seems to distort and overstate the findings since there does not seem to be any obvious correlation between the levels of phosphorylation inhibition following drug treatment of the sensitive versus resistant models except for the single most resistant one.

7- The other conclusion one can draw from this data is that the resistance of some of the PDXs to the PI-3K inhibitor is not mediated at the level of AKT phosphorylation, suggesting the development of resistance arises from other mechanisms, and perhaps this should be explicitly acknowledged.

8- Why is the phosphorylation status of MAPK3 not explicitly shown in Figure 3C, if its activity is what the authors claim is responsible for resistance of the W12 PDX sample?

9- The authors then apply the IMAC approach to query medulloblastoma samples from 38/40 previously analyzed tumors. They focus the text in the results section on two very interesting molecules, YAP and TAZ. Here again, however, the lack of a deeper description of what the results mean, in light of known biology, limits the conclusions they draw. For example, the statement "The Yap1 protein is amplified and upregulated in hedgehog-associated medulloblastomas (Fernandez et al., 2009) while the quantified Yap1 pS127 site indicates inactivation of the protein in this subtype (Artinian et al., 2015)" is only partially correct. Phosphorylation of YAP at Ser-127, and TAZ at Ser-89 results in 14-3-3 binding and cytoplasmic sequestration of these molecules (c.f. Kanai et al. EMBO J, 2000, Zhao et al., Genes and Development 2007) and limits their ability to co-activate TEAD transcription factors that contribute to proliferation. I believe that clarifying and explaining this aspect of the findings provides the reader with a better understanding of what the mass-spec results might mean.

Minor comments

10- The fact that the pY and IMAC mixtures are spiked with labelled peptides should be mentioned early in the Results section.

11- Can the authors comment on whether the panel should be optimally used with some type of isobaric labelling technique for serial time-sampled specimens in future studies?

Response to Editors specific concerns:

- The advance compared to previous related approaches needs to be better described and the potential pitfalls/challenges need to be discussed.
  - We have now added additional information to address this concern as well as one key citation that we overlooked in our original submission.

- Some follow up analyses providing biological context for the results obtained using SigPath would need to be performed. Reviewers #2 and #3 provide constructive suggestions in this regard (e.g. reviewer #2 points #3 and #8 and reviewer #3 point #4).

  - We also found the suggestions from the Reviewers helpful and have now added a significant number of new analyses addressing their concerns.

Reviewer #1:

Keshishian et al. present the most comprehensive targeted proteomics workflow for quantifying phospho-peptides in biological samples. It was designed for more than 300 phosphosites, of which more than 250 can be reliably targeted. This compares to assays with similar technology published some years ago, that aimed to target about 100 phosphosites.
The authors follow a process to determine the phosphosites to be monitored to allow a broad read out of signaling activity. The collection of heavy labelled phospho-peptides is synthesized and spiked into each sample. This sounds simple in theory but is quite difficult to control. The authors should comment on how accurately they were able to quantify the synthetic peptides, how stable they are and in what ratios they are combined. Are these phospho-peptides available to others and if not, how difficult would it be to re-create them?

- The details regarding quantitation and storage of the synthetic peptides have been added to the Peptide Synthesis section of Methods (p25)
- Other than the 24 peptides that failed the assay configuration, the rest of them were stable in solution. This detail was added in the SigPath assay development section of the manuscript on p7 .
- Peptide organization section of methods (p25) contains all the information about mixtures of peptides that were made and used prior to pY and/or IMAC enrichments.

The authors put the assay through its paces by first measuring cell lines with stimulation, PDX models and finally tumor material. There is mention of reproducibility such as rank order consistency but relatively little documentation of quantitative accuracy on the phospho-site level, which could be specifically addressed when several peptides cover the same site.
- To address this question, we have added correlation plots to Figure EV3 for the 12 sites where we measured two different peptide cleavage forms for the same phosphosite. We also added the following sentence to the "Application of the assay to the cancer cell lines with drug treatment" section on p9: "Furthermore, using both LUAD and CRC cell line perturbagen data, we investigated correlation of site quantification as measured by 2

different peptides for the subset of 12 sites for which such data was available. The measured levels of the sites differed by a maximum of 30% from peptide 1 to peptide 2, while the Pearson correlation of the drug/DMSO ratio was 0.6 (Fig. EV3D)."

The idea with such targeted PTM assays is that they should allow very consistent read out of phosphosites, be quite quantitative and ideally high throughput and easy to do. They sit between label-free assays and TMT-assays. Especially the label-free assays with DIA methods on high resolution instruments have been gaining ground, whereas the author's technology is quite mature and still uses low resolution instruments (triple quadrupoles). As the Carr group also performs dia, it would have been interesting to see a comparison between them, especially in quantitative accuracy and completeness.

- We thank the Reviewer for their comment and agree that such a comparison is warranted, but we think it is beyond the scope of this manuscript. We are in the process of assessing detectability of the targets using internal standard triggered PRM which is more sensitive than DIA in our hands and as reported by others (e.g., Schmidlin PMID: 27219855). This work will be described elsewhere. In our experience and that of others, the targeted MS assays using heavy-labeled internal standard peptides are not between label-free and TMT with respect to quantitative accuracy, but superior to both. The latter is subject to compression that can obscure the actual fold changes occurring as illustrated in Figure EV4B and described on p11. Please also see Figure 5 in PMID: 25724909 where this same effect is shown for another isobaric mass tag label, iTRAQ.

Overall, this is a substantial amount of work with many interesting aspects to it. The approach is not novel but has been under development for at least 10 years. Given the care that needs to be taken with the spike in peptides, an overall question would be if it is not easier to go with dia based method, which achieve very good CV values today. However, if the authors concentrated on the many points that are valuable to the community, this manuscript could still be a valuable addition to the literature.

- Our lab has considerable experience with DIA and a variation we call DIA-PRM (Peckner et al. Nat Methods 2018 PMCID: PMC5924490; Alvaro Sebastian Vaca Jacome et al. Nature Methods 2020 PMID: 33199889; Dele-Oni et al. Nature Scientific Data, 2021 in press.). We and others (e.g., PMID: 26060331) find that the CVs of DIA can be relatively poor unless using heavy-labeled internal standards. For example, see figure below and legend. We strongly believe there are important applications for DIA (especially DIA-PRM), but its sensitivity is not at the same level as MRM or PRM and only achieves excellent reproducibility when using internal standards. Our assay puts a premium on sensitivity over CV, although the CVs we achieve by MRM are quite good (<6%, see below).

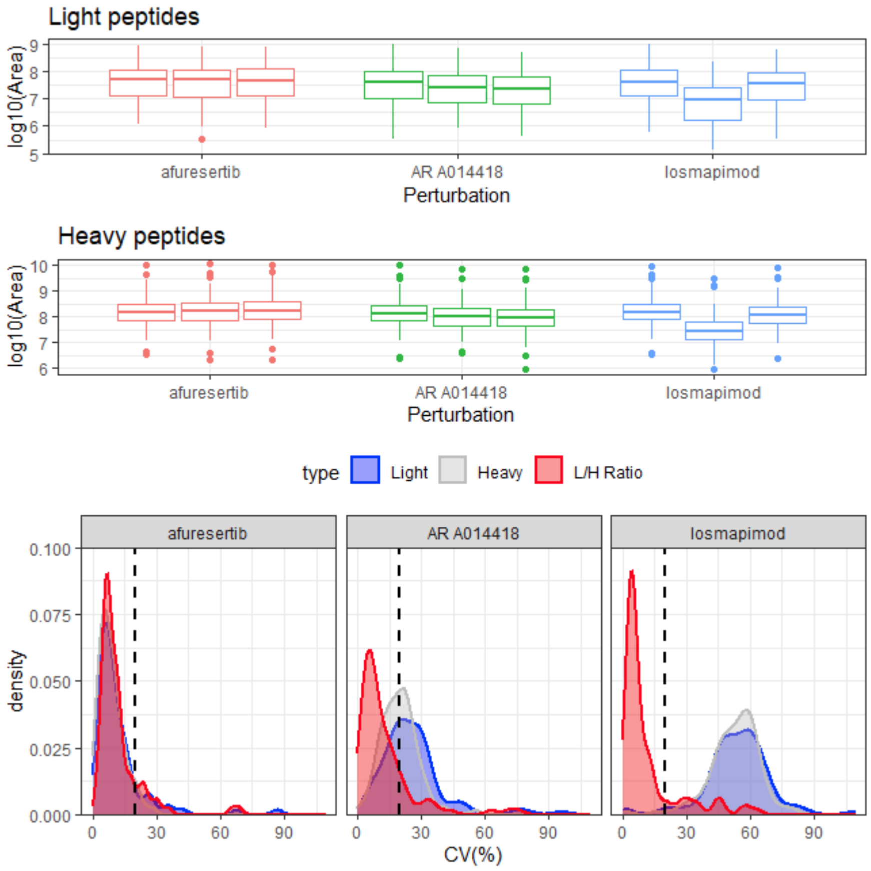

Figure Legend: Prostate cancer cell line (PC3) treated with three drugs/perturbations as shown. Data collected in DIA mode on Thermo HF instrument. The P100 heavy peptides (PMID: 26912667) were spiked in. The first two panels show the distribution of areas for the light (blue) and the heavy peptides (gray) for three drug perturbations analyzed in triplicates. The first drug perturbation (afuresertib) seems to have run without any problems and replicates are reproducible. The other two perturbations (AR compound and los compound) were not very reproducible. The third panel shows the distribution of CVs of all 96 peptides calculated on the areas for the light peptides, the areas of the heavy peptides, and the light-to-heavy ratios. For the first drug perturbation (afuresertib), the CV distributions are the same no matter what data we use. However, for the other two drug perturbations, without the use of heavy peptides the CVs are as large as 60% wheras using the L/H ratio corrects the variability of the measurement and gives excellent precision of quantification (Red curve mostly below a CV of 20%).

Organization of the paper: The authors should move a lot of the material from the back of the paper to the front, especially where they explain their assay, its limitations and its context. This would help the reader. They should also state in the main text that it is an MRM assay on a triple quadrupole. This would also be a good place to mention practical aspects like throughput (2-3h gradients?), what is necessary to create the library, costs, stability etc. They should also mention amounts needed, that the assay does not correct for protein level changes, quantitative accuracy expected and so on, in the beginning before they go to biological applications. The authors also claim 85% success rate but this can be much lower depending on the amount available, as they show later.

- The items requested by Reviewer are appropriately addressed in Discussion and Methods already, including how long the assays take to run, amounts of sample required, etc. As per reviewer's suggestion we added more detail to the "SigPath assay development" section about the library generation, as well as optimization of MRM parameters, and overall workflow (p7).
- Quantitative accuracy has been addressed in a new titration curve experiment performed during the revision of the manuscript summarized in the "SigPath assay configuration and evaluation" section (p8).

- The success rate of the assay was assessed based on the detection of spiked heavy peptides after the enrichment. Detection of endogenous signals is dependent on the presence and abundance of the phosphoproteins represented in the assay measured in a given study.

The authors cite their own efforts in MRM assay development and the CPTAC consortium extensively but are less inclusive in citing work outside. In the supplementary material, the authors say that all MS data was published in 2018, which is confusing.

- We thank the Reviewer and have now much more extensively and appropriately cited the important work of others in the introduction on pp4-5.

Specific points:

1. The title should be changed. E.g. Multiplexed phosphosite detection assay for...

- We respectfully disagree and prefer to keep the current title that appropriately emphasizes that this is a very large set of phosphosites being assayed.

2. Phosphosites of the assay (Figure 1C and 1D) should be depicted in a signaling pathway scheme.

- We have now added a new figure (Fig. 1B) that better illustrates the biology and pathways that are at least partially covered by the panel. The number of sites measured in each pathway is indicated graphically, and detailed information regarding pathways and coverage is provided in Dataset EV2 and figure EV1D.

3. Describe the assay in the main text (Figure 1A) and the figure legend has to be explained clearly.

- A brief description of the assay has been added to the “SigPath assay development” section of the manuscript (p7).

4. Conclusions drawn from the correlation in Figure 2B should be described in the main text.

- Figure 2B was mistakenly cited in the text as Figure 4B. We apologize and have now corrected this typo. Conclusions drawn from Figure 2B were already described in the result section. Please, refer to page 9.

5. Exchange Figure 4B on page 8 with Figure 2B.

- We thank the reviewer for noticing this and have corrected it.

6. Figure 2A legend contains the same sentence twice.

- Thank you for pointing this out. We have now fixed this.

7. 2C, 2D has to be better explained in the text. It is not clear that Y542 and Y580 are outliers in patient samples with ALK fusion; also, it looks like Figure 2C has a different pt of the lines than Figure 2D.

- We thank the reviewer for the close reading of the manuscript and review of the figures. We agree that the prior narrative was unclear, and in revision we have modified Figure 2B, removed Figure 2D, replaced Figure 2C, and expanded the associated text pertaining to the ALK-inhibitor treated cell line (main text P9 and Figures 2B and 2C).

8. Was the proteome of the cell line and PDX samples measured to check whether changes in phosphorylation underlie proteome changes?

In general, can authors also target nonphosphorylated peptides on the same proteins and measure their levels before enrichment to investigate whether changes in phosphosite levels can be just attributed to protein level differences instead of analyzing TMT proteome data (like done in Figure 4B)? Proteome data must also suffer from ratio compression.

- The cell line experiments were short-term perturbations and little if any change in proteome levels were observed over 6h. With respect to the PDX example provided (Mundt et al. *Cancer Res.* 2018), the abundance of each phosphosite was normalized to its corresponding protein abundance in the TMT analyses.
- Our assay is not designed to measure site stoichiometry, nor do we claim that it does. We often address this issue in our non-targeted work where one can normalize the phospho data by the protein levels if that is of interest and as was done in Figure 4. Creating a separate and parallel assay for the non-phosphorylated versions of each phosphopeptide can of course be done, but using these data to establish site stoichiometry is complicated if an enrichment step is used to detect and quantify low abundance phosphopeptides as was done here. If the phosphoprotein of interest is of sufficiently high abundance, one could develop an assay to begin to assess site stoichiometry by developing targeted assays to 1) an unmodified peptide to use as a surrogate of protein abundance, and 2) the unmodified version of the phosphopeptide. The sample would be analyzed +/- phosphatase treatment and the change in the abundance of the formerly phosphorylated peptide will provide the site stoichiometry. This requires that the unmodified peptides from the target protein are detectable in a single shot analysis, that the phosphatase reaction goes to completion and that the site stoichiometry is not very small. Assessing if the change in phosphorylation observed is more likely due to protein level change or site-specific phosphorylation change could, in principle, be accomplished by adding heavy non-phosphorylated peptide standards to the flow-through from the IMAC enrichment and measuring these non-phosphorylated peptides in a separate and parallel assay. The success of this method is entirely dependent on the ability to detect and quantify the unmodified peptide in the complex digest using a single shot approach. We have added the following two sentences to the Discussion (p14):
  "Assessing if the change in phosphorylation observed is more likely due to protein level change vs. site-specific phosphorylation change could, in principle, be accomplished by adding heavy non-phosphorylated peptide standards to the flow-through from the IMAC enrichment and measuring these non-phosphorylated peptides in a separate and parallel assay. The success of this method is entirely dependent on the ability to detect and quantify the unmodified peptide in the complex digest using a single shot approach."

  Of course, if no enrichment step is used, site stoichiometry can be obtained by spiking heavy-labeled versions of the base peptide and the phosphopeptide as the ionization efficiencies by electrospray are likely to be similar if not identical. This is a complicated issue and well described by Prus et al. (PMID: 31296352).
  Regardless, the overall amount of phosphorylation of a given site is still valuable despite its reflecting both the site-specific changes and the overall protein abundance. It is true that proteome data does suffer from compression, but is largely alleviated by use of extensive fractionation prior to on-line LC-MS/MS.

9. If a figure in the text is described, please mention the figure as well and make sure it is in the correct position, e.g. the description of Trametinib treatment outcome (Figure 2A) (page 9) or the description of the sensitivity for the PDX models (Figure 3A).

- We thank the reviewer for noticing this oversight and have fixed it now.

10. Figure legend for S3: The last sentence should be finished.

- This has also been fixed.

11. Figure 3C, 4A, S2A: What are the units/legends? Figure 3D, are fold changes on log2 scale?

- The heat maps in Figure 3C and 4A are colored with relative colors from blue to red per row, from row min to row max, respectively. This coloring scheme has been applied after the rows have been adjusted to robust Z-scores (subtracted median and divided by the median absolute deviation).
- The heat map in Figure S2A uses a relative color scheme from between 0 and 1 to depict the abundances of each site across 10 cell lines.
- The fold changes in Figure 3D are on log2 scale.
- This has now been clarified in the respective figure legends.

12. Were PTPN11 substrates measured? Are they differentially phosphorylated upon treatment or in cells harboring ALK fusions and EGFR mutants?

- Gab1 (GRB2 (growth factor receptor-bound protein 2)-associated binding protein 1), a multi-site docking protein that is both essential for PTPN11 function and regulated by PTPN11 activity, is among PTPN11 substrates that were measured. We have now highlighted this in Figure 2B, and added the following to the main text (p9):
  "Notably, we also observe downregulation of Gab1 phosphorylation at Y659 (Fig 2B), which is required for Gab1-PTPN11 binding and activation of downstream ERK/MAPK signaling initiated by PTPN11 (PMID: 11323411). Our current data showing that ALK inhibition leads to significant downregulation of phosphosites on both the C terminal of PTPN11 and Gab1 fits with these other lines of evidence in indicating a key and therapeutically tractable role of this phosphatase in ALK-mediated downstream signaling both in cell lines and human tumors."

13. In light of the amount of material required for phosphoenrichment, how practical is the approach for clinical/biological applications?

- The method is well suited for biological and clinical applications where available sample amounts are not limiting, such as for cell lines, surgically resected tumor tissue from humans and PDXs. Currently this version of the protocol is not suitable for clinical biopsies that typically yield <50ug protein amounts. This is not a limitation of this method per-se but rather of the enrichment reagent, primarily the pY antibody that is currently available. We are working to obtain a more sensitive version of pY affinity reagent that will likely make this method amenable to lower levels of sample input and allow this method to be fully automated.

14. Another limitation of their assay would be its throughput when it is applied to large sample collections. It utilizes and requires two laborious steps to enrich phosphopeptides to quantify them with high sensitivity. Furthermore, each LC-MRM/MS run takes considerable time (160min for IMAC method and 120min for pY method), allowing them to analyze only five samples per day. How possible is it to shorten gradients and simplify the workflow in future? This should be discussed in the manuscript in light of current developments in technology. Authors may consider revising their following sentence 'Sample preparation for SigPath is much simpler than methods commonly used

for deep-scale discovery...' as several streamlined phosphoproteomics platforms for in-depth analysis have been described in the literature.

- We agree with the reviewer and have modified text on pgs 14-15 in Discussion as follows: “While sample processing was done manually in the present study, throughput for SigPath can be greatly increased using automated digestion and IMAC enrichment on liquid handling robots as we have previously demonstrated (Abelin *et al.*, 2016). Antibody-based capture of pY-peptides will also become much faster and more reproducible once these antibodies are conjugated to magnetic beads for processing on systems like the Kingfisher as we have done in the case of KGG-peptide capture for ubiquitylation profiling (Rivera *et al*, 2021). The assay as presented here requires a total of 5 hours of on-instrument time for the analysis of both pY Ab- and IMAC-captured samples. This time can be shortened with faster MS instrumentation, use of shorter gradients or by mixing pY and IMAC captures and analyzing these together in a single LC-MRM/MS run. Use of FAIMS for posttranslationally-modified peptides would also provide another level of separation and potentially increase sensitivity (Popow *et al*, 2021; Udeshi *et al*, 2020).”
- We have also modified the sentence in question to read: “Sample preparation for SigPath is simple, requiring only digestion, phosphopeptide capture and analysis of the captured peptides together with spiked heavy peptide standards.…”

15. What is the assay quality without phosphoenrichment? Are enrichment procedures automated? If so, in which platform were they performed?

- Most of the phosphopeptides in the assay require deep profiling of the phosphoproteome with use of both fractionation and IMAC. Similarly, the majority of pY-peptides are not detected unless enriched using the antibody.

16. Figure legend 4B: was Pearson intended instead of Pierson correlation?

- Apologies for the typo, this has now been corrected.

17. Figure 4B: Is the fold change indicated? This should be added to the figure legend.

- This has now been clarified on the figure and in the figure legend.

18. The precision and reproducibility of the assay should be explored further; for instance, workflow and analytical variabilities can be determined.

- To address this comment we carried out titration curve experiments with both pY antibody and IMAC enrichment. The results for the IMAC subset of the assay (new Figure EV2B) show excellent CV (median of 5-10%) and linearity over a wide concentration range (0.05 - 1mg input protein). The pY subset has a median CV of 8-11% with protein input of 1 and 5mg (new Figure EV2A). We added experimental details to the methods section (p31), as well as “SigPath assay configuration and evaluation” section to the manuscript (p8).

19. Were SIL peptides AQUA peptides with known quantities? It is unclear if the authors determined their purity and asses light peptide contamination levels.

- All synthetic heavy isotope-labeled peptides (SIL peptides) were greater than 95% pure, and quantified by AAA analysis. These details have now been added to the methods section (p25).

20. Please read the manuscript carefully and remove all the typos.

- Done as requested.

Reviewer #2:

In this manuscript, Keshishian et al design a MS-based assay to systematically detect and quantify phosphorylation events in key regulatory proteins across a range of signaling pathways. They selected and curated a list of candidate peptides inferred from the literature, and combined this with available large-scale phosphoproteomics data to eventually compile an assay termed SigPath that spans 284 phosphosites in 200 phosphoproteins. They apply the assay to monitoring changes in the targeted phosphoproteome in cancer cells upon drug treatment, in cancer xenografts, and in tumor tissues. They identify phosphorylation events that were expected for the treatment or condition, in addition to novel ones that were unexpected and (in part) not detected in previous unbiased approaches. This leads the authors to conclude that SigPath should be a useful assay to examine the activation status of signaling pathways in various clinical and pre-clinical scenarios.

This is an interesting study with a clear rationale, namely that it may be more informative to accurately quantify a limited set of biologically important phosphorylation events, instead of doing this in a more classical unbiased way identifying thousands of phosphopeptides however whose function and relevance is less clear. Setting up such an assay is a considerable effort, requiring the selection of phosphopeptides (based on prior knowledge), optimization of MS conditions to reliably detect these peptides, and synthesis of isotope-coded versions of these peptides to be spiked into biological samples for quantification of endogenous phosphopeptides. Indeed, and rightfully, the description of the assay development takes a considerable portion of the manuscript.

Some critical issues remain as to the novelty of the manuscript, both conceptually using multiplexed MRM as an assay for cellular signaling activity, and with regard to biological findings. This paper is not the first to develop such an assay, two of which are referred to in the manuscript (Abelin et al, and Kennedy et al), where it remains a bit unclear what SigPath adds especially with regard to the Abelin tool (P100). Also there is some previous work that was not cited. With regard to the applications of SigPath that are shown, this reviewer had some mixed feelings about the few observations that were highlighted from the data set, against a range of results that remained uncommented. In particular, it is unclear why so little consistency was observed both in the drug-treated xenograft mice and among the sampled breast cancer phosphoproteomes. It either means that the assay is less conclusive than anticipated, or the diversity in signaling activity (both qualitatively and quantitatively) is much bigger than initially thought, which would be a sobering conclusion. Either way, it would have been advantageous if this had been exposed/investigated more deeply to point to potential weaknesses in the assay, or to challenges that to be addressed to increase our general understanding of cellular signaling.

- We thank the reviewer for their positive comments about the overall rationale and strategy employed in our study. We address the concerns raised below. We do believe that the results of

the assay that we present are more consistent with the notion that diversity in signaling activity (both qualitatively and quantitatively) is indeed larger than previously thought, although more work that is beyond the scope of this manuscript will be required to prove that conclusively.

Specific comments:
1. Abelin et al used a very similar strategy to design an MRM assay (termed P100, later used in a more elaborate application PMID 29655704) with a very similar target space as SigPath. How do P100 and SigPath differ, and why was it necessary to develop SigPath having P100 at hand? And why was it needed to build and optimize SigPath from scratch, having much of the foundation (probably some shared peptides/phospho-sites) already in place? What can SigPath do that P100 cannot?

- SigPath differs from the methodology and intent of P100 in two significant ways: P100 targets were selected based on a small number of initial chemical or genomic perturbations in cell lines, not based on their potential biological relevance. Furthermore, the targets chosen were high or relatively high abundance and could easily be measured by PRM - a key to why they were selected. In contrast, 80% of the phosphosites targeted by SigPath were nominated by cancer biologists for their relevance in known or suspected cancer biology of immediate use in understanding disease and impact of therapeutics, not based on prior observation in tumor tissue, PDX and cancer-relevant mammalian cell lines. Then the initial list of almost 400 sites was reduced to the final ca. 300 targets based on detection in deepscale phosphoproteomics experiments, not shallow dive phosphoproteomics as done in P100. In addition, only 1 peptide is in common between the P100 assay and SigPath.

- To clarify the distinction between SigPath and P100 further, we have added the following text to the introduction on p6: "... while Abelin et al. assayed a set of moderate-to-high abundance phosphopeptides known to be modulated in non-uniform ways by a panel of drugs in a range of cell lines. The targets were selected based on ease of detection in a small number of initial chemical or genomic perturbations in cell lines, not on their potential biological relevance. "

2. Apart from Abelin et al and Kennedy et al, Picotti et al were probably the first to introduce the concept of a MRM-based 'sentinel assay' 6 years ago (PMID: 25194849), and to implement this at a very similar scale as in this manuscript. In particular, it included 157 proteins and 152 phosphopeptides in a multiplexed fingerprint assay to report on the activity of 188 biological processes. Although this was performed in yeast, the study should be highlighted in the manuscript because conceptually it set the stage for SigPath.

- We agree with the Reviewer and apologize for the oversight. We have now added reference to this paper in the introduction (p5) as follows: "Soste and coworkers developed targeted MS assays to 152 phosphosites and 157 proteins in yeast that were culled from the literature to develop what they called "sentinel markers" to give biological insights (Soste et al. 2014)."

- We also added citations to this reference and others from the Picotti lab elsewhere in the introduction (pp4-5).

3. A main concern about the manuscript is that it treats data obtained by SigPath as individual observations, despite the fact that it was set up to obtain a system-wide perspective of signaling. In addition, a tool like SigPath provides the unique possibility to explore (causal) connectivity between regulatory events. Unfortunately, this was not attempted, and can be seen as a missed opportunity.

- We agree with the reviewer that not providing a system wide analysis of the SigPath results is definitely a missed opportunity. We have since explored connectivity using CausalPath analysis (causalpath.org) of all 3 datasets. We added one of the analyses from the 2hr drug/vehicle treatment of breast cancer xenograft tissue study to Figure 3 (3B), and we are providing a zip file for CausalPath analysis of all the datasets that will let readers further explore the data. While we can certainly generate figures for all the experiments to include in the appendix of the paper, we think that illustrating with one use case and providing means for readers to do further exploration is sufficient.

4. Even for the single observations of changes in protein phosphorylation the authors did not go into much (mechanistic) depth. For instance (page 9), they find that phosphorylation of RB1 changes upon MEK-inhibition by trametinib, and suggest this as a path how the drug regulates cell proliferation. This is not entirely unexpected, if not to say that it is very well established that this is one of the key ways how MEK drives proliferation (via CDK4/6, e.g. PMID 28127048)

- As per the reviewer's suggestion, we have extended the MEK/CKD1/RB1 focus in the revised manuscript and have included the reference indicated by the reviewer (p10). The biological data presented in this manuscript adequately serves as a proof-of-concept for the utility of the SigPath assay, and therefore, we have limited our commentary on mechanistic details.

5. To what extent is the assay portable to other mass spectrometric platforms beyond this particular TSQ Quantiva that was used? This is of particular importance since optimal collision energies may differ between mass spectrometers.

- The reviewer is raising an important point about transferability of this assay. We and others have shown that MRM assays are transferable between different laboratories and instruments with further optimization of instrument specific parameters such as fragment ions, CEs. The following sentence has been added to the Methods section (p26)
  "While we configured the assay on TSQ Quantiva MS, other triple quadrupole instruments can be used for this assay with further optimization of MS specific parameters for each instrument (Abbatiello *et al*, 2015; Kuhn *et al*, 2012)"

6. Figure 4A: Some samples show extremely high or extremely low phosphorylation levels across many proteins (blue and red vertical streaks in the plot). Can this be assigned to any particular status of the patient the sample originates from, and can something be learned from this? Or can it be excluded that this is a technical artifact (e.g. different sample amount, failing enrichment, age of the sample etc)?

- As noted by the reviewer, some of these samples seem to be globally hypo- or hyper-phosphorylated. This is due in part to an accentuation of the colors by robust z-scoring of the data (median-MAD normalization). This was not directly apparent in the figure legend, and we have now changed the expression legend from blue to red to read "min" and "max", as well as noting it directly in the legend on page 44. In the non-median-MAD normalized data,

this phenomenon is not discernible. Alternatively, by performing a median centering on a sample-by-sample basis to account for the possibility of different loading amounts, the striated pattern is reduced. However, since the SigPath assay is a small subset of carefully selected sites, we cannot be sure of the appropriateness of normalizing by sample/column, since we do not know whether those sites are representative of the entire phosphoproteome for each sample. Nevertheless, for comparison, and only for review purposes, please see the heat map below where samples have been normalized (median centered) both by column and sample.

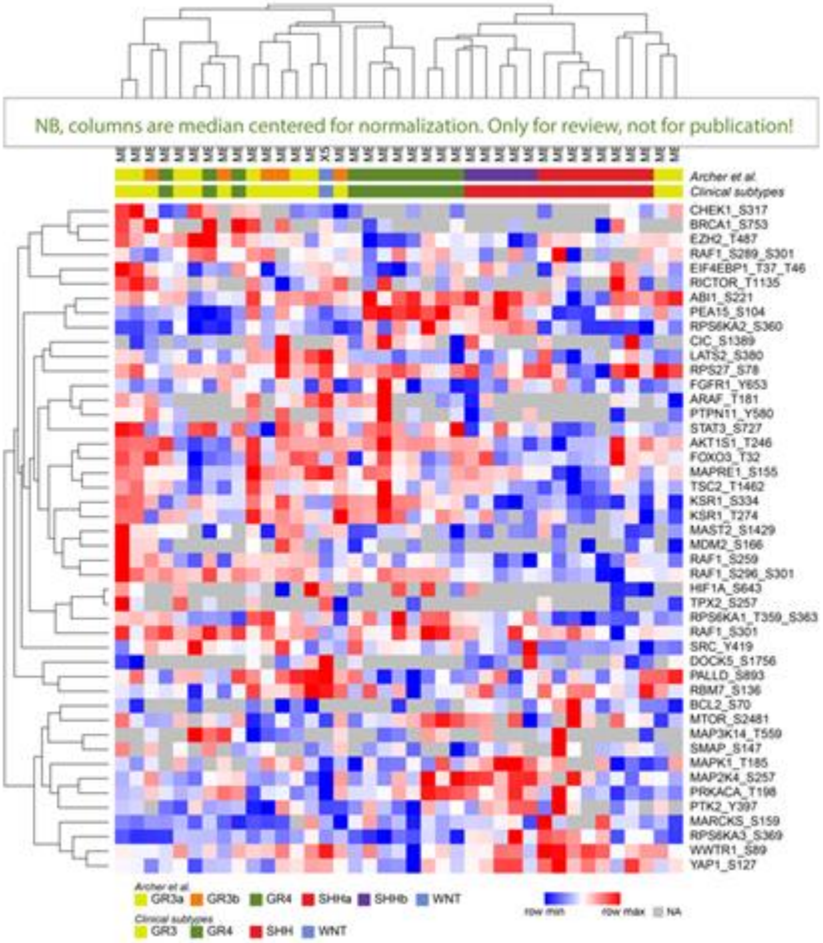

- The conclusions in the discovery data were based on an average of more than 35,000 phosphosites per patient. This discrepancy in the “field of view” will make any direct comparisons difficult. What the SigPath does beautifully is to reliably and with higher accuracy quantify a selected set of curated “cancer-relevant sites” which may not be enough to separate clinical subtypes. However, we do see a few of those markers being differential between the subtypes, and the high information content of the selected sites allows us to extract relevant biological information from individual samples. We have now performed the latter analyses in CausalPath and included them in the manuscript (see Data availability section of Methods for a link to it).

7. Fig 4A: Among the displayed proteins, the authors highlight YAP1 as a potentially interesting protein, representing one of few proteins showing consistent expression/phosphorylation in the

subgroups. For the remainder of the data, the general impression from the figure is that phosphorylation status is highly heterogeneous across these samples, and even within patient subgoups. Now with the notion that the sampled phosphorylation sites have been carefully selected to reflect biological activity, what does this mean for the signaling status of these patients/tissues, and the utility of (SRM-based) phosphoproteomics for their analysis/understanding? Should any of the conclusions of the discovery paper be revised (Archer et al, 2018), where different kinase activities were associated with disease subgroups?

- After data integration in Skyline we have now applied manual curation of the data for better assessment of detection of endogenous signals. For the peptides not detected we used imputation to assign a value for statistical tests. Details about this have been added to the methods section (pp32-33). After applying an unsupervised clustering (that was not performed initially) of the data the patient subgroups emerge - even in this limited dataset. It does, indeed, seem to follow the clinical subgroups. Something that the SigPath assay was not designed for, nevertheless, indicating a strong signaling of the underlying medulloblastoma subgroups.

8. In principle SigPath could have great potential, however it is under-used here. For instance, it would be great to plot the results of a SigPath analysis on the included pathways, visually connect observations, and identifies intermediate signaling hubs that may have been excluded from the panel but that may function as signaling hubs/branching points.

- We agree with the reviewer that not providing a system-wide analysis of the SigPath results is a missed opportunity. We have since explored connectivity using CausalPath analysis (causalpath.org) of all 3 datasets. We added one of the analyses from the 2hr drug/vehicle treatment of breast cancer xenograft tissue study to Figure 3 (3B), and we are providing a zip file for CausalPath analysis of all the datasets that will let readers do further exploration of the data. While we can certainly generate figures for all the experiments to include in the appendix of the paper, we think that illustrating with one use case and providing means for readers to do further exploration is sufficient.

9. PY-enrichment was not used in SigPath for the analysis of medulloblastoma tissue (p11 top of page). The authors should indicate how much material is needed for the assay, anticipating that the required input may be lower than for a regular phosphor-proteomics analysis benefiting from enhanced sensitivity afforded by MRM.

- We have now performed titration curve experiments for both pY and IMAC subsets of the assay, which in addition to studying the reproducibility of the assay also addressed the sensitivity achieved in detecting endogenous signals in a mixture of 5 cell lines. In this experiment we detected in the lowest point of the curve (1mg for pY, and 0.05mg for IMAC) 93 and 77 percent of the peptides detected in the highest input amount for pY and IMAC respectively. This detail now has been added to the methods section (p31) and SigPath assay configuration and evaluation section (p8) of the manuscript.

10. The discussion enumerates the conceptual advantages of SigPath, and MRM-based methods in general, to all of which I can agree. However it does not address the key question how its implementation works out in practice, and what can be learned from the data it delivers. For instance, by encompassing key nodes across a wide range of pathways and cellular processes, it should be possible to deduce (causal) relationships between changes in phosphorylation along or across pathways, instead of treating MRMs in the assay as individual measurements. The ability to do so

would be a distinct advantage of an integral assay such as SigPath over e.g. western blots that can test far fewer events simultaneously.

- This point is similar to point 8 raised by this reviewer. Again, we agree with the reviewer that not providing a system wide analysis of the SigPath results is definitely a missed opportunity, and we have done so using CausalPath analysis (causalpath.org) of all 3 datasets.

Reviewer #3:

Review of Keshishian et al., "Highly multiplexed quantitative phosphosite assay for biological and preclinical studies".

In this manuscript Keshishian and colleagues address an important issue with respect to cell signaling - namely the limited ability of most laboratories to profile more than a few biologically relevant signaling pathways at a time, using convenrtional approaches like Western blotting or other kinase assay-based techniques. While mass spectrometry has emerged as an extraordinarily useful approach to do this, most proteomics labs have concentrated their phosphoproteomic studies on accumulating as many substrates as possible rather than drilling down deeply into the underlying biology of a moderate but important set of known pathways and substrates. Some advances were made using reverse phase protein arrays, but this approach suffers from limited availability of high-quality antibodies, very limited dynamic range, and high background. One of the problems with most phosphoproteomic analysis platforms is the stochastic selection of peptides for analysis, and the complexity of the data, which limits direct comparisons between many samples. The authors build a collection of 298 phosphopeptides representing 284 phosphosites on 280 proteins, of which they appear to be able to detect ~60-70% reliably in subsequent experiments.

Major Comments:

1- Overall, the manuscript is more of a resource or technical article than a true description of new biology. Nevertheless, I think, if the appropriate limitations of the panel are adequately addressed in the text, it makes a very valuable contribution to systems-based studies of signaling, and is appropriate for Molecular Systems Biology.

- We thank the reviewer for their positive view of our work.

2- A major shortcoming of the manuscript is the complete absence of a comprehensive signaling diagram showing exactly which proteins and sites are being monitored with the technique. While the selection of phosphosites is, of course, a matter of personal choice, it would have made more sense to ensure that many of the key signaling pathways were represented. As best I can tell from the Supplemental data and Excel spreadsheet, the selection of pathways captures some, but not all, sites on proteins that are critically important in cell decision processes. For example, at least 5 sites on mTOR and half a dozen mTOR substrates are measured, but there are no phosphorylation sites on PI3KR1, DNA-PK, Cdc25A, B, or C, etc. As such, the panel seems to be heavily biased towards measuring signaling in the MAPK, mTOR and Hippo space, with some, but not nearly comprehensive elements of the PI-3K and apoptosis pathways, and other pathways much more lightly sampled. This needs to be explicitly shown using a pathway diagram, or series of pathway diagrams, so that the readers and adopters of the technology will know what is and is not adequately sampled.

- We agree with the reviewer that a comprehensive diagram was missing from the manuscript. We have now added a new figure 1B that shows all the proteins and the number of sites represented in SigPath from the Hallmark gene sets. Since it would be hard to show names of all the proteins in this diagram, we represented them with blue-colored boxes and used the intensity of the blue color to represent the number of sites per protein. In this figure we also added edges to show overlap between the different gene sets. We hope this diagram captures the assay as represented with Hallmark gene sets. Additional detail in list form is provided as Table 1 and Dataset EV2.

3- The statement protein "phosphosites were selected to provide readouts on DNA damage, cell cycle arrest, apoptosis, spindle checkpoint activation, hypoxia, autophagy, cell stress and epithelial-to-mesenchymal transition" seems to overstate the conclusions that can be drawn from what is actually being measured. To really understand the signaling in these pathways many additional components would need to be measured. The sentence should be revised to indicate the preliminary nature of the readouts that the panel provides. It is certainly an overstatement to claim that the readout of a single site on ATM and Chk1, for example, is an adequate proxy for reading out DNA damage and cell cycle arrest for example. Instead, the sites provide some preliminary information that could be used in subsequent experiments to focus on things like DNA damage, cell cycle arrest, etc. and this should be explicitly acknowledged.

We thank the reviewer and agree with this important comment. In the discussion on page 13 we have added the following to make the limitations clearer:
"It is important to note that the coverage of these pathways by the current SigPath assay is variable and incomplete, but the sites measured provide some preliminary information that can be used as starting points for further exploration. Furthermore, the assay can be expanded to include additional sites of interest."

4- The panel is then used to examine phosphorylation site changes at 2 time points - 6 and 24 hours in LUAD H1322 cells treated with the ALK inhibitor ceritinib and CRC Ls513cells treated with the MEK inhibitor Trametinib. The findings that the authors observe are consistent with what has been observed in CPTAC data from patients with these tumor types. However, other than validating that the panel can detect changes, we really learn almost nothing of biological consequence here. The authors should add a diagram mapping the ALK signaling pathway including PTPN11, and the

adjacent relevant pathway such as ERBB3, EGFR, and JAK-STAT, showing which sites downstream from ALK on which proteins were the most and least down and up-regulated, which would provide much more biological context for the results. For example, the finding that PTPN11 Y542/580 phosphorylation is reduced might be expected to result in reduced Erk phosphorylation (Miura et al, Oncogene, 2013), yet no such decrease in Erk phosphorylation is observed, possibly due to upregulation of ERBB3 and EGFR. This should be explained in a few more sentences, citing the appropriate references that support this. Can the authors provide some direct evidence of this by simply co-treating the cells with ceritinib plus and ERBB3/EGFR inhibitor and simply blotting for phosphoErk? This would markedly improve the manuscript, clearly extend the mass-spectrometry observations and confirm the tentative conclusions about activation of alternative Tyr kinase receptor pathways.

- We agree that one cannot confidently infer biological consequences based solely on the presented data, especially when dealing with something as complicated and nuanced as the interplay between multiple RTKs. This is not, however, the chief objective of this manuscript, which is instead focused on the methodology and upon use cases as proofs of concept for the ability of the assay to generate specific, testable hypotheses across a range of basic and translational applications. The findings put forward in that context require further experimental validation that is beyond the scope of this manuscript.
  We also thank the reviewer for pointing out the Miura, et al. *Oncogene* paper showing PTPN11 phosphorylation paralleled by ERK phosphorylation. Consistent with that report, we also observe clear downregulation of ERK1/2 (MAPK3/1) phosphorylation in the H1322 cell line when treated with the ALK inhibitor ceritinib. This downregulation was observed at both the 6 and 24 hour timepoints, but achieved statistical significance (FDR <0.05) only at the latter time point together with the PTPN11 pY sites as shown in Figure 2A and 2B. While, as noted above, complicated feedback mechanisms are likely to exist in these pathways, since in this case pERK is decreased, consistent with expectations, we did not think the proposed validation experiment was warranted.

5- The panel is then applied to six TNBC PDX model samples, with baseline values and values at 2 hours after a single dose, and at 50 hours after treatment with the PI-3K inhibitor buparlisib, aapparent 2 or 3 doses. These studies appear to have been done with one mouse/tumor from each of the PDX samples, but the caption to Figure 3A seems to indicate a total of 30 samples. Can the authors clarify this discrepancy? The data shown in Figure 3 indicates single values of phosphorylation - are these average values from multiple repeats, and if so, how much spread is there in the data?

- We are sorry for the confusion about the total number of samples analyzed for this study. Figure 3 shows 5 different PDX/mice models, each of which has undergone 5 different treatments (2 hours Buparlisib, 50 hour Buparlisib, washout, 2 hour vehicle and 50 hour vehicle). I.e, 6 models and 5 treatments each, in total amounting to 30 samples. We have now clarified this in the figure legend.

6- The main thing that one can conclude from this limited study is that the single most resistant PDX model appears to show the least effect of the drug on AKT phosphorylation at three well-established sites. In this regard, the statement "Importantly, the SigPath targeted MS data clearly shows quantitative differences in site modulation between the sensitive and resistant models upon inhibition" seems to distort and overstate the findings since there does not seem to be any obvious

correlation between the levels of phosphorylation inhibition following drug treatment of the sensitive versus resistant models except for the single most resistant one.

- We thank the reviewer for this thoughtful assessment, and agree that the impact of these findings was overstated. We have made appropriate changes to the language of the main text, which now focuses on the most resistant PDX model with respect to sites AKT1S1 pT246, AKT pT308 and AKT pS473. We now emphasize that these are established pharmacodynamic sites, not sites that we suggest to be response markers for PI3K inhibition, in less ambiguous text on page 10, paragraph 4, and on page 11, paragraph 1. Furthermore, our CasualPath analyses show a strong inhibition of PI3K related signaling at 2 hours, and less at 50 hours, in line with our discovery data (Mundt et al.). Our CasualPath analyses are now discussed on page 11, paragraph 1.

7- The other conclusion one can draw from this data is that the resistance of some of the PDXs to the PI-3K inhibitor is not mediated at the level of AKT phosphorylation, suggesting the development of resistance arises from other mechanisms, and perhaps this should be explicitly acknowledged.

- The conclusion reached by the reviewer that the observed resistance is not mediated exclusively via the classical PI3K pathway is precisely the one advanced in the discovery study, and we fully agree with the reviewer that it should be made explicit here. We appreciate the recommendation, and have made commensurate changes to the main text (page 11, paragraph 2).

8- Why is the phosphorylation status of MAPK3 not explicitly shown in Figure 3C, if its activity is what the authors claim is responsible for resistance of the W12 PDX sample?

- Figure 3C was originally based exclusively on PI3K/AKT/mTOR sites. The phosphorylation status of MAPK3/ERK1 (pY204) has now been added to figure 3C, and noted in the text.

  9- The authors then apply the IMAC approach to query medulloblastoma samples from 38/40 previously analyzed tumors. They focus the text in the results section on two very interesting molecules, YAP and TAZ. Here again, however, the lack of a deeper description of what the results mean, in light of known biology, limits the conclusions they draw. For example, the statement "The Yap1 protein is amplified and upregulated in hedgehog-associated medulloblastomas (Fernandez et al., 2009) while the quantified Yap1 pS127 site indicates inactivation of the protein in this subtype (Artinian et al., 2015)" is only partially correct. Phosphorylation of YAP at Ser-127, and TAZ at Ser-89 results in 14-3-3 binding and cytoplasmic sequestration of these molecules (c.f. Kanai et al. EMBO J, 2000, Zhao et al., Genes and Development 2007) and limits their ability to co-activate TEAD transcription factors that contribute to proliferation. I believe that clarifying and explaining this aspect of the findings provides the reader with a better understanding of what the mass-spec results might mean.

- We thank the reviewer for this observation, and for the specific guidance with respect to interpretation of some of the findings. In a systematic effort to more deeply probe our results in the context of known biology we have employed CausalPath analyses (described above) suggesting among other things activation of YES1, an upstream regulator of YAP1. We have

also availed ourselves of the reviewer's expertise in directly incorporating the insights regarding 14-3-3 binding, sequestration and the consequent limitations on TEAD transcription factor co-activation (PP 12-13).

Minor comments

10- The fact that the pY and IMAC mixtures are spiked with labelled peptides should be mentioned early in the Results section.

- This detail has now been added to the SigPath assay development section

11- Can the authors comment on whether the panel should be optimally used with some type of isobaric labelling technique for serial time-sampled specimens in future studies?

- We are strong advocates for use of isobaric labeling combined with off-line fractionation pre-LC-MS/MS analyses as these provide very broad and deep coverage of proteome, phosphoproteome, etc. in serial time course analyses (e.g., drug perturbations in cell line experiments). However, these experiments require expensive labeling and many, many hours of on-instrument time. The SigPath assay can provide relevant (albeit limited) pathway modulation information in 7-10-fold less time than required by discovery experiments, and the quantitative precision of the measurements is much higher than for label-free, DIA or TMT-based methods.
- As far as actually combining the panel of labeled peptides with an isobaric labeling strategy, this can be done, but it carries the complication of 1) having to TMT label e.g., with superheavy version of TMT and 2) using, for example, the TOMAHAQ method (Erickson et al, PMID: 28065596). We have tested the TOMAHAQ method and find it to be far more complicated to implement than the approach we have presented here. That said, while such a comparison may be valuable, it is beyond the scope of the present paper. The SigPath assay as is can be implemented on triple quadrupole instruments as well as non-tribrid instruments like the Exploris, whereas one needs a much more expensive and complicated instrument capable of MS3 like the Lumos to use TOMAHAQ.

6th Aug 2021

RE:MSB-2020-10156R,Highly multiplexed quantitative phosphosite assay for biology and preclinical studies

Thank you for sending us your revised manuscript.We have now heard back from the three reviewers who were asked to evaluate your study.The reviewers are satisfied with the modifications made and they are supportive of publication.They only list a few remaining minor concerns,which we would ask you to address in a minor revision.

Reviewer #1:

In this revision, Keshishian et al. have addressed most of the points that they could address and we think that the revised version looks fine in general. The main point remains that there is little novelty or exciting biology in this paper but there is little to do about this at this point.

The main focus of the presented study is to develop a targeted MS assay for quantifying phosphosites of biological interest. Therefore, it was essential to demonstrate the robustness and precision of the assay.
The authors now support their case better by:

1. substantially expanding the discussion on practical aspects like throughput and potential limitations of their assay,
2. adding more detail to the results and method sections about how the assay was established,
3. and more importantly, performing additional experiments to show the precision and reproducibility of phosphopeptide detection.

Additionally, they provided use cases as proofs of concepts to show how this assay can potentially be utilized and be helpful in biological and preclinical studies. One example (Figure 3B) shows connections between regulated proteins by applying CausalPath analysis.

They also appropriately toned down their claims, especially regarding the capability of their assay and biological findings, and are now more inclusive in citing work outside - although this is still incomplete. Other acquisition techniques and global phosphoproteome pipelines should be acknowledged more, in our opinion. In Figure 1A, IMAC enrichment was described as antibody enrichment. This should be corrected.

Reviewer #2:

I thank the authors for their response, appropriately addressing my concerns, or providing

explanations when not all suggestions could be met. From the revised manuscript and the questions that were raised by all three reviewers a number of things have become clear: First, there is an undeniable need for an assay that quantitatively maps signaling activity across multiple pathways, and an MRM-based approach is arguably the best way to go. Yet, however easy it is to ask such a question, its implementation is utterly challenging in many respects. Exposing these challenges and providing solutions to them are the strengths of this paper, but also illustrating limitations (better done in the revision than in the original manuscript) is not a weakness, but in fact gains credibility by the displayed technical rigor.

Second, the ability to quantify a targeted set of phosphorylation events does not necessarily make it easier to infer biological regulation, as pointed out by Reviewer 3. Toning down the biological implications of the work with a more explicit focus on method development is a logical consequence. The notion that this type of analyses not only answers questions, but may also (or even primarily) raise new hypotheses for further investigation can be regarded as another take-away from this study.

Third, SigPath is claimed as 'highly multiplexed', however the chosen panel of targets currently represents only a minor segment of the cellular signaling space. Hence, the impact of SigPath can only grow by expanding the assay to additional phosphosites, proteins and pathways. This may be best done in a community effort, potentially following the technical standard as described in the current paper.

Overall, the revised manuscript demonstrates the potential of SigPath in biological and (pre)clinical research, and I therefore recommend publication of this exciting work.

Reviewer #3:

The authors have adequately addressed the majority of my concerns. Importantly, many of the overstated claims that were not fully supported by the data have been removed. Whilst I would have preferred that they experimentally test one of the conclusions about pathway rewiring after ALK inhibition, I concede that the value of the paper is more about the method at this point than the underlying biology. The addition of the pathway diagrams into Figure 1 is a major improvement. The more cautious discussion adds to the scholarship of the presentation. Other than some typos in the last sentence on page 10, the paper reads better than the original version. I believe the paper merits publication.

Reviewer #1:

In this revision, Keshishian et al. have addressed most of the points that they could address and we think that the revised version looks fine in general. The main point remains that there is little novelty or exciting biology in this paper but there is little to do about this at this point.

The main focus of the presented study is to develop a targeted MS assay for quantifying phosphosites of biological interest. Therefore, it was essential to demonstrate the robustness and precision of the assay.
The authors now support their case better by:
1. substantially expanding the discussion on practical aspects like throughput and potential limitations of their assay,
2. adding more detail to the results and method sections about how the assay was established,
3. and more importantly, performing additional experiments to show the precision and reproducibility of phosphopeptide detection.

Additionally, they provided use cases as proofs of concepts to show how this assay can potentially be utilized and be helpful in biological and preclinical studies. One example (Figure 3B) shows connections between regulated proteins by applying CausalPath analysis.

They also appropriately toned down their claims, especially regarding the capability of their assay and biological findings, and are now more inclusive in citing work outside - although this is still incomplete. Other acquisition techniques and global phosphoproteome pipelines should be acknowledged more, in our opinion.

We thank the Reviewer for positive comments about the revised manuscript. While we appreciate and share the Reviewer's view that other technologies (global and targeted via use of antibodies) have been and are being actively used for phosphoproteome analysis, and indeed have referenced a number of those, our paper is focused entirely on the development and application of a targeted MS assay and is not intended as a review of the field.

In Figure 1A, IMAC enrichment was described as antibody enrichment. This should be corrected.

We thank the reviewer for catching this oversight and have now corrected it.

Reviewer #2:

I thank the authors for their response, appropriately addressing my concerns, or providing explanations when not all suggestions could be met. From the revised manuscript and the questions that were raised by all three reviewers a number of things have become clear: First, there is an undeniable need for an assay that quantitatively maps signaling activity across multiple pathways, and an MRM-based approach is arguably the best way to go. Yet, however easy it is to ask such a question, its implementation is utterly challenging in many respects.

Exposing these challenges and providing solutions to them are the strengths of this paper, but also illustrating limitations (better done in the revision than in the original manuscript) is not a weakness, but in fact gains credibility by the displayed technical rigor.

Second, the ability to quantify a targeted set of phosphorylation events does not necessarily make it easier to infer biological regulation, as pointed out by Reviewer 3. Toning down the biological implications of the work with a more explicit focus on method development is a logical consequence. The notion that this type of analyses not only answers questions, but may also (or even primarily) raise new hypotheses for further investigation can be regarded as another take-away from this study.

Third, SigPath is claimed as 'highly multiplexed', however the chosen panel of targets currently represents only a minor segment of the cellular signaling space. Hence, the impact of SigPath can only grow by expanding the assay to additional phosphosites, proteins and pathways. This may be best done in a community effort, potentially following the technical standard as described in the current paper.

Overall, the revised manuscript demonstrates the potential of SigPath in biological and (pre)clinical research, and I therefore recommend publication of this exciting work.

We thank the reviewer for appreciating the importance of the enterprise, the complexity of its implementation, and the strong foundation that SigPath represents. We are glad our efforts in revision were found to be satisfactory, and reiterate that in responding to all three reviewers we think the manuscript was considerably strengthened.

Reviewer #3:

The authors have adequately addressed the majority of my concerns. Importantly, many of the overstated claims that were not fully supported by the data have been removed. Whilst I would have preferred that they experimentally test one of the conclusions about pathway rewiring after ALK inhibition, I concede that the value of the paper is more about the method at this point than the underlying biology. The addition of the pathway diagrams into Figure 1 is a major improvement. The more cautious discussion adds to the scholarship of the presentation. Other than some typos in the last sentence on page 10, the paper reads better than the original version. I believe the paper merits publication.

We thank the reviewer for the positive feedback, for recognizing the centrality of Sigpath assay development and application to this manuscript, and for guidance in ensuring a suitably measured exposition. We did not identify typographical errors on the last sentence of page 10; however we further refined the sentence to improve clarity. It now reads “Inhibition of these phosphorylation…”.

2nd Revision - Editorial Decision 20th Aug 2021

Thank you again for sending us your revised manuscript. We are now satisfied with the modifications made and I am pleased to inform you that your paper has been accepted for publication.

EMBO PRESS

YOU MUST COMPLETE ALL CELLS WITH A PINK BACKGROUND ➔

PLEASE NOTE THAT THIS CHECKLIST WILL BE PUBLISHED ALONGSIDE YOUR PAPER

Corresponding Author Name: Hasmik Keshishian
Journal Submitted to: Molecular Systems Biology
Manuscript Number: MSB-2020-10156R

Reporting Checklist For Life Sciences Articles (Rev. June 2017)

This checklist is used to ensure good reporting standards and to improve the reproducibility of published results. These guidelines are consistent with the Principles and Guidelines for Reporting Preclinical Research issued by the NIH in 2014. Please follow the journal's authorship guidelines in preparing your manuscript.

A- Figures
1. Data
The data shown in figures should satisfy the following conditions:

- the data were obtained and processed according to the field's best practice and are presented to reflect the results of the experiments in an accurate and unbiased manner.
- figure panels include only data points, measurements or observations that can be compared to each other in a scientifically meaningful way.
- graphs include clearly labeled error bars for independent experiments and sample sizes. Unless justified, error bars should not be shown for technical replicates.
- if n< 5, the individual data points from each experiment should be plotted and any statistical test employed should be justified
- Source Data should be included to report the data underlying graphs. Please follow the guidelines set out in the author ship guidelines on Data Presentation.

2. Captions

Each figure caption should contain the following information, for each panel where they are relevant:

- a specification of the experimental system investigated (eg cell line, species name).
- the assay(s) and method(s) used to carry out the reported observations and measurements
- an explicit mention of the biological and chemical entity(ies) that are being measured.
- an explicit mention of the biological and chemical entity(ies) that are altered/varied/perturbed in a controlled manner.

- the exact sample size (n) for each experimental group/condition, given as a number, not a range;
- a description of the sample collection allowing the reader to understand whether the samples represent technical or biological replicates (including how many animals, litters, cultures, etc.).
- a statement of how many times the experiment shown was independently replicated in the laboratory.
- definitions of statistical methods and measures:
  - common tests, such as t-test (please specify whether paired vs. unpaired), simple χ2 tests, Wilcoxon and Mann-Whitney tests, can be unambiguously identified by name only, but more complex techniques should be described in the methods section;
  - are tests one-sided or two-sided?
  - are there adjustments for multiple comparisons?
  - exact statistical test results, e.g., P values = x but not P values < x;
  - definition of 'center values' as median or average;
  - definition of error bars as s.d. or s.e.m.

Any descriptions too long for the figure legend should be included in the methods section and/or with the source data.

USEFUL LINKS FOR COMPLETING THIS FORM

http://www.antibodypedia.com
http://1degreebio.org
http://www.equator-network.org/reporting-guidelines/improving-bioscience-research-repor
http://grants.nih.gov/grants/olaw/olaw.htm
http://www.mrc.ac.uk/Ourresearch/Ethicsresearchguidance/Useofanimals/index.htm
http://ClinicalTrials.gov
http://www.consort-statement.org
http://www.consort-statement.org/checklists/view/32-consort/66-title
http://www.equator-network.org/reporting-guidelines/reporting-recommendations-for-tumo
http://datadryad.org
http://figshare.com
http://www.ncbi.nlm.nih.gov/gap
http://www.ebi.ac.uk/ega
http://biomodels.net/
http://biomodels.net/miriam/
http://jjj.biochem.sun.ac.za
http://oba.od.nih.gov/biosecurity/biosecurity_documents.html
http://www.selectagents.gov/

In the pink boxes below, please ensure that the answers to the following questions are reported in the manuscript itself. Every question should be answered. If the question is not relevant to your research, please write NA (non applicable). We encourage you to include a specific subsection in the methods section for statistics, reagents, animal models and human subjects.

B- Statistics and general methods

Please fill out these boxes ➔ (Do not worry if you cannot see all your text once you press return)

1.a. How was the sample size chosen to ensure adequate power to detect a pre-specified effect size?	NA, an effect size was not pre-specified
1.b. For animal studies, include a statement about sample size estimate even if no statistical methods were used.	All available PDX models were used. See Mundt et al 2018 (PMID: XX) for details.
2. Describe inclusion/exclusion criteria if samples or animals were excluded from the analysis. Were the criteria pre-established?	NA, no PDX models were excluded from the analysis
3. Were any steps taken to minimize the effects of subjective bias when allocating animals/samples to treatment (e.g. randomization procedure)? If yes, please describe.	NA, all PDX models underwent the same treatment protocol.
For animal studies, include a statement about randomization even if no randomization was used.	As all PDX models were subjected to treatment, no randomization was used.
4.a. Were any steps taken to minimize the effects of subjective bias during group allocation or/and when assessing results (e.g. blinding of the investigator)? If yes please describe.	no
4.b. For animal studies, include a statement about blinding even if no blinding was done	no blinding
5. For every figure, are statistical tests justified as appropriate?	Yes
Do the data meet the assumptions of the tests (e.g., normal distribution)? Describe any methods used to assess it.	We visually inspected the density of the distributions and confirmed that they appeared approximately normal-like; the moderated t-test we used is relatively robust, and can be applied to approximately normally distributed data.

Is there an estimate of variation within each group of data?	Yes
Is the variance similar between the groups that are being statistically compared?	Yes

C- Reagents

6. To show that antibodies were profiled for use in the system under study (assay and species), provide a citation, catalog number and/or clone number, supplementary information or reference to an antibody validation profile. e.g., Antibodypedia (see link list at top right), 1DegreeBio (see link list at top right).	Phospho-Tyrosine (P-Tyr-1000) MultiMab™ Rabbit mAb mix #8954 (CST)<br>Schlessinger, J. (2000) Cell 103, 211-25.
7. Identify the source of cell lines and report if they were recently authenticated (e.g., by STR profiling) and tested for mycoplasma contamination.	CCLE (Cancer Cell Line Encyclopedia)<br>https://portals.broadinstitute.org/ccle<br>All the cell lines have been mycoplasma tested prior to release for research use.

* for all hyperlinks, please see the table at the top right of the document

D- Animal Models

8. Report species, strain, gender, age of animals and genetic modification status where applicable. Please detail housing and husbandry conditions and the source of animals.	Patient-derived xenograft (PDX) models were selected from the Washington University Human in Mouse (WHIM) PDX collection as described in Mundt et al.(Mundt et al., 2018)
9. For experiments involving live vertebrates, include a statement of compliance with ethical regulations and identify the committee(s) approving the experiments.	NA
10. We recommend consulting the ARRIVE guidelines (see link list at top right) (PLoS Biol. 8(6), e1000412, 2010) to ensure that other relevant aspects of animal studies are adequately reported. See author guidelines, under 'Reporting Guidelines'. See also: NIH (see link list at top right) and MRC (see link list at top right) recommendations. Please confirm compliance.	Yes

E- Human Subjects

11. Identify the committee(s) approving the study protocol.	Ethics Committee of the Medical Faculty at Heidelberg University
12. Include a statement confirming that informed consent was obtained from all subjects and that the experiments conformed to the principles set out in the WMA Declaration of Helsinki and the Department of Health and Human Services Belmont Report.	Patient samples were obtained with informed consent according to the International Cancer Genome Consortium (ICGC) guidelines as approved by the Ethics Committee of the Medical Faculty at Heidelberg University, and as approved by the institutional review board of contributing center Nikolay Nilovich Burdenko Neurosurgical Institute in Moscow
13. For publication of patient photos, include a statement confirming that consent to publish was obtained.	NA
14. Report any restrictions on the availability (and/or on the use) of human data or samples.	NA
15. Report the clinical trial registration number (at ClinicalTrials.gov or equivalent), where applicable.	NA
16. For phase II and III randomized controlled trials, please refer to the CONSORT flow diagram (see link list at top right) and submit the CONSORT checklist (see link list at top right) with your submission. See author guidelines, under 'Reporting Guidelines'. Please confirm you have submitted this list.	NA
17. For tumor marker prognostic studies, we recommend that you follow the REMARK reporting guidelines (see link list at top right). See author guidelines, under 'Reporting Guidelines'. Please confirm you have followed these guidelines.	NA

F- Data Accessibility

18: Provide a "Data Availability" section at the end of the Materials & Methods, listing the accession codes for data generated in this study and deposited in a public database (e.g. RNA-Seq data: Gene Expression Omnibus GSE39462, Proteomics data: PRIDE PXD000208 etc.) Please refer to our author guidelines for 'Data Deposition'.<br><br>Data deposition in a public repository is mandatory for:<br>a. Protein, DNA and RNA sequences<br>b. Macromolecular structures<br>c. Crystallographic data for small molecules<br>d. Functional genomics data<br>e. Proteomics and molecular interactions	Data availability section has been added to the Materials and Methods with links to the raw data in Panorama, statistical analysis in Protigy and CausalPath analysis.
19. Deposition is strongly recommended for any datasets that are central and integral to the study; please consider the journal's data policy. If no structured public repository exists for a given data type, we encourage the provision of datasets in the manuscript as a Supplementary Document (see author guidelines under 'Expanded View' or in unstructured repositories such as Dryad (see link list at top right) or Figshare (see link list at top right).	NA
20. Access to human clinical and genomic datasets should be provided with as few restrictions as possible while respecting ethical obligations to the patients and relevant medical and legal issues. If practically possible and compatible with the individual consent agreement used in the study, such data should be deposited in one of the major public access-controlled repositories such as dbGAP (see link list at top right) or EGA (see link list at top right).	NA
21. Computational models that are central and integral to a study should be shared without restrictions and provided in a machine-readable form. The relevant accession numbers or links should be provided. When possible, standardized format (SBML, CellML) should be used instead of scripts (e.g. MATLAB). Authors are strongly encouraged to follow the MIRIAM guidelines (see link list at top right) and deposit their model in a public database such as Biomodels (see link list at top right) or JWS Online (see link list at top right). If computer source code is provided with the paper, it should be deposited in a public repository or included in supplementary information.	NA

G- Dual use research of concern

22. Could your study fall under dual use research restrictions? Please check biosecurity documents (see link list at top right) and list of select agents and toxins (APHIS/CDC) (see link list at top right). According to our biosecurity guidelines, provide a statement only if it could.	No
